# Transcriptome Comparisons Identify New Cell Markers for Theca Interna and Granulosa Cells from Small and Large Antral Ovarian Follicles

**DOI:** 10.1371/journal.pone.0119800

**Published:** 2015-03-16

**Authors:** Nicholas Hatzirodos, Katja Hummitzsch, Helen F. Irving-Rodgers, Raymond J. Rodgers

**Affiliations:** 1 Discipline of Obstetrics and Gynaecology, School of Paediatrics and Reproductive Health, Robinson Research Institute, University of Adelaide, Adelaide, South Australia, 5005, Australia; 2 School of Medical Science, Griffith University, Gold Coast, 4222, Queensland, Australia; China Agricultural University, CHINA

## Abstract

In studies using isolated ovarian granulosa and thecal cells it is important to assess the degree of cross contamination. Marker genes commonly used for granulosa cells include *FSHR*, *CYP19A1* and *AMH* while *CYP17A1* and *INSL3* are used for thecal cells. To increase the number of marker genes available we compared expression microarray data from isolated theca interna with that from granulosa cells of bovine small (n = 10 for both theca and granulosa cells; 3-5 mm) and large (n = 4 for both theca and granulosa cells, > 9 mm) antral follicles. Validation was conducted by qRT-PCR analyses. Known markers such as *CYP19A1*, *FSHR* and *NR5A2* and another 11 genes (*LOC404103*, *MGARP*, *GLDC*, *CHST8*, *CSN2*, *GPX3*, *SLC35G1*, *CA8*, *CLGN*, *FAM78A*, *SLC16A3*) were common to the lists of the 50 most up regulated genes in granulosa cells from both follicle sizes. The expression in theca interna was more consistent than in granulosa cells between the two follicle sizes. Many genes up regulated in theca interna were common to both sizes of follicles (*MGP*, *DCN*, *ASPN*, *ALDH1A1*, *COL1A2*, *FN1*, *COL3A1*, *OGN*, *APOD*, *COL5A2*, *IGF2*, *NID1*, *LHFP*, *ACTA2*, *DUSP12*, *ACTG2*, *SPARCL1*, *FILIP1L*, *EGFLAM*, *ADAMDEC1*, *HPGD*, *COL12A1*, *FBLN5*, *RAMP2*, *COL15A1*, *PLK2*, *COL6A3*, *LOXL1*, *RARRES1*, *FLI1*, *LAMA2*). Many of these were stromal extracellular matrix genes. *MGARP*, *GLDC*, *CHST8*, *GPX3* were identified as new potential markers for granulosa cells, while *FBLN5*, *OGN*, *RAMP2* were significantly elevated in the theca interna.

## Introduction

It is well known that ovarian follicles are formed during fetal development and initially are composed of an oocyte, arrested in meiosis and surrounded by a single layer of granulosa cells all enveloped by the follicular basal lamina [1]. Each day a number of follicles are activated to resume growth and development [2]. The granulosa cells replicate and eventually a fluid-filled antral cavity develops. It is surrounded by multiple layers of epithelial granulosa cells. Specialized stromal layers, the theca interna and externa, develop outside of the follicular basal lamina. The theca interna is composed of capillaries, fibroblasts, immune cells and specialized steroidogenic cells [3]. These steroidogenic cells produce androgens and also insulin-like-3 (*INSL3*). This commences when the theca interna is first recognizable as a distinct tissue layer and continues up until ovulation of the follicle [4]. The granulosa cells only mature into steroidogenic cells during the last 5% of their follicular development in the weeks leading up to ovulation [5]. At this stage, the granulosa cells develop the capacity to convert the thecal-derived androgens into estrogens. This splitting of the steroidogenic pathway between the thecal cells and the granulosa cells is known as the two cell theory of estradiol production [6].

Much of our knowledge of both theca and granulosa cells comes from analysis of isolated cells and their *in vitro* culture. The isolation typically involves extruding granulosa cells from ruptured follicles in small animal species or physically scraping them from the follicular basal lamina in follicles that have been split open in large animal species. If these processes are conducted carefully very little contamination of the granulosa cells with thecal cells occurs. Also, if not conducted thoroughly then the isolated cells are not representative of the *in vivo* cells as the antrally-situated cells can have different properties to those located basally in the membrana granulosa [7]. Additionally, the theca interna layer can be isolated by removal of the granulosa cells and dissection of the theca interna away from the externa. Cross contamination of granulosa cells with cells from the theca interna and *vice versa* is always a concern and is often confirmed retrospectively by quantitation of markers for the theca interna and for granulosa cells (examples include [8–12]).

To identify granulosa cell contamination of the theca cell preparations expression of cytochrome P450aromatase or its encoding gene *CYP19A1* and FSH receptor (*FSHR*) are often used. The standard marker of thecal contamination of granulosa cell preparations is cytochrome P450 17α-hydroxylase or its encoding gene *CYP17A1*. Insulin like 3 (*INSL3*) is also exclusively expressed in the steroidogenic cells of the theca interna [13] and is an appropriate thecal cell marker. There is definitely a need for identifying additional markers of granulosa cells as aromatase/*CYP19A1* is up regulated in large antral follicles [5], reducing its utility when working with much smaller antral follicles. Furthermore in very large follicles approaching ovulation, *CYP19A1* expression is reduced like during early atresia [5], which is a disadvantage when working with these follicle types. In follicles aromatase is exclusively expressed in granulosa cells, excluding suiform species [14], but its changing levels of expression during follicle growth and atresia makes it less than ideal as a marker. Thus additional markers of both theca and granulosa cells would be useful to have.

The goal of the current study was to examine the transcriptomes of granulosa cells and theca interna derived from both small (3–5 mm) and large (> 9 mm) healthy bovine follicles and to identify additional genes differentially expressed between granulosa cells and theca interna. The transcriptome analyses used the same type of Affymetrix platforms for all samples examined as recently published by us [15–18].

## Materials and Methods

### Tissues and cells, RNA extraction and array hybridization

Granulosa cells and intact theca interna were previously isolated from four different groups of individual healthy follicles (n = 10 for theca interna and n = 10 for granulosa cells from follicles 3–5 mm, n = 4 from theca interna and n = 4 from granulosa cells from follicles > 9 mm) obtained from an abattoir according to previously described methods [16,17]. The health status of the follicles was confirmed by histological examination of a portion of the follicle wall. It was based on the number of apoptotic figures observed in the membrana granulosa [19,20]. RNA was extracted from granulosa cells and theca interna of each follicle preparation and 2 μg of RNA per sample was processed for hybridization to Bovine Affymetrix Genome Array (Australian Genomics Research Facility, Parkville, Australia and ACRF Cancer Genomics facility, Adelaide, Australia) [16,17]. The CEL files can be obtained from the Gene Expression Omnibus (GEO) under series records GSE39589 and GSE49505 for granulosa and theca interna data, respectively.

### Array analyses

Affymetrix CEL file data were pre-processed in Partek Genomics Suite (version beta 6.6, St Louis, Missouri, USA) using RMA [21] background summarization, with quantile normalization and log base 2 transformation and mean probe set summarization with adjustment for GC content. Spike-in hybridization intensities on all arrays were within acceptable quality control limits.

### Statistical analyses

One-way ANOVA using method of moments estimation was conducted to determine the differentially-expressed probe sets between group comparisons. Post-hoc testing for multiple corrections was performed using the step-up Benjamini-Hochberg False Discovery Rate (FDR) method. *P* values less than 0.05 were considered to be statistically significant. Probe sets which were 4-fold different between granulosa cells and theca interna in small and large antral follicles were selected as differentially regulated. Intensity values for a unique probe set were assigned to each gene on the basis of maximum average intensity across all the arrays. Only genes annotated under Release 34 of the Affymetrix annotations of the Bovine Genome Array (Release Date, 10/24/13) were included in the lists of differentially-regulated genes.

### Measurement of gene expression by quantitative real-time polymerase chain reaction (qRT-PCR)

Total RNA (200 ng) from granulosa cells and theca interna of small (n = 10 each) and large (n = 5 and 4, respectively, and all from different animals) healthy follicles was extracted and used to synthesize cDNA as detailed previously [18]. All samples for qRT-PCR were identical to the samples used for the microarray apart from small follicle granulosa cells where limited amounts of RNA were obtained and additional small healthy follicles (n = 10) were obtained to isolate more RNA. In addition, an extra sample was included in the large follicle granulosa cell group for the PCR analyses (thus n = 5). Real time RT-PCR assays were designed against 11 genes using web based software and quantitative RT-PCR values were determined from the geometric mean of 2^-ΔΔCt^ of the target genes to the cyclophilin A (*PPIA*) and glyceraldehyde phosphate dehydrogenase (*GAPDH*) as performed previously [18]. The sequence information of the primers is shown in Table 1 and the genes were chosen to represent some genes known to be differentially expressed and other genes with diverse cell functions.

**Table 1:** Primer sequences used for qRT-PCR.

Gene Name	Gene Symbol	GenBank Accession No.	Forward primer (5’- 3’)	Reverse primer (5’- 3’)	Size (bp)
glyceraldehyde 3-phosphate dehydrogenase	*GAPDH*	XR_027767	ACCACTTTGGCATCGTGGAG	GGGCCATCCACAGTCTTCTG	76
peptidylprolyl isomerase A	*PPIA*	NM_178320	CTGGCATCTTGTCCATGGCAAA	CCACAGTCAGCAATGGTGATCTTC	202
mitochondria-localized glutamic acid-rich protein	*MGARP*	NM_001166611	ACGCAGCGATCATGTAACAG	TTCAGGGGCTTCTAAACTGG	115
glycine dehydrogenase (decarboxylating)	*GLDC*	NM_001192951	AAACCAGGGAGCAACACATC	ACAGCCATATTCGCCAAGAG	82
carbohydrate (N-acetylgalactosamine 4–0) sulfotransferase 8	*CHST8*	NM_001145992	CTTGCATGTTCTCGTCCATC	TTTATTCCTGGTGCCTGGTG	106
glutathione peroxidase 3 (plasma)	*GPX3*	NM_174077	CTAGCCACCCTCAAGTATGTTCG	TCACATCGCCTTTCTCAAACAGT	76
follicle stimulating hormone receptor	*FSHR*	NM_174061	GACCCTGATGCCTTCCAGA	TGGCAAGTGCTTAATACCTGTGTT	74
fibulin 5	*FBLN5*	NM_001014946	TGCAACTGAGAATCCCTGTG	GCATTCGTCCATATCACTGC	121
osteoglycin	*OGN*	NM_173946	TGCAAGGCTAATGACACCAG	GATGTTTTCCCAGGATGACG	85
receptor (G protein-coupled) activity modifying protein	*RAMP2*	NM_001098860	CCAAGTCAGAAGGGAAAACG	TAATCAGGGCCCAATCACAC	118
cytochrome P450, family 17, subfamily A, polypeptide 1	*CYP17A1*	NM_174304	ACCATCAGAGAAGTGCTCCGAA	CCACAACGTCTGTGCCTTTGT	115

## Results

### General comparisons

**Fig 1:**
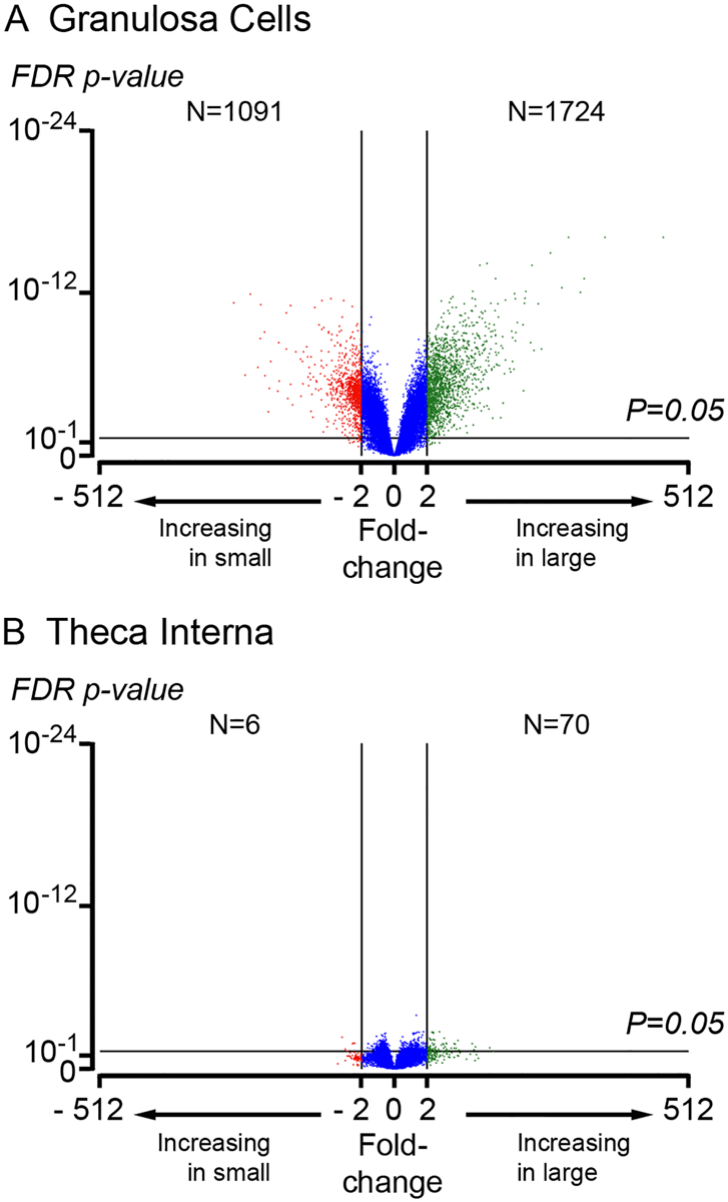
Volcano plots of gene expression in small versus large antral follicles for (A) granulosa cells and (B) theca interna. The X-axis represents the fold-change between small and large follicles and the Y-axis represents the FDR *P* value for statistical significance for differences in gene expression across the arrays.

**Fig 2:**
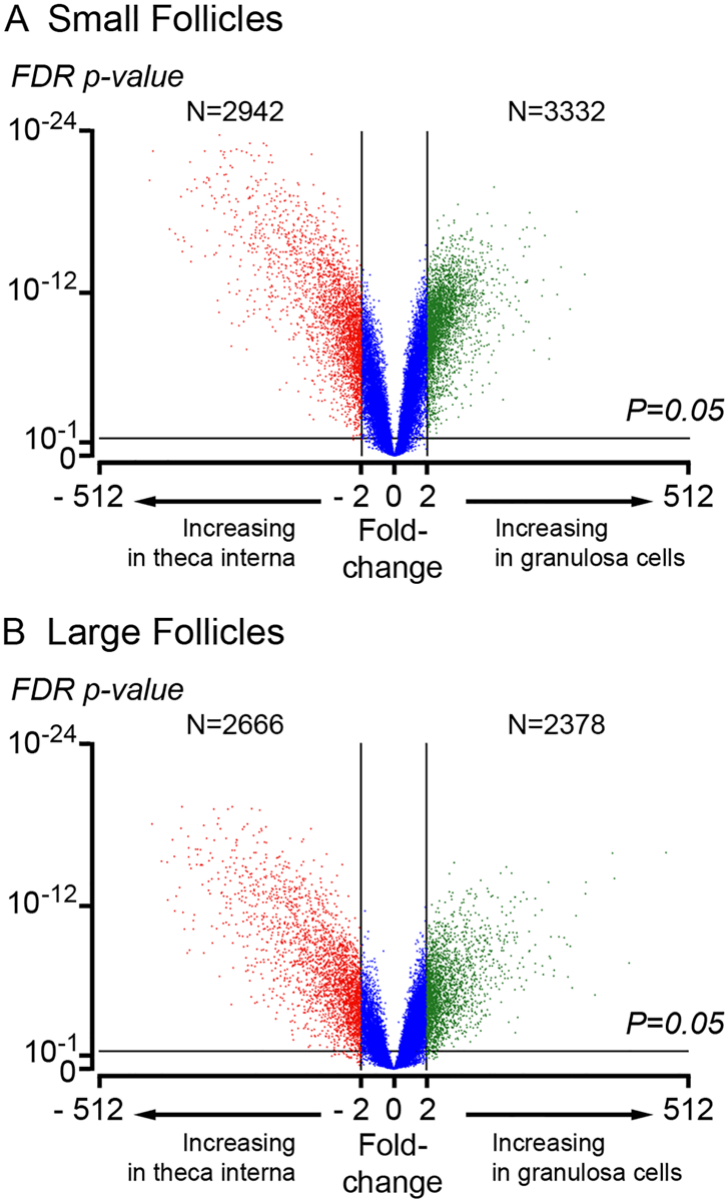
Volcano plots of gene expression in granulosa cells versus theca interna from (A) small and (B) large antral follicles. The X-axis represents the fold-change between small and large follicles and the Y-axis represents the FDR *P* value for statistical significance for differences in gene expression across the arrays.

Volcano plots (log fold change in normalized signal intensity versus statistical significance *P* value) of small versus large follicles for granulosa cells and theca interna are shown in Fig. 1A and B, respectively. Many genes were differentially expressed in granulosa cells between small and large follicles, whilst only relatively few were changed by follicle size in the theca interna (Fig. 1). Volcano plots of granulosa cells versus theca interna from small and large antral follicles are shown in Fig. 2A and B, respectively. Many genes were differentially expressed between granulosa cells and theca interna at both follicle sizes (Fig. 2).

### Differentially expressed genes

Comparisons of array data between theca interna and granulosa cells from small and large healthy follicles were conducted and the fifty most differentially-expressed genes are listed in Tables 2 to 5. The complete list and full gene names and mean intensities on arrays are presented in S1 and S2 Tables. A number of genes were differentially expressed in granulosa cells at both sizes of follicles (Tables 2 and 3; S1 and S2 Tables) and included known genes such as *CYP19A1*, *FSHR* and *NR5A2*. Another 11 genes were common to both lists for the small and large follicles (*LOC404103*, *MGARP*, *GLDC*, *CHST8*, *CSN2*, *GPX3*, *SLC35G1*, *CA8*, *CLGN*, *FAM78A*, *SLC16A3*; Tables 2 and 3). As previously reported [16] theca interna is more uniform from small to large follicles. We found 30 genes (*MGP*, *DCN*, *ASPN*, *ALDH1A1*, *COL1A2*, *FN1*, *COL3A1*, *OGN*, *APOD*, *COL5A2*, *IGF2*, *NID1*, *LHFP*, *ACTA2*, *DUSP12*, *ACTG2*, *SPARCL1*, *FILIP1L*, *EGFLAM*, *ADAMDEC1*, *HPGD*, *COL12A1*, *FBLN5*, *RAMP2*, *COL15A1*, *PLK2*, *COL6A3*, *LOXL1*, *RARRES1*, *FLI1*, *LAMA2*) out of 50 that were common to both sizes of follicles (Tables 4 and 5). Genes up regulated in theca included the stromal matrix genes such as *DCN*, *COL1A2* and *COL3A1*.

**Table 2:** Top 50 genes differentially up regulated in granulosa cells from small follicles.

Gene Symbol	Fold-change	Gene Symbol	Fold-change	Gene Symbol	Fold-change
*PTI*	55.7	*PPARG*	13.8	*CCDC3*	8.9
*LOC404103	47.1	*STRA6*	13.1	*HAUS4*	8.6
*NR5A2	41.0	*GUCA1A*	12.9	*TLL2*	8.3
*IHH*	33.7	*SLC35G1	12.2	*ALG3*	8.2
*UPK1B*	31.0	*LAMC2*	11.6	*EFHD1*	8.2
*CYP19A1	26.5	*TRIM6*	10.5	*FAM78A	8.2
*JAKMIP1*	23.9	*CHRDL1*	10.4	*LOC614107*	8.1
*MGARP	22.7	*MZB1*	10.4	*TCRA*	8.1
*FSHR	19.4	*CA8	10.1	*CRABP2*	7.8
*CDH2*	19.0	*CLGN	9.9	*LOC510844*	7.7
*GLDC	17.4	*CARTPT*	9.9	*SLC16A3	7.7
*CHST8	17.0	*PRR15*	9.8	*SLC10A2*	7.5
*GYLTL1B*	16.4	*NUP210*	9.7	*UGT2B11*	7.5
*CSN2	16.4	*LOC509420*	9.7	*AP3B2*	7.5
*GPX3	16.0	*EPDR1*	9.2	*SVOPL*	7.4
*NOS2*	15.8	*FGFR2*	9.2	*STAC3*	7.4
*CA14*	15.2	*AOAH*	8.9		

The fold change is the ratio of signal intensity of granulosa cell to theca interna from microarray analyses.

*Genes in bold are common to Table 3.

**Table 3:** Top 50 genes differentially up regulated in granulosa cells from large follicles.

Gene Symbol	Fold-change	Gene Symbol	Fold-change	Gene Symbol	Fold-change
*TNFAIP6*	319.3	*SPOCK2*	16.6	*GPT*	11.2
*CYP19A1	147.0	*SLC35G1	15.7	*TNPO1*	11.1
*LRP8*	107.3	*VCAN*	15.3	*NPR3*	10.9
*MGARP	42.3	*TOX*	14.9	*PRR15*	10.9
*NR5A2	40.1	*AP2B1*	14.4	*SLC16A3	10.6
*SLC27A3*	39.1	*INHBA*	13.9	*BTBD7*	10.5
*EFNA5*	37.0	*RRAGD*	13.3	*LOC404103	10.4
*CHST8	30.0	*BEX2*	13.2	*FAM78A	10.4
*CA8	27.6	*GLDC	13.0	*NT5E*	10.4
*CLGN	27.1	*NABP1*	12.9	*SLCO3A1*	10
*APOA2*	26.7	*TFR2*	12.5	*PIK3R1*	9.9
*GPX3	23.8	*IL6R*	12.5	*MTR*	9.9
*LINGO2*	23.0	*SLC39A8*	12.2	*ADAM9*	9.8
*LRRC2*	20.1	*IGSF11*	12.2	*TMEM120A*	9.7
*SUSD4*	20.1	*CSN2	11.9	*LINGO2*	9.6
*FSHR	19.9	*RGN*	11.5	*FST*	9.5
*CITED1*	17.6	*TLL2*	11.4		

The fold change is the ratio of signal intensity of granulosa cell to theca interna from microarray analyses.

*Genes in bold are common to Table 2.

**Table 4:** Top 50 genes differentially up regulated in theca interna from small follicles.

Gene Symbol	Fold-change	Gene Symbol	Fold-change	Gene Symbol	Fold-change
*MGP	*176.6*	*SPARCL1	*52.8*	*COL15A1	*35.7*
*DCN	*102.9*	*ID1*	*52.6*	*PLK2	*35.2*
*ASPN	*96.9*	*FILIP1L	*49.3*	*COL6A3	*33.5*
*ALDH1A1	*91.6*	*EGFLAM	*45.4*	*CDKN1C*	*33.4*
*COL1A2	*88.5*	*ADAMDEC1	*44.7*	*TBX3*	*33.0*
*FN1	*87.3*	*HPGD	*43.7*	*PDGFRA*	*32.9*
*COL3A1	*84.9*	*SDC2*	*43.4*	*XDH*	*32.8*
*OGN	*75.4*	*COL12A1	*42.5*	*LOXL1	*32.1*
*APOD	*74.3*	*STAR*	*40.7*	*RARRES1	*32.0*
*COL5A2	*73.5*	*PLXND1*	*40.5*	*FLI1	*31.9*
*NID2*	*73.4*	*CLEC3B*	*39.9*	*DKK3*	*31.7*
*IGF2	*68.3*	*FBLN5	*39.5*	*LMO7*	*31.6*
*CXCL14*	*65.9*	*DLC1*	*38.6*	*A2M*	*31.3*
*NID1	*56.7*	*TGFBR2*	*37.1*	*SCG2*	*31.3*
*LHFP	*55.5*	*RAMP2	*36.5*	*LAMA2	*31.0*
*ACTA2 */* ***ACTG2***	*55.0*	*IGFBP6*	*36.2*	*LAMB1*	*28.2*
*DUSP12	*53.9*	*C27H8orf4*	*36.0*		

The fold change is the ratio of signal intensity of theca interna to granulosa cells from microarray analyses.

*Genes in bold are common to Table 5.

**Table 5:** Top 50 genes differentially up regulated in theca interna from large follicles.

Gene Symbol	Fold-change	Gene Symbol	Fold-change	Gene Symbol	Fold-change
*COL15A1	*166.6*	*ASPN	*69.4*	*FN1	*42.7*
*ALDH1A1	*136.3*	*COL6A3	*66.6*	*RAMP2	*41.9*
*MGP	*121.3*	*HPGD	*65.2*	*GNG11*	*41.5*
*NID1	*108.5*	*IGF2	*63.6*	*LAMA2	*41.1*
*COL5A2	*104.6*	*COL1A1*	*63.5*	*FLI1	*40.9*
*ACTA2 / ACTG2	*102.9*	*LOC781493*	*60.0*	*CLDN11*	*40.3*
*RARRES1	*99.8*	*ADAMDEC1	*58.7*	*SCARA5*	*39.8*
*SPARCL1	*92.8*	*SDPR*	*57.8*	*FAM101B*	*38.3*
*COL12A1	*90.1*	*CXCL14*	*57.8*	*TAGLN*	*37.2*
*FBLN5	*88.8*	*LHFP	*56.0*	*ITGBL1*	*36.8*
*COL3A1	*86.7*	*CAV1*	*50.5*	*RGS5*	*35.4*
*APOD	*84.7*	*FILIP1L	*49.8*	*ENPP2*	*35.0*
*OGN	*78.0*	*XDH	*49.0*	*ACTN1*	*35.0*
*DUSP12	*77.4*	*DCN	*47.2*	*FBN1*	*34.8*
*SHISA2*	*75.5*	*CD99*	*46.8*	*LOXL1	*34.7*
*EGFLAM	*73.4*	*MMP2*	*45.3*	*A2M*	*34.6*
*COL1A2	*69.5*	*PLK2	*44.7*		

The fold change is the ratio of signal intensity of theca interna to granulosa cells from microarray analyses.

*Genes in bold are common to Table 4.

### Verification of differentially expressed genes by qRT-PCR

**Fig 3:**
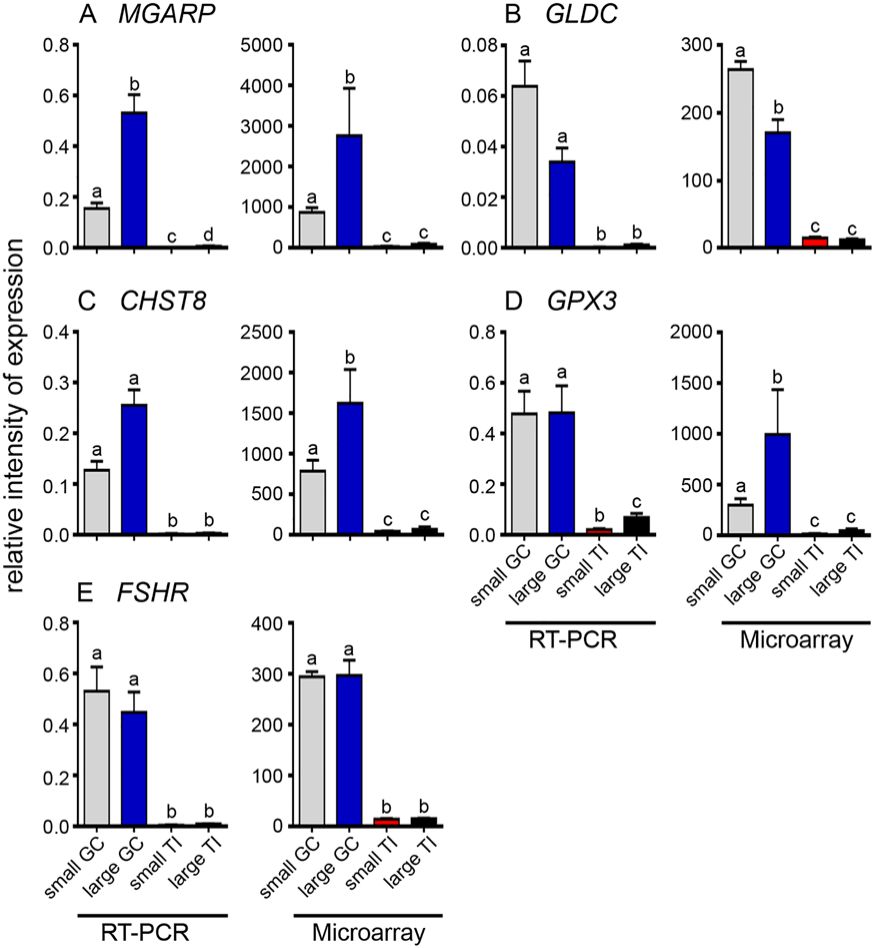
Expression data for up regulated genes in granulosa cells from small and large follicles. The data are shown as the mean ± SEM (n = 10 for small follicle group, n = 4 for large follicle group, GC = granulosa cells, TI = theca interna). qRT-PCR values were determined from the geometric mean of 2^-ΔΔCt^ of the target genes to the cyclophilin A (*PPIA*) and glyceraldehyde phosphate dehydrogenase (*GAPDH*), and the microarray values are signal intensities (normalized but not log transformed). Significantly different results for qRT-PCR were determined by one-way ANOVA with Tukey’s post hoc test. The *P* values for the microarray results were corrected for multiple testing using the FDR. All values which were statistically different from each other are indicated by the different alphabetical symbols in the graphs.

**Fig 4:**
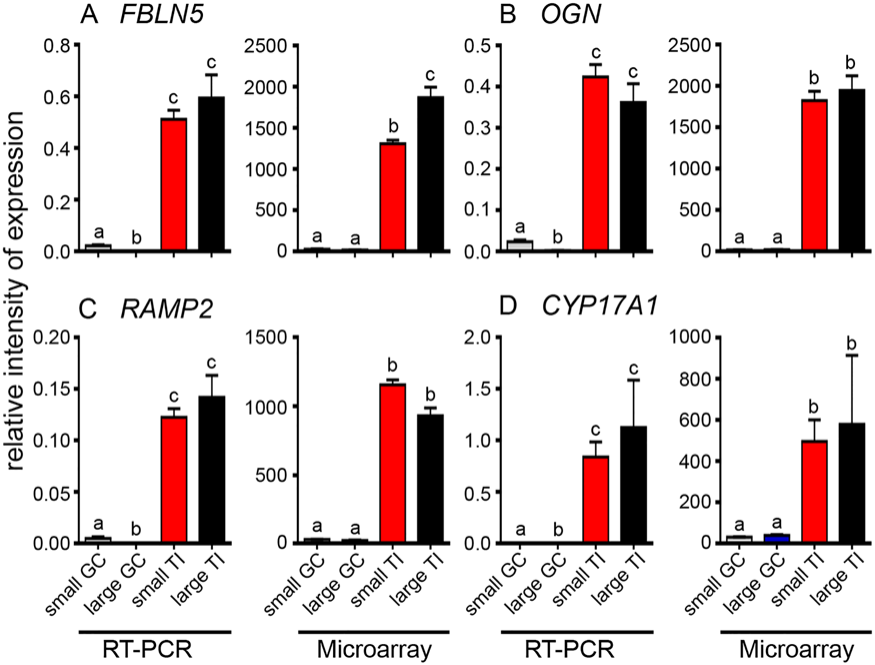
Expression data for up regulated genes in thecal layers from small and large follicles. The data are shown as the mean ± SEM (n = 10 for small follicle group, n = 5 for large follicle group, GC = granulosa cells, TI = theca interna). qRT-PCR values were determined from the geometric mean of 2^-ΔΔCt^ of the target genes to the cyclophilin A (*PPIA*) and glyceraldehyde phosphate dehydrogenase (*GAPDH*), and the microarray values are signal intensities (normalized but not log transformed). Significantly different results for qRT-PCR were determined by one-way ANOVA with Tukey’s post hoc test. The *P* values for the microarray results were corrected for multiple testing using the FDR. All values which are statistically different from each other are indicated by different alphabetical symbols in the graphs.

A number of genes that were differentially up regulated in the granulosa cells (*MGARP*, *GLDC*, *CHST8*, *GPX3* and *FSHR*) or in the theca interna (*FBLN5*, *OGN*, *RAMP2* and *CYP17A1*) for both follicles sizes were selected and their mRNA levels examined in granulosa cells (Fig. 3) and theca interna (Fig. 4) isolated from small and large follicles. In this group of genes two were chosen as well known makers of granulosa cells (*FSHR*) and thecal cells (*CYP17*). The mRNA levels, relative to housekeeping genes *GAPDH* and *PPIA* and the array signal intensities are shown in Figs. 3 and 4. These results show that the microarray and the qRT-PCR data agree remarkably well in relative terms for all the genes examined, thus confirming that microarray analyses have indeed identified genes which are differentially expressed between granulosa cells and theca interna.

## Discussion

Isolation of granulosa and thecal cells by dissection has been used extensively to identify their separate roles in folliculogenesis and to examine the regulation of their growth and development. The degree to which cross contamination can confound the data varies from experiment to experiment and not all authors assess contamination, or need to, particularly if examining genes or molecules which have been well characterized in follicles. However, purity can matter substantially and many authors have assessed the purity of the cell preparations (examples include [8–12]). However, additional markers of these cell types at different stages of follicle growth would be useful to advance research on these cells. To identify such genes we compared granulosa cells and theca interna of two sizes of bovine follicles. These were derived from bovine follicles validated by histology as healthy [15,18]. The purity of the cell types had previously been confirmed [15,18]. RNA microarrays were conducted under similar conditions for the different cell and follicle types and the results from these arrays were validated previously [16,17].

Volcano plots identified that granulosa cells from small and large follicles differed substantially in their transcriptomes, unlike theca interna which was uniform. This initially seems counter intuitive as the mural granulosa cells being an epithelial layer are cellularly homogeneous, whereas the theca interna is a stromal layer with many different cell types. However, the significant changes in the granulosa cells with follicle size, probably reflects their important changing roles in follicular growth at the stages examined. Comparing the transcriptomes of theca interna and granulosa cells at each size, identified that their transcriptomes were very different in both the small and large follicles, with thousands of genes differentially expressed between the two cell types (*P* < 0.05, > 2 fold).

We focused on the 50 most differentially-expressed genes (full list is in the S1 and S2 Tables) between granulosa cells and theca interna within each follicle size group and identified genes common to both sizes of follicles. The genes up regulated in granulosa cells were all more than 7 fold up regulated and those up regulated in the theca interna were all greater than 28 fold up regulated. Of the short lists of up regulated genes for the two follicle sizes only 14 were common to both sizes of follicles for granulosa cells and included genes (*CYP19A1*, *FSHR* and *NR5A2*) known to be differentially up regulated in granulosa cells. The theca interna, which had much more uniform transcriptomes between the two sizes, had 30 genes in common to both short lists. Many of the genes up regulated in the theca interna were collagens and associated genes (*COL1A2*, *COL3A1*, *COL5A2*, *COL6A3*, *COL12A1*, *COL15A1*, *DCN*), other extracellular matrix genes (*LAMA2*, *NID1*, *FBLN5*, *FN1*, *MGP*, *ASPN*, *OGN*, *EGFLAM*) or encoding enzymes that process extracellular matrix (*ADAMDEC1*, *LOXL1*). Whilst these are good candidates for indicating the presence of cells derived from the theca interna in a preparation of granulosa cells some of these genes are also likely to be expressed in the ovarian stroma as the theca interna is a stromal layer [22]. Therefore some of these genes are not likely to be thecal-specific markers but certainly can be used to identify thecal contamination of preparations of granulosa cells.

A number of genes were selected for validation by quantitative RT-PCR for differential expression between granulosa cells and theca interna in both small and large follicles. These genes displayed relatively high array intensity in a particular cell type and are not well characterized in terms of ovarian function. *MGARP*, *GLDC*, *CHST8*, *GPX3* and the known differentially-expressed gene in granulosa cells, *FSHR*, were substantially greater in granulosa cells than theca interna as determined by both RT-PCR and microarray analyses and for both sizes of follicles. *MGARP*, or ovary specific acidic protein (*OSAP*) [23], and *CHST8*, a sulfotransferase, were up regulated in large follicles in the current study and have been shown previously [24]. *GLDC*, which encodes glycine decarboxylase, was increased in small follicles which correspond to a less differentiated phenotype, more similar to pluripotent cells [25]. The extracellular matrix protein genes, *FBLN5* and *OGN*, and *RAMP2*, the adrenomedullin co-receptor, and the known differentially-expressed gene in theca interna, *CYP17A1*, were substantially greater in theca interna than in granulosa cells as determined by both qRT-PCR and microarray analyses and for both sizes of follicles. Thus these results confirm that the microarray analyses were correctly identifying genes differentially expressed in granulosa cells and in theca interna.

Thus in summary, we have identified a substantial number of genes which are differentially expressed between granulosa cells and the theca interna. This information will aid future studies of these cells, identifying the degree of cross contamination and providing a list of genes of interest for further study.

## Supporting Information

S1 Table List of genes > 4-fold differentially-expressed in granulosa cells compared with theca interna in small follicles with FDR *P* < 0.05. Gene name, ID, fold change and mean array intensities are presented.
(PDF)

S2 Table List of genes 4-fold differentially-expressed in granulosa cells compared with theca interna in large follicles with FDR *P* < 0.05. Gene name, ID, fold change and mean array intensities are presented.
(PDF)
