# Supplementary material for: Transcriptome Comparisons Identify New Cell Markers for Theca Interna and Granulosa Cells from Small and Large Antral Ovarian Follicles
Source: PLoS One. 2015 Mar 16;10(3):e0119800. doi: 10.1371/journal.pone.0119800 (PMC4361622; doi:10.1371/journal.pone.0119800)
Supplement: S1 Table — Gene name, ID, fold change and mean array intensities are presented. (PDF) [file pone.0119800.s001.pdf]

Table S1. List of genes > 4-fold differentially expressed in granulosa cells compared with theca interna in small follicles with FDR $P < 0.05$. Gene name, ID, fold change and mean array intensities are presented.

Gene Symbol	Gene Title	RefSeq Transcript ID	Fold-change	Mean $\log_2$ array intensity	
				Granulosa cells	Theca interna
PTI	pancreatic trypsin inhibitor	NM_001001554	55.7	9.3	3.5
LOC404103	spleen trypsin inhibitor	NM_205786	47.1	11.2	5.6
NR5A2	nuclear receptor subfamily 5, group A, member 2	NM_001206816	41.0	10.7	5.3
IHH	Indian hedgehog	NM_001076870	33.7	9.2	4.1
UPK1B	uroplakin 1B	NM_174482	31.0	11.7	6.7
CYP19A1	cytochrome P450, family 19, subfamily A, polypeptide 1	NM_174305	26.5	8.8	4.1
JAKMIP1	janus kinase and microtubule interacting protein 1	NM_001102251	23.9	8.5	3.9
MGARP	chromosome 17 open reading frame, human C4orf49	NM_001166611	22.7	9.8	5.3
FSHR	follicle stimulating hormone receptor	NM_174061	19.4	8.2	3.9
CDH2	cadherin 2, type 1, N-cadherin (neuronal)	NM_001166492	19.0	10.7	6.5
GLDC	glycine dehydrogenase (decarboxylating)	NM_001192951	17.4	8.0	3.9
CHST8	carbohydrate (N-acetylgalactosamine 4-0) sulfotransferase 8	NM_001145992	17.0	9.6	5.5
GYLTL1B	glycosyltransferase-like 1B	NM_001206192	16.4	9.4	5.3
CSN2	casein beta	NM_181008	16.4	10.9	6.8
GPX3	glutathione peroxidase 3 (plasma)	NM_174077	16.0	8.2	4.2
NOS2	nitric oxide synthase 2, inducible	NM_001076799	15.8	11.3	7.3
CA14	carbonic anhydrase XIV	NM_001192205	15.2	8.9	5.0
PPARG	peroxisome proliferator-activated receptor gamma	NM_181024	13.8	9.4	5.6
STRA6	stimulated by retinoic acid gene 6 homolog (mouse)	NM_001075730	13.1	11.6	7.9
GUCA1A	guanylate cyclase activator 1A (retina)	NM_174546	12.9	8.3	4.6
SLC35G1	solute carrier family 35, member G1	NM_001076470	12.2	9.4	5.8
LAMC2	laminin, gamma 2	XM_002694208 /// XM_588297	11.6	8.1	4.5
TRIM6	tripartite motif containing 6	NM_001205189	10.5	7.4	4.0
CHRDL1	chordin-like 1	XM_592894	10.4	9.3	5.9
MZB1	marginal zone B and B1 cell-specific protein	NM_001098930	10.4	8.3	4.9

CA8	carbonic anhydrase VIII	NM_001083690	10.1	7.0	3.7
CLGN	calmegin	NM_001034205	9.9	8.3	5.0
CARTPT	CART prepropeptide	NM_001007820	9.9	9.8	6.5
PRR15	proline rich 15	NM_001205521	9.8	9.2	5.9
NUP210	nucleoporin 210kDa	NM_001103320 /// NM_001191461	9.7	9.0	5.7
LOC509420	chromosome 9 open reading frame 61-like	XM_003582533 /// XM_003586391	9.7	7.6	4.3
EPDR1	ependymin related protein 1 (zebrafish)	NM_001102288	9.2	10.3	7.1
FGFR2	fibroblast growth factor receptor 2	NM_001205310	9.2	7.1	3.9
AOAH	acyloxyacyl hydrolase (neutrophil)	NM_001078096	8.9	8.2	5.1
CCDC3	coiled-coil domain containing 3	NM_001172375	8.9	11.2	8.0
HAUS4	HAUS augmin-like complex, subunit 4	NM_001206190	8.6	7.2	4.1
TLL2	tolloid-like 2	XM_002698408 /// XM_864694	8.3	8.4	5.4
ALG3	asparagine-linked glycosylation 3, alpha-1,3-mannosyltransferase homolog (S. cerevisia	NM_001083511	8.2	7.0	4.0
EFHD1	EF-hand domain family, member D1	NM_001075832	8.2	8.2	5.1
FAM78A	family with sequence similarity 78, member A	NM_001038508	8.2	8.2	5.2
LOC614107	hexokinase 2-like	XM_865470	8.1	7.9	4.8
TCRA	T cell receptor, alpha	NM_001075519 /// NM_001098474	8.1	8.4	5.4
CRABP2	cellular retinoic acid binding protein 2	NM_001008670	7.8	7.4	4.4
LOC510844	zinc finger homeobox protein 3-like	XM_003583394 /// XM_003587229	7.7	7.3	4.4
SLC16A3	solute carrier family 16, member 3 (monocarboxylic acid transporter 4)	NM_001109980	7.7	8.0	5.1
SLC10A2	solute carrier family 10 (sodium/bile acid cotransporter family), member 2	XM_002691982 /// XM_604179	7.5	6.6	3.7
UGT2B11	UDP glucuronosyltransferase 2 family, polypeptide B11	XM_001788093 /// XM_002688319 /// XM_002688320 /// XM_002704592	7.5	6.7	3.8
AP3B2	adaptor-related protein complex 3, beta 2 subunit	NM_001243335	7.5	7.9	5.0

SVOPL	SVOP-like	NM_001192500	7.4	6.5	3.6
STAC3	SH3 and cysteine rich domain 3	NM_001192484	7.4	7.1	4.2
CITED1	Cbp/p300-interacting transactivator, with Glu/Asp-rich carboxy-terminal domain, 1	NM_174518	7.2	8.9	6.1
CHST11	carbohydrate (chondroitin 4) sulfotransferase 11	NM_001192668	7.2	6.6	3.8
SRGN	serglycin	NM_001025326	7.1	13.3	10.5
EFNA5	ephrin-A5	NM_001076432	7.1	6.9	4.1
FST	follistatin	NM_175801	6.9	13.0	10.2
PLCB4	phospholipase C, beta 4	NM_001166510 /// NM_174424	6.9	6.1	3.3
KLHL29	kelch-like 29 (Drosophila)	NM_001164494	6.6	6.4	3.6
LOC505468	cytochrome P450 family 2 subfamily C polypeptide 18-like	XM_002698396 /// XM_002698397 /// XM_002703179 /// XM_003584074 /// XM_003587881 /// XM	6.6	5.9	3.2
TMIGD2	transmembrane and immunoglobulin domain containing 2	XM_002688933 /// XM_602676	6.5	6.6	3.9
NPNT	nephronectin	XM_002688090 /// XM_003586181 /// XM_003586182 /// XM_003586183 /// XM_003586184 /// XM	6.5	11.2	8.5
OFD1Y	oral-facial-digital syndrome 1 Y-linked	XM_002706852	6.5	6.4	3.7
ATP10B	ATPase, class V, type 10B	XM_002689352 /// XM_003582473	6.4	5.8	3.1
INHBA	inhibin, beta A	NM_174363	6.4	11.7	9.0
TBKBP1	TBK1 binding protein 1	XM_001253300 /// XM_002695919	6.4	7.1	4.4
HOPX	HOP homeobox	NM_174097	6.4	8.7	6.0
GALNT13	UDP-N-acetyl-alpha-D-galactosamine:polypeptide N-acetylgalactosaminyltransferase 13 (Ga	NM_001076886	6.4	6.5	3.9
CDK6	cyclin-dependent kinase 6	NM_001192301	6.4	7.7	5.0
FOXO1	forkhead box O1	XM_002691748 ///	6.3	9.4	6.8

		XM_583090			
CAPRIN2	caprin family member 2	XM_002687720 /// XM_871851	6.3	11.2	8.5
PGF	placental growth factor	NM_173950	6.3	8.5	5.8
HS3ST5	heparan sulfate (glucosamine) 3-O-sulfotransferase 5	NM_001076215	6.2	7.1	4.5
PRSS35	protease, serine, 35	NM_001035457	6.2	9.8	7.1
RHO	rhodopsin	NM_001014890	6.2	6.8	4.2
AQP1	aquaporin 1 (Colton blood group)	NM_174702	6.2	7.0	4.4
LOC100847479	serine protease inhibitor Kazal-type 2-like	XM_003582329 /// XM_003586205	6.2	7.1	4.5
GPT	glutamic-pyruvate transaminase (alanine aminotransferase)	NM_001083740	6.2	9.0	6.4
MMP16	matrix metallopeptidase 16 (membrane-inserted)	NM_001192622	6.1	6.5	3.8
ELMO1	engulfment and cell motility 1	NM_001024505 /// NM_001113227	6.1	6.3	3.7
LTF	lactotransferrin	NM_180998	6.1	8.7	6.1
IL17RE	interleukin 17 receptor E	XM_001251390 /// XM_002696936	6.0	8.0	5.4
SUSD4	sushi domain containing 4	NM_001101904	6.0	6.6	4.0
GCLC	glutamate-cysteine ligase, catalytic subunit	NM_001083674	6.0	9.2	6.6
RRAGD	Ras-related GTP binding D	NM_001192828	6.0	7.7	5.1
ITGA2	integrin, alpha 2 (CD49B, alpha 2 subunit of VLA-2 receptor)	NM_001166499	5.9	5.9	3.3
MEX3C	mex-3 homolog C (C. elegans)	XM_002697824 /// XM_607763	5.9	6.7	4.1
TOX2	TOX high mobility group box family member 2	NM_001206688 /// NM_001206689	5.8	8.4	5.9
SLAIN1	SLAIN motif family, member 1	XM_002691900 /// XM_003582874 /// XM_003582875 /// XM_003586721 /// XM_583891	5.8	8.3	5.8
CATHL1 /// LOC100847261	cathelicidin 1 /// cathelicidin-1-like	NM_174825 /// XM_003587646	5.7	5.9	3.4
PLA2G1B	phospholipase A2, group IB (pancreas)	NM_174646	5.7	8.5	6.0

PFKFB3	6-phosphofructo-2-kinase/fructose-2,6-biphosphatase 3	NM_001077837	5.7	7.8	5.3
SMN1	survival of motor neuron 1, telomeric	NM_175701	5.7	7.8	5.3
NABP1	oligonucleotide/oligosaccharide-binding fold containing 2A	NM_001098124	5.6	6.8	4.3
C8H9orf152	chromosome 8 open reading frame, human C9orf152	NM_001206177	5.6	6.0	3.5
CCBL1 /// LOC100335550 /// LOC100852281 /// LOC781863	cysteine conjugate-beta lyase, cytoplasmic /// kynurenine--oxoglutarate transaminase 1-	NM_001077978 /// XM_001790512 /// XM_002706500 /// XM_002706501 /// XM_003585361 /// XM	5.6	9.2	6.7
LINGO2	leucine rich repeat and Ig domain containing 2	XM_002689490 /// XM_590571	5.6	5.6	3.2
TRPA1	transient receptor potential cation channel, subfamily A, member 1	XM_002692726 /// XM_581588	5.6	6.3	3.8
PLEK	pleckstrin	NM_001192496	5.6	7.8	5.3
TTYH1	tweety homolog 1 (Drosophila)	NM_001077015	5.6	7.7	5.3
AGR2	anterior gradient homolog 2 (Xenopus laevis)	NM_001040500	5.6	5.8	3.4
PTGFRN	prostaglandin F2 receptor negative regulator	XM_002686122 /// XM_618406	5.6	7.4	4.9
CPEB1	cytoplasmic polyadenylation element binding protein 1	XM_002696587 /// XM_003583730 /// XM_003587555 /// XM_864691	5.6	7.2	4.7
OASL	2'-5'-oligoadenylate synthetase-like	NM_001081659	5.6	5.9	3.4
PTPRD	protein tyrosine phosphatase, receptor type, D	XM_002689593 /// XM_003582520 /// XM_003582521 /// XM_003582522 /// XM_003582523 /// XM	5.5	7.9	5.4
APOB	---	---	5.5	6.6	4.1
SLC27A3	solute carrier family 27 (fatty acid transporter), member 3	XM_001790632 /// XM_002686020	5.5	7.0	4.6
MUC15	mucin 15, cell surface associated	NM_176631	5.5	6.0	3.6
ATP5SL	ATP5S-like	NM_001272008 /// NM_001272009 ///	5.4	6.7	4.2

		XM_003583433 /// XM_003587269			
MERTK	c-mer proto-oncogene tyrosine kinase	NM_001192024	5.4	7.9	5.4
HMOX1	heme oxygenase (decycling) 1	NM_001014912	5.4	9.9	7.5
RENBP	renin binding protein	NM_001046223	5.3	7.7	5.3
SPEF1	sperm flagellar 1	NM_001034422	5.3	7.6	5.2
ELMOD3	ELMO/CED-12 domain containing 3	NM_001015661	5.3	7.5	5.1
FXYD7	FXYD domain containing ion transport regulator 7	NM_001037627	5.3	7.9	5.5
GPRC5A	G protein-coupled receptor, family C, group 5, member A	NM_001034515	5.2	6.6	4.2
MYO1D	myosin ID	NM_001075838	5.2	6.8	4.4
RASL11B	RAS-like, family 11, member B	NM_001015635	5.2	9.4	7.0
LOC782061	aldo-keto reductase family 1, member C1-like	NM_001166223	5.2	6.1	3.7
EMX2	empty spiracles homeobox 2	NM_001075845	5.2	6.2	3.8
NXPH2	neurexophilin 2	NM_174406	5.2	6.0	3.6
YBX2	Y box binding protein 2	NM_001098126	5.2	6.4	4.1
ALB	albumin	NM_180992	5.2	5.7	3.4
CDH1	cadherin 1, type 1, E-cadherin (epithelial)	NM_001002763	5.2	6.2	3.8
PKP2	plakophilin 2	NM_001083729	5.2	6.1	3.7
GPX2	glutathione peroxidase 2 (gastrointestinal)	NM_001163139	5.2	7.2	4.8
ASB15	ankyrin repeat and SOCS box containing 15	NM_174687	5.1	6.4	4.1
ZP3	zona pellucida glycoprotein 3 (sperm receptor)	NM_173974	5.1	6.9	4.5
TPD52L1	tumor protein D52-like 1	NM_001076033	5.1	6.6	4.3
GUCY2F	guanylate cyclase 2F, retinal	NM_174549	5.1	5.5	3.2
MAP4K1	mitogen-activated protein kinase kinase kinase kinase 1	NM_001075825	5.1	6.7	4.3
TESPA1	thymocyte expressed, positive selection associated 1	XM_002687624 /// XM_584123	5.1	6.0	3.7
RRAD	Ras-related associated with diabetes	NM_001045913	5.1	8.7	6.3
SEL1L3	sel-1 suppressor of lin-12-like 3 (C. elegans)	NM_001206556	5.1	6.4	4.1
BoLA	major histocompatibility complex, class I, A	NM_001114855	5.1	7.5	5.2
SLCO3A1	solute carrier organic anion transporter family, member 3A1	NM_001001134	5.0	7.1	4.8
UXS1	UDP-glucuronate decarboxylase 1	NM_001206064	5.0	7.5	5.1
RBM24	RNA binding motif protein 24	NM_001104985	5.0	7.0	4.7

TMEM156	transmembrane protein 156	NM_001083469	5.0	6.5	4.1
TRAM1	translocation associated membrane protein 1	NM_001040476	5.0	6.6	4.3
UBN2	ubinuclein 2	NM_001193208	5.0	6.1	3.7
RPRM	reprimo, TP53 dependent G2 arrest mediator candidate	NM_001080739	5.0	7.1	4.7
OBSL1	obscurin-like 1	NM_001075491	5.0	7.1	4.7
CCDC159	coiled-coil domain containing 159	XM_001253294 /// XM_002688871	4.9	7.3	5.0
SLC24A1	solute carrier family 24 (sodium/potassium/calcium exchanger), member 1	NM_174655	4.9	6.6	4.3
PRSS22	protease, serine, 22	XM_002697927 /// XM_587533	4.9	6.3	4.0
ROR2	receptor tyrosine kinase-like orphan receptor 2	NM_001105464	4.9	10.0	7.8
FSHB	follicle stimulating hormone, beta polypeptide	NM_174060	4.9	6.1	3.8
ATP13A2	ATPase type 13A2	NM_001192271	4.8	8.0	5.8
MFGE8	milk fat globule-EGF factor 8 protein	NM_176610	4.8	11.9	9.7
RCN3	reticulocalbin 3, EF-hand calcium binding domain	NM_001046260	4.8	6.4	4.2
F2RL2	coagulation factor II (thrombin) receptor-like 2	NM_001038533	4.8	6.7	4.4
STAC	SH3 and cysteine rich domain	NM_001077962	4.8	7.1	4.8
CD72	CD72 molecule	XM_001251357 /// XM_002689678	4.8	6.3	4.1
KIAA1549	KIAA1549 ortholog	NM_001080267	4.8	6.0	3.7
CCNT1	cyclin T1	NM_001001147	4.7	6.4	4.2
CHAC1	ChaC, cation transport regulator homolog 1 (E. coli)	NM_001098882	4.7	7.2	5.0
MOB3B	MOB kinase activator 3B	NM_001046491	4.7	8.2	5.9
C6	complement component 6	NM_001045979	4.7	5.2	3.0
HOOK1	hook homolog 1 (Drosophila)	NM_001206870 /// XM_003582004	4.7	6.4	4.2
LRRC2	leucine rich repeat containing 2	NM_001080311	4.7	7.6	5.3
DBT	dihydrolipoamide branched chain transacylase E2	NM_173905	4.7	6.1	3.8
PLOD2	procollagen-lysine, 2-oxoglutarate 5-dioxygenase 2	NM_001101149	4.7	10.1	7.8
ABP1	amiloride binding protein 1 (amine oxidase (copper-containing))	NM_001034361	4.7	5.5	3.2
TRAM2	translocation associated membrane protein 2	NM_001193150	4.7	6.2	3.9

CMBL	carboxymethylenebutenolidase homolog (Pseudomonas)	NM_001192983	4.7	11.5	9.3
LTA	lymphotoxin alpha (TNF superfamily, member 1)	NM_001013401	4.7	5.9	3.6
PLP1	proteolipid protein 1	NM_174149	4.6	5.9	3.7
PKM	pyruvate kinase, muscle	NM_001205727	4.6	9.4	7.2
PDE6A	phosphodiesterase 6A, cGMP-specific, rod, alpha	NM_001001526	4.6	5.6	3.4
HPSE	heparanase	NM_174082	4.6	6.4	4.2
FAM184A	family with sequence similarity 184, member A	XM_002690079 /// XM_002705132 /// XM_614661	4.6	7.2	5.0
GATM	glycine amidinotransferase (L-arginine:glycine amidinotransferase)	NM_001045878	4.6	10.0	7.9
PDP2	pyruvate dehyrogenase phosphatase catalytic subunit 2	XM_002694806 /// XM_003584879	4.5	8.9	6.7
CD33	CD33 antigen-like	NM_001191347	4.5	6.1	3.9
SKAP1	src kinase associated phosphoprotein 1	NM_001192110 /// XM_002695944 /// XM_003583599	4.5	5.5	3.3
SLC22A23	solute carrier family 22, member 23	NM_001076341	4.5	6.3	4.1
LNP1	leukemia NUP98 fusion partner 1	NM_001205535	4.5	6.4	4.3
NSUN7	NOP2/Sun domain family, member 7	XM_002688230 /// XM_612659	4.5	5.4	3.2
TNPO1	transportin 1	NM_001076540	4.5	9.1	6.9
PRPF40A	PRP40 pre-mRNA processing factor 40 homolog A (S. cerevisiae)	XM_002685353 /// XM_003584917 /// XM_003584918 /// XM_003585746	4.5	6.7	4.5
CGN	cingulin	NM_001192786	4.5	8.6	6.4
SCD	stearoyl-CoA desaturase (delta-9-desaturase)	NM_173959 /// XM_002698490	4.5	11.5	9.4
TARP	TCR gamma alternate reading frame protein	XM_002706437 /// XM_003585963	4.5	8.1	5.9
BMP15	bone morphogenetic protein 15	NM_001031752	4.5	6.2	4.0
CCL28	chemokine (C-C motif) ligand 28	NM_001101163	4.4	6.2	4.1
ZNF385A	zinc finger protein 385A	NM_001206001	4.4	6.3	4.1

SLC38A2	solute carrier family 38, member 2	NM_001082424	4.4	6.8	4.7
TMEM138	transmembrane protein 138	NM_001098967	4.4	7.0	4.8
TGIF1	TGFB-induced factor homeobox 1	NM_001128499	4.4	8.8	6.7
HRG	histidine-rich glycoprotein	NM_173919	4.4	5.8	3.7
UCHL1	ubiquitin carboxyl-terminal esterase L1 (ubiquitin thiolesterase)	NM_001046172	4.4	11.2	9.1
IL18R1	interleukin 18 receptor 1	XM_002691182 /// XM_590497	4.4	5.3	3.2
ELK1	ELK1, member of ETS oncogene family	NM_001191236	4.4	8.1	6.0
TCAIM	T cell activation inhibitor, mitochondrial	NM_001102016	4.4	7.3	5.1
TMEM120A	transmembrane protein 120A	NM_001079600	4.4	9.8	7.7
HEATR1	HEAT repeat containing 1	NM_001076448 /// XM_583512 /// XM_585511	4.3	6.0	3.9
TMEM216	transmembrane protein 216	NM_001166563	4.3	6.6	4.5
OPRM1	opioid receptor, mu 1	NM_174408	4.3	5.6	3.5
CYLC1	cylicin, basic protein of sperm head cytoskeleton 1	NM_174302	4.3	5.5	3.3
SLC27A2	solute carrier family 27 (fatty acid transporter), member 2	NM_001192863	4.3	5.2	3.1
TBC1D8	TBC1 domain family, member 8 (with GRAM domain)	NM_001206142	4.3	9.5	7.4
FMO2	flavin containing monooxygenase 2 (non-functional)	NM_001075162 /// NM_001163274	4.3	5.6	3.5
MPDZ	multiple PDZ domain protein	NM_001192891	4.3	5.8	3.7
SLC38A1	solute carrier family 38, member 1	XM_001790621 /// XM_002687321	4.3	5.3	3.2
KCNE1	potassium voltage-gated channel, Isk-related family, member 1	NM_001077977	4.3	5.9	3.8
LOC785870 /// PDYN	PDYN protein-like /// prodynorphin	NM_174139 /// XM_001253283	4.3	5.5	3.4
TRGV7-1	T cell receptor gamma variable 7-1	---	4.3	5.4	3.3
DNAJC6	DnaJ (Hsp40) homolog, subfamily C, member 6	NM_174836	4.2	5.7	3.6
USP28	ubiquitin specific peptidase 28	NM_001192998	4.2	5.5	3.5
UAP1L1	UDP-N-acteylglucosamine pyrophosphorylase 1-like 1	XM_002691711 /// XM_003585073	4.2	7.6	5.5

BTN3A3	butyrophilin, subfamily 3, member A3	NM_001034207	4.2	5.9	3.8
HYDIN	HYDIN, axonemal central pair apparatus protein	XM_002694849 /// XM_002701816	4.2	6.2	4.1
STXBP2	syntaxin binding protein 2	NM_001046208	4.2	8.8	6.7
VSX1	visual system homeobox 1	NM_174767	4.2	5.7	3.6
KLC2	kinesin light chain 2	NM_001075768	4.2	6.9	4.8
IFIT2	interferon-induced protein with tetratricopeptide repeats 2	XM_001787823 /// XM_002698356	4.2	6.5	4.4
TPP1	tripeptidyl peptidase I	NM_001075718	4.2	7.2	5.2
PNLIPRP2	pancreatic lipase-related protein 2	NM_001105355	4.2	6.2	4.1
MEST	mesoderm specific transcript homolog (mouse)	NM_001083368	4.2	11.3	9.2
PAPSS2	3'-phosphoadenosine 5'-phosphosulfate synthase 2	NM_001076075	4.2	10.4	8.3
NARS	asparaginyl-tRNA synthetase	NM_001046572	4.2	5.1	3.1
CDH26	cadherin 26	XM_002692262 /// XM_869285	4.2	7.1	5.1
LAT	linker for activation of T cells	NM_001104978	4.2	7.1	5.1
CLDN6	claudin 6	NM_001205697	4.2	6.3	4.3
ENC1	ectodermal-neural cortex 1 (with BTB-like domain)	NM_001078067	4.2	6.5	4.4
MMD	monocyte to macrophage differentiation-associated	NM_001075601	4.1	7.4	5.3
CTDSPL2	CTD (carboxy-terminal domain, RNA polymerase II, polypeptide A) small phosphatase like	NM_001191405	4.1	5.5	3.4
ANGPT2	angiopoietin 2	NM_001098855	4.1	9.4	7.3
HTR2A	5-hydroxytryptamine (serotonin) receptor 2A	NM_001001157	4.1	5.8	3.7
SORT1	sortilin 1	XM_002686189 /// XM_002703890 /// XM_003581961 /// XM_003585853	4.1	8.1	6.1
IL21	interleukin 21	NM_198832	4.1	5.2	3.2
SLC5A9	solute carrier family 5 (sodium/glucose cotransporter), member 9	NM_001205936	4.1	6.0	4.0
TYRP1	tyrosinase-related protein 1	NM_174480	4.1	5.2	3.1
ANTXR2	anthrax toxin receptor 2	NM_001076826	4.1	7.3	5.3
ID2	inhibitor of DNA binding 2, dominant negative helix-loop-	NM_001034231	4.1	6.6	4.6

	helix protein				
NCOA7	nuclear receptor coactivator 7	NM_001102254 /// XM_003582598 /// XM_003586458	4.1	8.0	6.0
APOBEC4	apolipoprotein B mRNA editing enzyme, catalytic polypeptide-like 4 (putative)	XM_002694210 /// XM_003584869	4.1	5.5	3.5
FGF11	fibroblast growth factor 11	NM_001192939	4.1	5.6	3.6
MDC1	mediator of DNA-damage checkpoint 1	XM_002697355 /// XM_588451	4.1	7.2	5.2
CADPS	Ca++-dependent secretion activator	NM_001076020	4.1	5.3	3.3
TMOD1	tropomodulin 1	NM_001079640	4.1	6.8	4.8
CHN2	chimerin (chimaerin) 2	NM_001045963	4.1	7.0	5.0
SLC7A1	solute carrier family 7 (cationic amino acid transporter, y+ system), member 1	NM_001135792	4.1	9.0	7.0
BTD	biotinidase	NM_001102206	4.1	6.8	4.8
COL10A1	collagen, type X, alpha 1	NM_174634	4.1	6.1	4.1
IGSF1	immunoglobulin superfamily, member 1	NM_001105048	4.0	6.6	4.6
C16H1orf170	uncharacterized protein C1orf170 homolog	NM_001098048	4.0	6.6	4.6
NUDT10	nudix (nucleoside diphosphate linked moiety X)-type motif 10	NM_001035488	4.0	7.0	5.0
ISCU	iron-sulfur cluster scaffold homolog (E. coli)	NM_001075683	4.0	5.6	3.6
VWA8	von Willebrand factor A domain containing 8	XM_002691823 /// XM_602479	4.0	6.6	4.6
CTNND2	catenin (cadherin-associated protein), delta 2 (neural plakophilin-related arm-repeat p	XM_002696428 /// XM_601963	4.0	5.7	3.7
HEG1	HEG homolog 1 (zebrafish)	XM_002684814 /// XM_589074	4.0	9.2	7.2
SLCO2B1	solute carrier organic anion transporter family, member 2B1	NM_174843	-4.0	5.0	7.0
MSN	moesin	NM_001046477	-4.0	7.9	9.9
VAMP5	vesicle-associated membrane protein 5 (myobrevin)	NM_001046476	-4.0	7.0	9.0
CPEB2	cytoplasmic polyadenylation element binding protein 2	XM_002688462 /// XM_002704640	-4.0	6.9	8.9
FRY	furry homolog (Drosophila)	NM_001205616	-4.0	5.4	7.4

WTIP	Wilms tumor 1 interacting protein	NM_001205562	-4.0	5.8	7.8
TESC	tescalcin	XM_002694586 /// XM_002701712	-4.0	4.6	6.6
DUSP10	dual specificity phosphatase 10	NM_001034725	-4.0	3.8	5.8
SCLY	selenocysteine lyase	NM_001083804	-4.0	5.0	7.0
COL4A6	collagen, type IV, alpha 6	XM_002699886 /// XM_601826	-4.0	5.4	7.4
RFTN2	raftlin family member 2	NM_001097986	-4.0	4.6	6.6
ITPR1	inositol 1,4,5-trisphosphate receptor, type 1	NM_174841	-4.0	7.4	9.5
ETS2	v-ets erythroblastosis virus E26 oncogene homolog 2 (avian)	NM_001080214	-4.0	4.9	6.9
ACSL5 /// LOC100851804	acyl-CoA synthetase long-chain family member 5 /// long-chain-fatty-acid--CoA ligase 5-	NM_001075650 /// XM_003585076	-4.1	6.0	8.0
FOXP2	forkhead box P2	NM_001205569	-4.1	5.0	7.0
CYBA	cytochrome b-245, alpha polypeptide	NM_174034	-4.1	5.8	7.8
MIA3	melanoma inhibitory activity family, member 3	NM_001166571	-4.1	6.5	8.5
TCF7L1	transcription factor 7-like 1 (T-cell specific, HMG-box)	XM_002691408 /// XM_593301	-4.1	4.7	6.7
SWAP70	SWAP switching B-cell complex 70kDa subunit	NM_001080297	-4.1	5.5	7.6
PPP2R2B	protein phosphatase 2, regulatory subunit B, beta	NM_001014879 /// NM_001272085 /// NR_073586	-4.1	4.6	6.6
TOPORS	topoisomerase I binding, arginine/serine-rich, E3 ubiquitin protein ligase	NM_001192578	-4.1	4.4	6.4
TSHZ1	teashirt zinc finger homeobox 1	XM_002697657 /// XM_594245	-4.1	6.3	8.3
MRC1	mannose receptor, C type 1	XM_003582920 /// XM_003582925 /// XM_003586772	-4.1	4.2	6.2
CD81	CD81 molecule	NM_001035099	-4.1	9.1	11.2
S100B	S100 calcium binding protein B	NM_001034555	-4.1	6.3	8.4
SMTN	smoothelin	NM_001076879	-4.1	5.6	7.6
B2M /// B2M	beta-2-microglobulin /// beta-2-microglobulin-like	NM_173893 /// XM_001251107 /// XM_002691119	-4.1	9.6	11.6

MICALL2	MICAL-like 2	NM_001098932	-4.2	4.2	6.3
CORO1C	coronin, actin binding protein, 1C	NM_001081590	-4.2	7.4	9.5
LCAT	lecithin-cholesterol acyltransferase	NM_001046069	-4.2	5.9	8.0
CYYR1	cysteine/tyrosine-rich 1	NM_001078105	-4.2	4.3	6.3
SH3RF1	SH3 domain containing ring finger 1	NM_001098065 /// XM_003586353	-4.2	5.9	8.0
TAX1BP3	Tax1 (human T-cell leukemia virus type I) binding protein 3	NM_001034474	-4.2	7.8	9.8
C16H1orf115	chromosome 16 open reading frame, human C1orf115	NM_001205383	-4.2	7.3	9.4
SNRK	SNF related kinase	XM_002696930 /// XM_002702734	-4.2	7.2	9.3
PDLIM1	PDZ and LIM domain 1	NM_001035455	-4.2	7.8	9.9
IMPA1	inositol(myo)-1(or 4)-monophosphatase 1	NM_174361	-4.2	6.0	8.1
ERCC6L2	excision repair cross-complementing rodent repair deficiency, complementation group 6-l	NM_001082606 /// NM_001103310 /// XM_003582565 /// XM_003586422	-4.2	4.5	6.5
ADA	adenosine deaminase	NM_173887	-4.2	5.7	7.7
MAP7D1	MAP7 domain containing 1	XM_002686551 /// XM_589552	-4.3	6.8	8.9
IGJ	immunoglobulin J polypeptide, linker protein for immunoglobulin alpha and mu polypeptid	NM_175773	-4.3	5.6	7.7
ALPL	alkaline phosphatase, liver/bone/kidney	NM_176858	-4.3	5.4	7.5
RAB27A	RAB27A, member RAS oncogene family	NM_001101270	-4.3	4.9	7.0
ANKRD29	ankyrin repeat domain 29	NM_001102104	-4.3	5.5	7.6
PTPLAD2	protein tyrosine phosphatase-like A domain containing 2	NM_001076522	-4.3	5.5	7.6
PDGFRL	platelet-derived growth factor receptor-like	NM_001035301	-4.3	5.0	7.1
CEBPB	CCAAT/enhancer binding protein (C/EBP), beta	NM_176788	-4.3	3.7	5.8
SLCO2A1	solute carrier organic anion transporter family, member 2A1	NM_174829	-4.3	4.8	6.9
LOC781036	dnaJ homolog subfamily C member 1-like	XM_003582915 /// XM_003582917 /// XM_003586766	-4.3	4.7	6.8
CAMK2N1	calcium/calmodulin-dependent protein kinase II inhibitor 1	NM_001114520	-4.3	4.9	7.0
FDX1	ferredoxin 1	NM_181011	-4.3	8.6	10.7

CALML4	calmodulin-like 4	NM_001034671	-4.3	5.3	7.4
NEDD4L	neural precursor cell expressed, developmentally down-regulated 4-like	XM_002697776 /// XM_587080	-4.3	5.7	7.8
PCSK6	proprotein convertase subtilisin/kexin type 6	XM_002696654 /// XM_603014	-4.3	5.0	7.1
MEIS2	Meis homeobox 2	NM_001076175 /// NM_001191269	-4.3	6.1	8.3
MRVI1	murine retrovirus integration site 1 homolog	NM_174392 /// NM_194465	-4.3	5.1	7.2
EPS8	epidermal growth factor receptor pathway substrate 8	NM_001076102 /// XM_003582254 /// XM_003586110	-4.4	4.4	6.5
CD1D	CD1D antigen, d polypeptide	NM_001205381	-4.4	4.3	6.4
IGIP	IgA regulatory protein	NM_205771	-4.4	4.7	6.9
KAT2B	K(lysine) acetyltransferase 2B	XM_002684716 /// XM_613744	-4.4	7.6	9.7
MMRN1	multimerin 1	NM_001102500	-4.4	3.9	6.0
GRK5	G protein-coupled receptor kinase 5	NM_174331	-4.4	4.9	7.0
FMNL3	formin-like 3	NM_001191506	-4.4	5.2	7.3
CCL16	chemokine (C-C motif) ligand 16	XM_002695627 /// XM_868834	-4.4	3.4	5.5
GLUL	glutamate-ammonia ligase	NM_001040474	-4.4	9.3	11.4
CXCR4	chemokine (C-X-C motif) receptor 4	NM_174301	-4.4	5.0	7.1
RBPMS	RNA binding protein with multiple splicing	NM_001046535	-4.4	4.9	7.0
DSTN	destrin (actin depolymerizing factor)	NM_001015586	-4.4	7.7	9.8
RAD52	RAD52 homolog (S. cerevisiae)	NM_001024525	-4.4	4.2	6.3
VAT1	vesicle amine transport protein 1 homolog (T. californica)	NM_001192265	-4.4	7.1	9.3
NOV	nephroblastoma overexpressed gene	NM_001102382	-4.4	5.8	7.9
SDE2	SDE2 telomere maintenance homolog (S. pombe)	NM_001099065	-4.4	4.5	6.7
SCARF1	scavenger receptor class F, member 1	XM_002695682 /// XM_002702111	-4.4	4.7	6.8
CKB /// LOC100851864	creatine kinase, brain /// creatine kinase B-type-like	NM_001015613 /// XM_003583773	-4.4	5.8	7.9

PPP1R16B	protein phosphatase 1, regulatory subunit 16B	NM_174824	-4.4	4.7	6.9
EPB41L4A	erythrocyte membrane protein band 4.1 like 4A	NM_001105384	-4.5	5.6	7.8
TAP1	transporter 1, ATP-binding cassette, sub-family B (MDR/TAP)	NM_001098058	-4.5	6.2	8.4
NRP2	neuropilin 2	NM_001193237	-4.5	4.6	6.8
IFITM3 /// IFITM3 /// LOC786073	interferon induced transmembrane protein 3 /// interferon induced transmembrane protein	NM_001078141 /// NM_181867 /// XM_001253013 /// XM_003586693	-4.5	9.6	11.7
VAV3	vav 3 guanine nucleotide exchange factor	XM_002686162 /// XM_615898	-4.5	4.5	6.6
TFF2	trefoil factor 2	NM_001083521	-4.5	5.4	7.6
LIMCH1	LIM and calponin homology domains 1	NM_001191521	-4.5	4.4	6.6
FLNB	filamin B, beta	NM_001191460	-4.5	6.7	8.8
FAM109B	family with sequence similarity 109, member B	NM_001045936	-4.5	5.4	7.6
EHBP1L1	EH domain binding protein 1-like 1	NM_001191243	-4.5	3.5	5.6
PKIG	protein kinase (cAMP-dependent, catalytic) inhibitor gamma	NM_205812	-4.5	5.6	7.7
LOC100849300 /// MAP2K6	dual specificity mitogen-activated protein kinase kinase 6-like /// mitogen-activated p	NM_001034045 /// XM_003585085	-4.5	5.3	7.5
LOC100337091	1-phosphatidylinositol-4,5-bisphosphate phosphodiesterase gamma-2-like	XM_003583364 /// XM_003585201 /// XM_003587203	-4.5	4.6	6.7
HOXD9	homeobox D9	XM_002685288 /// XM_002703599	-4.5	6.2	8.4
LOC504548 /// UBD	ubiquitin D-like /// ubiquitin D	NM_001206473 /// XM_002697377 /// XM_580689	-4.5	4.7	6.8
JARID2	jumonji, AT rich interactive domain 2	NM_001192656 /// XM_002697579 /// XM_003583926	-4.5	6.8	9.0
NXN	nucleoredoxin	NM_001102136	-4.5	6.0	8.2
SULT1A1	sulfotransferase family, cytosolic, 1A, phenol-preferring, member 1	NM_177521	-4.5	6.2	8.4
WNT2B	wingless-type MMTV integration site family, member 2B	NM_001099363	-4.5	6.2	8.4

MECOM	MDS1 and EVI1 complex locus	XM_002684943 /// XM_003581739 /// XM_003581740 /// XM_003581741 /// XM_003581742 /// XM	-4.6	4.2	6.4
ARAP3	ArfGAP with RhoGAP domain, ankyrin repeat and PH domain 3	XM_003582447 /// XM_003586307	-4.6	4.9	7.1
LOC782991 /// SIRPA	tyrosine-protein phosphatase non-receptor type substrate 1-like /// signal-regulatory p	NM_175788 /// XR_083446	-4.6	4.3	6.5
FREM1	FRAS1 related extracellular matrix 1	NM_001192995	-4.6	4.2	6.4
CYP11A1	cytochrome P450, family 11, subfamily A, polypeptide 1	NM_176644 /// XM_003587562	-4.6	9.3	11.5
GLTSCR2	glioma tumor suppressor candidate region gene 2	NM_001038507	-4.6	6.3	8.5
CXHXorf57	chromosome X open reading frame, human CXorf57	XM_001789923 /// XM_002699835 /// XM_003584302 /// XM_003588125	-4.6	4.7	6.9
ARMCX3	armadillo repeat containing, X-linked 3	NM_001192453	-4.6	6.0	8.2
RBM25	RNA binding motif protein 25	XM_002690998 /// XM_879644	-4.6	3.5	5.7
LOC785805	collagen alpha-5(VI) chain-like	XM_003581780 /// XM_003585711	-4.6	3.6	5.8
SH3KBP1	SH3-domain kinase binding protein 1	NM_001128500	-4.7	4.9	7.1
LASP1 /// LOC787753	LIM and SH3 protein 1 /// LIM and SH3 domain protein 1-like	NM_001035394 /// XM_001255031	-4.7	8.3	10.5
NUCB2	nucleobindin 2	NM_001075381	-4.7	5.9	8.1
COL4A5	collagen, type IV, alpha 5	XM_001790133 /// XM_001790134 /// XM_002699860 /// XM_002699862	-4.7	5.9	8.1
KLF6	Kruppel-like factor 6	NM_001035271	-4.7	4.7	6.9
SULT1B1	sulfotransferase family, cytosolic, 1B, member 1	NM_001075823	-4.7	4.7	7.0
PLEKHO1	pleckstrin homology domain containing, family O member 1	NM_001083797	-4.7	5.8	8.0
PRKCB	protein kinase C, beta	NM_174587	-4.7	3.9	6.1

SEMA5A	sema domain, seven thrombospondin repeats (type 1 and type 1-like), transmembrane domai	XM_002696441 /// XM_583112	-4.7	5.2	7.5
RGS19	regulator of G-protein signaling 19	NM_001076915	-4.7	4.7	7.0
LLGL2	lethal giant larvae homolog 2 (Drosophila)	NM_001099098	-4.7	3.3	5.5
LOC100848191	latent-transforming growth factor beta-binding protein 4-like	XM_003587266 /// XR_138945	-4.7	4.8	7.0
DBNDD2	dysbindin (dystrobrevin binding protein 1) domain containing 2	NM_001130748	-4.7	4.5	6.7
NFKBIA	nuclear factor of kappa light polypeptide gene enhancer in B-cells inhibitor, alpha	NM_001045868	-4.7	6.3	8.5
KIAA0408	KIAA0408 ortholog	XM_001789720 /// XM_002690052	-4.7	4.4	6.6
ROCK1	Rho-associated, coiled-coil containing protein kinase 1	NM_001191227 /// XM_002697789 /// XM_003583969	-4.7	5.5	7.7
FKBP11	FK506 binding protein 11, 19 kDa	NM_001045932	-4.7	7.6	9.8
LARP6	La ribonucleoprotein domain family, member 6	NM_001099205	-4.7	4.7	6.9
EFEMP1	EGF containing fibulin-like extracellular matrix protein 1	NM_001081717	-4.8	4.1	6.3
KLHL28	kelch-like 28 (Drosophila)	NM_001099030	-4.8	6.4	8.6
SRPX	sushi-repeat containing protein, X-linked	NM_001040489	-4.8	6.1	8.3
RIMS1	regulating synaptic membrane exocytosis 1	XM_002690015 /// XM_003582588 /// XM_003582589 /// XM_003586450 /// XM_003586451 /// XM	-4.8	3.8	6.1
NNAT	neuronatin	NM_001201324 /// NM_178323	-4.8	3.9	6.2
LOC100851547 /// NMI	N-myc-interactor-like /// N-myc (and STAT) interactor	NM_001035098 /// XM_003581830	-4.8	5.9	8.2
SAT1	spermidine/spermine N1-acetyltransferase 1	NM_001034333	-4.8	8.4	10.7
DUSP1	dual specificity phosphatase 1	NM_001046452	-4.8	7.4	9.7
FBLN2	fibulin 2	XM_002697131 /// XM_589271	-4.8	4.3	6.5
RGS16	regulator of G-protein signaling 16	NM_174450	-4.8	5.3	7.6

Sep-06	septin 6	NM_001035430	-4.8	3.9	6.2
NTRK2	neurotrophic tyrosine kinase, receptor, type 2	NM_001075225	-4.8	4.0	6.3
HLF	hepatic leukemia factor	NM_001192437	-4.8	5.1	7.4
HDAC7	histone deacetylase 7	NM_001193141	-4.8	6.4	8.7
ODZ2	odz, odd Oz/ten-m homolog 2 (Drosophila)	XM_001788056 /// XM_002689363 /// XM_003582481 /// XM_003582482 /// XM_003582483 /// XM	-4.8	4.2	6.5
BACE2	beta-site APP-cleaving enzyme 2	NM_001206062	-4.8	5.7	7.9
FAM171A1	family with sequence similarity 171, member A1	NM_001102180	-4.8	6.1	8.4
MARCKS	myristoylated alanine-rich protein kinase C substrate	NM_001076276	-4.8	8.1	10.3
ACSL1	acyl-CoA synthetase long-chain family member 1	NM_001076085	-4.8	6.8	9.1
EML1	echinoderm microtubule associated protein like 1	XM_002696768 /// XM_590509	-4.8	5.1	7.4
APOLD1	apolipoprotein L domain containing 1	NM_001101180	-4.8	5.0	7.3
ZNF462	zinc finger protein 462	NM_001205811	-4.9	6.1	8.4
NFKBIZ	nuclear factor of kappa light polypeptide gene enhancer in B-cells inhibitor, zeta	NM_174726	-4.9	5.2	7.5
TIE1	tyrosine kinase with immunoglobulin-like and EGF-like domains 1	NM_173965	-4.9	4.3	6.6
ANKH	ankylosis, progressive homolog (mouse)	NM_001109793	-4.9	5.5	7.8
PFKM	phosphofructokinase, muscle	NM_001075268	-4.9	6.4	8.6
LOC100851475 /// LOC100852174 /// SRPX2	sushi repeat-containing protein SRPX2-like /// sushi repeat-containing protein SRPX2-li	NM_001014926 /// XM_003584298 /// XM_003585524	-4.9	4.1	6.4
VCL	vinculin	NM_001078093 /// NM_001191370	-4.9	4.8	7.1
IGF2R	insulin-like growth factor 2 receptor	NM_174352	-4.9	6.5	8.8
PPP1R1A	protein phosphatase 1, regulatory (inhibitor) subunit 1A	NM_001130760	-4.9	5.6	7.9
KIAA1217	KIAA1217 ortholog	XM_002692069 /// XM_590767	-4.9	5.5	7.8
SCUBE2	signal peptide, CUB domain, EGF-like 2	XM_002693059 ///	-4.9	5.3	7.6

		XM_003583105 /// XM_003586953 /// XM_608409			
COBLL1	COBL-like 1	XM_002685374 /// XM_610577	-4.9	4.9	7.2
APOA1	apolipoprotein A-I	NM_174242	-4.9	9.6	11.9
RFTN1	raftlin, lipid raft linker 1	XM_002685189 /// XM_583278	-4.9	6.0	8.3
RASSF3	Ras association (RalGDS/AF-6) domain family member 3	NM_001192886	-5.0	6.3	8.6
FOLH1	folate hydrolase (prostate-specific membrane antigen) 1	NM_001101858	-5.0	7.0	9.3
ROBO4	roundabout homolog 4, magic roundabout (Drosophila)	NM_001099191	-5.0	5.9	8.2
PLAC8	placenta-specific 8	NM_001076987	-5.0	4.7	7.0
CORO1A	coronin, actin binding protein, 1A	NM_174521	-5.0	4.0	6.3
UACA	uveal autoantigen with coiled-coil domains and ankyrin repeats	NM_174209	-5.0	6.1	8.4
FXYD5	FXYD domain containing ion transport regulator 5	NM_001192063	-5.0	4.7	7.0
SCHIP1	schwannomin interacting protein 1	NM_001046412	-5.0	6.0	8.4
TENC1	tensin like C1 domain containing phosphatase (tensin 2)	XM_001790201 /// XM_002687243	-5.0	4.9	7.2
SIPA1	signal-induced proliferation-associated 1	NM_001101895	-5.0	6.0	8.3
PGM5	phosphoglucomutase 5	NM_001102335	-5.0	4.8	7.1
SERPINB8	serpin peptidase inhibitor, clade B (ovalbumin), member 8	NM_001035287	-5.0	5.1	7.4
IFI27	putative ISG12(a) protein	NM_001038050	-5.0	7.3	9.7
RGCC	regulator of cell cycle	NM_001102276	-5.0	5.3	7.6
BOLA-DRB3 /// LOC100851018	major histocompatibility complex, class II, DRB3 /// DLA class II histocompatibility an	NM_001012680 /// XM_003585221	-5.1	5.3	7.7
TSPAN2	tetraspanin 2	NM_001034657	-5.1	3.6	5.9
C16H1orf21	chromosome 16 open reading frame, human C1orf21	NM_001081547	-5.1	5.7	8.0
C26H10orf32 /// LOC100852300	chromosome 26 open reading frame, human C10orf32 /// UPF0693 protein C10orf32 homolog	XM_002698498 /// XM_003584090 /// XM_003585335	-5.1	6.7	9.1
KDELR3	KDEL (Lys-Asp-Glu-Leu) endoplasmic reticulum protein	NM_001192233	-5.1	8.3	10.6

	retention receptor 3				
MAGI1	membrane associated guanylate kinase, WW and PDZ domain containing 1	XM_001789358 /// XM_001789365 /// XM_002696963 /// XM_003583802	-5.1	7.1	9.4
TSC22D3	TSC22 domain family, member 3	NM_001103342	-5.1	7.4	9.8
FABP5	fatty acid binding protein 5 (psoriasis-associated)	NM_174315	-5.1	5.1	7.4
FHOD1	formin homology 2 domain containing 1	NM_001206339	-5.1	5.3	7.7
RGS1	regulator of G-protein signaling 1	NM_001199063	-5.1	3.5	5.9
USP53	ubiquitin specific peptidase 53	XM_003582305 /// XM_003586177 /// XM_003586178	-5.1	7.7	10.1
ASS1	argininosuccinate synthase 1	NM_173892	-5.1	6.0	8.4
MYCBP2	MYC binding protein 2	NM_001192817	-5.1	7.0	9.3
CPQ	plasma glutamate carboxypeptidase	NM_001076248	-5.2	5.2	7.6
MDK /// MDK	midkine-like /// midkine (neurite growth-promoting factor 2)	NM_173935 /// XM_002694376 /// XM_870711	-5.2	5.9	8.3
LOC100849927 /// TINAGL1	tubulointerstitial nephritis antigen-like /// tubulointerstitial nephritis antigen-like	XM_002685619 /// XM_003585097 /// XM_882308	-5.2	3.8	6.2
TGM2	transglutaminase 2 (C polypeptide, protein-glutamine-gamma-glutamyltransferase)	NM_177507	-5.2	8.1	10.5
PVR	poliovirus receptor	XM_002695088 /// XM_605244	-5.2	5.2	7.6
STK10	serine/threonine kinase 10	NM_001192627	-5.2	6.0	8.4
ICAM1	intercellular adhesion molecule 1	NM_174348	-5.2	5.4	7.8
PYROXD2	pyridine nucleotide-disulphide oxidoreductase domain 2	NM_001034532	-5.3	4.9	7.3
SEMA5B	sema domain, seven thrombospondin repeats (type 1 and type 1-like), transmembrane domai	XM_001788280 /// XM_002684809	-5.3	5.1	7.5
SPTBN1	spectrin, beta, non-erythrocytic 1	NM_001192276	-5.3	9.1	11.5
ANKRD50	ankyrin repeat domain 50	NM_001205949	-5.3	7.3	9.8
LBH	limb bud and heart development homolog (mouse)	NM_001099152	-5.3	5.3	7.7
MFNG	MFNG O-fucosylpeptide 3-beta-N-	NM_001101051	-5.3	5.0	7.4

	acetylglucosaminyltransferase				
PSMB9	proteasome (prosome, macropain) subunit, beta type, 9 (large multifunctional peptidase	NM_001034388	-5.4	7.2	9.6
KLF4	Kruppel-like factor 4 (gut)	NM_001105385	-5.4	4.9	7.4
LOC100137759	N-acetyl-beta-glucosaminyl-glycoprotein 4-beta-N-acetylgalactosaminyltransferase 1-like	XM_003584770 /// XM_003588069	-5.4	3.9	6.4
EFNA1	ephrin-A1	NM_001034292	-5.4	5.6	8.0
ROBO1	roundabout, axon guidance receptor, homolog 1 (Drosophila)	NM_001192888	-5.4	6.3	8.8
LHFPL2	lipoma HMGIC fusion partner-like 2	NM_001099151	-5.4	5.5	8.0
PLAGL1	pleiomorphic adenoma gene-like 1	NM_001103289	-5.4	5.8	8.3
KIAA1522	KIAA1522 ortholog	XM_001790114 /// XM_002685609 /// XM_003581882 /// XM_003585785	-5.5	7.0	9.5
LOC520070	endosialin-like	XM_003584268 /// XM_003588045	-5.5	4.5	7.0
TIMP1	TIMP metallopeptidase inhibitor 1	NM_174471	-5.5	7.6	10.1
GSN	gelsolin	NM_001034627 /// NM_001113284	-5.5	8.3	10.8
FGL1	fibrinogen-like 1	NM_001034313	-5.5	4.4	6.9
USF1	upstream transcription factor 1	NM_001001161	-5.5	4.7	7.2
MIR568	microRNA mir-568	NR_031331	-5.5	6.9	9.3
PRKCDBP	protein kinase C, delta binding protein	NM_001083401	-5.6	6.4	8.9
BOLA-DQA2	major histocompatibility complex, class II, DQ alpha 2	NM_001012681	-5.6	4.5	7.0
GJB5	gap junction protein, beta 5, 31.1kDa	NM_001205907	-5.6	6.4	8.9
DSC2	desmocollin 2	NM_001166526	-5.6	4.7	7.2
FHL2	four and a half LIM domains 2	NM_001046046	-5.6	8.2	10.7
REG4	regenerating islet-derived family, member 4	NM_001076986	-5.6	4.1	6.6
PLXNB2	plexin B2	NM_001205629	-5.6	5.0	7.5
CLIC4	chloride intracellular channel 4	NM_001080218	-5.7	7.6	10.1
CTSC	cathepsin C	NM_001033617	-5.7	7.4	9.9
SMARCA2	SWI/SNF related, matrix associated, actin dependent	NM_001099115	-5.7	7.6	10.1

	regulator of chromatin, subfamily a				
AOX1	aldehyde oxidase 1	NM_176668	-5.7	6.3	8.8
SERTAD4	SERTA domain containing 4	NM_001193037	-5.7	4.3	6.8
C1S	complement component 1, s subcomponent	NM_001076550	-5.7	4.6	7.1
TGFBR3	transforming growth factor, beta receptor III	XM_001253071 /// XM_002686233	-5.7	8.2	10.7
LOC100851799 /// SLC40A1	solute carrier family 40 member 1-like /// solute carrier family 40 (iron-regulated tra	NM_001077970 /// XM_003581794	-5.7	6.8	9.3
BOLA-DMA	major histocompatibility complex, class II, DM alpha	NM_001012674	-5.7	4.5	7.0
RBFOX2	RNA binding protein, fox-1 homolog (C. elegans) 2	NM_001205372	-5.7	6.0	8.5
KCTD15	potassium channel tetramerisation domain containing 15	NM_001075568	-5.7	5.2	7.7
KLHL23	kelch-like 23 (Drosophila)	NM_001205507	-5.7	5.1	7.6
IPO11	importin 11	NM_001192932	-5.8	6.6	9.1
CD200	CD200 molecule	NM_001034620	-5.8	4.1	6.6
C1QC	complement component 1, q subcomponent, C chain	NM_001206396	-5.8	4.5	7.0
GPRC5C	G protein-coupled receptor, family C, group 5, member C	NM_001272010 /// XM_002696193 /// XM_873143	-5.8	4.8	7.4
PDE4DIP	phosphodiesterase 4D interacting protein	NM_001101902	-5.8	5.0	7.6
CCND3	cyclin D3	NM_001034709	-5.8	7.3	9.9
MEOX2	mesenchyme homeobox 2	NM_001098045	-5.8	3.9	6.5
ACVR1	activin A receptor, type I	NM_176663	-5.8	5.6	8.2
ICAM3	intercellular adhesion molecule 3	NM_174349	-5.9	4.5	7.1
CP	ceruloplasmin (ferroxidase)	NM_001256556 /// XM_002685026 /// XM_592003	-5.9	3.4	5.9
SLC1A3	solute carrier family 1 (glial high affinity glutamate transporter), member 3	NM_174600	-5.9	3.7	6.2
MAP1B	microtubule-associated protein 1B	NM_001206119	-5.9	5.3	7.9
MICAL1	microtubule associated monoxygenase, calponin and LIM domain containing 1	NM_001081582	-5.9	4.6	7.1
C1QB	complement component 1, q subcomponent, B chain	NM_001046599	-5.9	4.9	7.4
ISLR	immunoglobulin superfamily containing leucine-rich repeat	NM_001080729	-5.9	5.0	7.6

CXCL3	chemokine (C-X-C motif) ligand 3	NM_001046513	-6.0	4.9	7.4
ECSCR	endothelial cell-specific chemotaxis regulator	NM_001046099 /// NM_001244445 /// NM_001244446	-6.0	5.8	8.4
TIMP3	TIMP metallopeptidase inhibitor 3	NM_174473	-6.0	5.3	7.9
ANGPTL4	angiopoietin-like 4	NM_001046043	-6.0	5.1	7.7
IMPA2	inositol(myo)-1(or 4)-monophosphatase 2	NM_001192282	-6.0	7.2	9.8
TCN2	transcobalamin II	NM_174195	-6.0	5.9	8.5
ERRFI1	ERBB receptor feedback inhibitor 1	NM_001077930	-6.0	7.3	9.9
PRSS2	protease, serine, 2 (trypsin 2)	NM_174690	-6.0	4.6	7.2
RSRC2	arginine/serine-rich coiled-coil 2	NM_001102146	-6.1	6.0	8.6
LYVE1	lymphatic vessel endothelial hyaluronan receptor 1	NM_205815	-6.1	5.1	7.7
NCOR2	nuclear receptor corepressor 2	XM_003583311 /// XM_003584904 /// XM_003587159 /// XM_581058	-6.1	8.0	10.6
ARHGAP18	Rho GTPase activating protein 18	NM_001110772	-6.1	4.8	7.4
Sep-08	septin 8	NM_001076230	-6.1	7.7	10.3
FOXS1	forkhead box S1	NM_001099716	-6.2	4.1	6.7
MXRA7	matrix-remodelling associated 7	XM_001253301 /// XM_002696202 /// XM_003583641 /// XM_003583642 /// XM_003587485 /// XM	-6.2	8.8	11.4
DUSP7	dual specificity phosphatase 7	NM_001101294	-6.3	5.2	7.8
LRRC34	leucine rich repeat containing 34	XM_002684932 /// XM_590280	-6.3	3.8	6.5
COL13A1	collagen, type XIII, alpha 1	NM_001105433	-6.3	4.7	7.4
CFD	complement factor D (adipsin)	NM_001034255	-6.3	4.4	7.0
SLC5A11	solute carrier family 5 (sodium/glucose cotransporter), member 11	NM_001034660	-6.3	4.0	6.7
BICC1	bicaudal C homolog 1 (Drosophila)	XM_002698825 /// XM_617983	-6.3	4.5	7.2

LOC504773	regakine 1	NM_001034220	-6.3	5.5	8.1
ANXA2	annexin A2	NM_174716	-6.4	8.4	11.1
CFH	complement factor H	NM_001033936	-6.4	3.6	6.3
SLC9A3R2	solute carrier family 9 (sodium/hydrogen exchanger), member 3 regulator 2	NM_001077065	-6.4	5.5	8.2
ENDOD1	endonuclease domain containing 1	NM_001102519	-6.5	4.9	7.6
TNFRSF1A	tumor necrosis factor receptor superfamily, member 1A	NM_174674	-6.5	6.5	9.2
LAPTM5	lysosomal protein transmembrane 5	NM_001046118	-6.5	4.8	7.5
RNASE6	ribonuclease, RNase A family, k6	NM_174594	-6.5	4.9	7.6
BT.106027 /// LOC783653	--- /// ---	---	-6.5	3.6	6.3
OCLN	occludin	NM_001082433	-6.6	5.0	7.7
KLF2	Kruppel-like factor 2 (lung)	XM_001787366 /// XM_002688582	-6.6	5.3	8.0
SELENBP1	selenium binding protein 1	NM_001046048	-6.6	5.1	7.8
OCIAD2	OCIA domain containing 2	NM_001034258	-6.6	5.2	7.9
NR2F2	nuclear receptor subfamily 2, group F, member 2	NM_174402	-6.7	7.9	10.7
CXCR7	chemokine (C-X-C motif) receptor 7	NM_001098381	-6.7	5.7	8.5
CXCL16	chemokine (C-X-C motif) ligand 16	NM_001046095	-6.7	5.0	7.8
ABLIM1	actin binding LIM protein 1	XM_002698530 /// XM_866358	-6.7	6.4	9.1
ACTG2	actin, gamma 2, smooth muscle, enteric	NM_001013592	-6.7	7.6	10.4
CD44	CD44 molecule (Indian blood group)	NM_174013	-6.8	3.4	6.1
CHST2	carbohydrate (N-acetylglucosamine-6-O) sulfotransferase 2	NM_001113769	-6.8	3.5	6.3
ITGBL1	integrin, beta-like 1 (with EGF-like repeat domains)	NM_001206834	-6.8	5.6	8.3
BGN	biglycan	NM_178318	-6.8	6.0	8.8
FHL3	four and a half LIM domains 3	NM_001034223	-6.8	5.1	7.8
MS4A8B	membrane-spanning 4-domains, subfamily A, member 8B	NM_001034056	-6.9	7.5	10.3
SHANK3	SH3 and multiple ankyrin repeat domains 3	XM_002687990 /// XM_589942	-6.9	5.4	8.2
ARHGEF3	Rho guanine nucleotide exchange factor (GEF) 3	XM_001250879 /// XM_002697002 /// XM_003583806 ///	-6.9	4.0	6.8

		XM_003583807 /// XM_003587632 /// XM			
IGFBP3	insulin-like growth factor binding protein 3	NM_174556 /// XM_003582113	-7.0	6.2	9.0
RHOU	ras homolog gene family, member U	NM_001098147	-7.0	4.9	7.7
PRKAG2	protein kinase, AMP-activated, gamma 2 non-catalytic subunit	XM_002686979 /// XM_002704191	-7.0	5.4	8.2
TRPM4	transient receptor potential cation channel, subfamily M, member 4	XM_002695205 /// XM_002701936	-7.0	4.2	7.0
QSOX1	quiescin Q6 sulfhydryl oxidase 1	NM_001102074	-7.1	6.6	9.4
SPARC	secreted protein, acidic, cysteine-rich (osteonectin)	NM_174464	-7.1	9.2	12.0
RBMS2	RNA binding motif, single stranded interacting protein 2	NM_001034365	-7.1	5.0	7.8
FAM43A	family with sequence similarity 43, member A	XM_002684829 /// XM_586734	-7.2	4.8	7.6
TPBG	trophoblast glycoprotein	XM_002690047 /// XM_593502	-7.2	6.6	9.4
ADAM19	ADAM metallopeptidase domain 19	NM_001075475	-7.2	5.1	7.9
MALL	mal, T-cell differentiation protein-like	NM_001046115	-7.2	3.5	6.3
FAM105A	family with sequence similarity 105, member A	NM_001102171	-7.2	6.3	9.1
ADAMTS4	ADAM metallopeptidase with thrombospondin type 1 motif, 4	NM_181667	-7.2	4.7	7.5
CYB5R3	cytochrome b5 reductase 3	NM_001103250	-7.3	8.5	11.4
PQLC3	PQ loop repeat containing 3	NM_001101878	-7.3	4.7	7.5
JAG1	jagged 1	NM_001191178	-7.3	6.5	9.3
TAGLN2	transgelin 2	NM_001013599	-7.3	6.8	9.6
FRMD4A	FERM domain containing 4A	NM_001192267	-7.3	4.9	7.8
CD9	CD9 molecule	NM_173900	-7.3	7.7	10.6
DPYD	dihydropyrimidine dehydrogenase	NM_174041	-7.3	5.7	8.6
KANK2	KN motif and ankyrin repeat domains 2	NM_001076531	-7.3	6.2	9.1
SH3BP5	SH3-domain binding protein 5 (BTK-associated)	NM_001206288	-7.3	5.1	8.0
VCAM1	vascular cell adhesion molecule 1	NM_174484	-7.4	4.7	7.6
GNG2	guanine nucleotide binding protein (G protein), gamma 2	NM_174072	-7.5	5.8	8.7
NCAM1	neural cell adhesion molecule 1	NM_174399	-7.5	4.9	7.8

LOC100851458	CUGBP Elav-like family member 2-like	XM_003582899	-7.5	3.8	6.7
ARPC1B	actin related protein 2/3 complex, subunit 1B, 41kDa	NM_001014844	-7.5	5.5	8.5
IQSEC1	IQ motif and Sec7 domain 1	NM_001206814	-7.6	5.1	8.1
FRMD6 /// LOC100335364	FERM domain containing 6 /// FERM domain-containing protein 6-like	NM_001102133 /// XM_002700610	-7.6	4.6	7.5
LYZ	lysozyme	NM_001078159	-7.6	3.9	6.8
CCDC85B	coiled-coil domain containing 85B	NM_001144087	-7.6	3.8	6.8
BLA-DQB /// LA-DQB /// LOC100851058	MHC class II antigen /// MHC cell surface glycoprotein /// rano class II histocompatibi	NM_001034668 /// NM_001080923 /// XM_003585246	-7.7	3.4	6.3
LMNA	lamin A/C	NM_001034053	-7.7	5.6	8.5
OSTF1	osteoclast stimulating factor 1	NM_174409	-7.7	7.2	10.1
PEAR1	platelet endothelial aggregation receptor 1	NM_001101300	-7.7	5.2	8.1
ATRX	alpha thalassemia/mental retardation syndrome X-linked	XM_002699982 /// XM_002699983 /// XM_002707277 /// XM_592333	-7.8	5.7	8.6
C29H11orf75 /// SMCO4	chromosome 29 open reading frame, human C11orf75 /// single-pass membrane protein with	XM_002698992 /// XM_002703411 /// XM_002706160	-7.8	5.1	8.1
ID3	inhibitor of DNA binding 3, dominant negative helix-loop-helix protein	NM_001014950	-7.8	6.7	9.6
ERG	v-ets erythroblastosis virus E26 oncogene homolog (avian)	NM_001102183	-7.8	4.7	7.7
CCL14	chemokine (C-C motif) ligand 14	NM_001046585	-7.8	5.0	7.9
OSR2	odd-skipped related 2 (Drosophila)	NM_001034328	-7.8	4.2	7.1
CAST	calpastatin	NM_001030318 /// NM_001030319 /// NM_001030320 /// NM_174003	-7.9	6.8	9.8
EMP1	epithelial membrane protein 1	XM_001251759 /// XM_002687764	-7.9	4.8	7.8
CLDN5	claudin 5	NM_001076460	-7.9	4.8	7.8
HEPH	hephaestin	XM_002700167 /// XM_003584900 ///	-7.9	4.7	7.7

		XM_587920			
CDH5	cadherin 5, type 2 (vascular endothelium)	NM_001001601	-8.0	5.5	8.5
C1QTNF7	C1q and tumor necrosis factor related protein 7	NM_001076201	-8.0	4.1	7.1
TMEM150C	transmembrane protein 150C	NM_001078001	-8.0	3.8	6.8
PRPF38B	PRP38 pre-mRNA processing factor 38 (yeast) domain containing B	XM_002686159 /// XM_003584875	-8.0	4.9	7.9
GGTA1	alpha-galactosyltransferase 1 (glycoprotein)	NM_177511	-8.0	4.9	8.0
APLNR	apelin receptor	NM_001102524	-8.1	4.5	7.5
TMEM88	transmembrane protein 88	NM_001098378	-8.1	5.4	8.4
FERMT2	fermitin family member 2	NM_001101264	-8.1	6.9	9.9
PLIN5	perilipin 5	NM_001101136	-8.2	4.1	7.2
FBLIM1	filamin binding LIM protein 1	NM_001076955	-8.2	6.3	9.4
WFDC1	WAP four-disulfide core domain 1	XM_002694744 /// XM_581642	-8.3	5.8	8.9
PTGIS	prostaglandin I2 (prostacyclin) synthase	NM_174444	-8.4	4.6	7.7
TACC2	transforming, acidic coiled-coil containing protein 2	NM_001102159 /// XM_003584113 /// XM_003584114 /// XM_003587903	-8.4	5.2	8.3
CYR61	cysteine-rich, angiogenic inducer, 61	NM_001034340	-8.4	7.2	10.2
AHNAK	---	---	-8.4	7.4	10.5
DACT1	dapper, antagonist of beta-catenin, homolog 1 (Xenopus laevis)	XM_002690959 /// XM_002690960 /// XM_002700646	-8.4	5.3	8.3
REM1	RAS (RAD and GEM)-like GTP-binding 1	NM_001046001	-8.4	3.2	6.3
LOC787239	DKFZP459P193 protein-like	XM_001254697	-8.5	5.0	8.1
CASP4	caspase 4, apoptosis-related cysteine peptidase	NM_176638	-8.5	4.2	7.3
LRCH1	leucine-rich repeats and calponin homology (CH) domain containing 1	XM_002691830 /// XM_581598	-8.5	3.7	6.8
LDB2	LIM domain binding 2	NM_001046611	-8.5	5.3	8.4
LRRC70	leucine rich repeat containing 70	XM_002696307 /// XM_002702499	-8.5	5.1	8.2
ME3	malic enzyme 3, NADP(+)-dependent, mitochondrial	NM_001075877	-8.6	3.9	7.0

TMEM119	transmembrane protein 119	NM_001083664	-8.9	5.7	8.8
PLN	phospholamban	NM_001103319	-8.9	4.2	7.3
COL18A1	collagen, type XVIII, alpha 1	NM_001083388	-8.9	7.1	10.3
C4A /// LOC100851057 /// LOC100852118 /// LOC617696	complement component 4A /// complement C4-A-like /// complement C4-A-like /// complemen	NM_001166485 /// XM_003584610 /// XM_003584815 /// XM_870004	-8.9	7.3	10.4
EMCN	endomucin	NM_001076420	-9.0	5.8	9.0
CD34	CD34 molecule	NM_174009	-9.2	4.9	8.1
CYBRD1	cytochrome b reductase 1	NM_001206049	-9.3	5.8	9.0
LMO2	LIM domain only 2 (rhombotin-like 1)	NM_001076352	-9.3	4.9	8.1
RND3	Rho family GTPase 3	NM_001191158 /// XM_003585297	-9.3	5.5	8.8
FRZB	frizzled-related protein	NM_174059	-9.3	4.2	7.4
LPAR6	lysophosphatidic acid receptor 6	NM_001101284	-9.3	3.8	7.0
TIMP2	TIMP metallopeptidase inhibitor 2	NM_174472	-9.4	9.1	12.4
AGTR1	angiotensin II receptor, type 1	NM_174233	-9.4	4.3	7.5
OLFML3	olfactomedin-like 3	NM_001075197	-9.4	5.9	9.1
DHRS7	dehydrogenase/reductase (SDR family) member 7	NM_001046162	-9.4	3.7	6.9
LMCD1	LIM and cysteine-rich domains 1	NM_001076222	-9.5	5.1	8.4
LUC7L3	LUC7-like 3 (S. cerevisiae)	NM_001034684	-9.6	5.5	8.8
C1QA	complement component 1, q subcomponent, A chain	NM_001014945	-9.6	5.4	8.7
TGFB2	transforming growth factor, beta 2	NM_001113252	-9.7	3.5	6.8
TCF4	transcription factor 4	NM_001034621	-9.8	4.7	8.0
HFM1	HFM1, ATP-dependent DNA helicase homolog (S. cerevisiae)	NM_001205576	-9.8	3.2	6.5
WIPF1	WAS/WASL interacting protein family, member 1	NM_001076923	-9.8	5.1	8.4
GNB4	guanine nucleotide binding protein (G protein), beta polypeptide 4	NM_001099033	-9.8	3.3	6.6
LOC539690	complement component C1q receptor-like	XM_003582935 /// XM_003586784	-9.9	5.6	8.9
ALDH1A2	aldehyde dehydrogenase 1 family, member A2	XM_002690855 ///	-10.0	5.1	8.5

		XM_615062			
LIMA1	LIM domain and actin binding 1	NM_001192754	-10.0	5.3	8.6
AFAP1L2	actin filament associated protein 1-like 2	NM_001076375	-10.1	7.0	10.3
ARHGDIB	Rho GDP dissociation inhibitor (GDI) beta	NM_175797	-10.2	5.4	8.7
CHST7	carbohydrate (N-acetylglucosamine 6-O) sulfotransferase 7	NM_001193203	-10.4	4.9	8.3
ABCB1 /// ABCB4 /// LOC100296627 /// LOC785554	ATP-binding cassette, sub-family B (MDR/TAP), member 1 /// ATP-binding cassette, sub-fa	XM_002686717 /// XM_002686730 /// XM_002686731 /// XM_003582070 /// XM_003584756 /// XM	-10.5	5.3	8.7
PEG3	paternally expressed 3	NM_001002887 /// XM_003583503 /// XM_003587348	-10.6	7.6	11.0
SPRY1	sprouty homolog 1, antagonist of FGF signaling (Drosophila)	NM_001099366	-10.7	5.0	8.5
ELTD1	EGF, latrophilin and seven transmembrane domain containing 1	NM_001076908	-10.7	5.3	8.7
C1QTNF5	C1q and tumor necrosis factor related protein 5	NM_001099138	-10.9	4.3	7.8
KCNE4	potassium voltage-gated channel, Isk-related family, member 4	NM_001081543	-11.0	5.9	9.3
TSPAN33	tetraspanin 33	NM_001034672	-11.0	3.4	6.8
EHD4	EH-domain containing 4	NM_001192053	-11.1	4.5	7.9
SH3BGR	SH3 domain binding glutamic acid-rich protein	NM_001243326	-11.2	5.7	9.2
GPR116	G protein-coupled receptor 116	NM_001193243	-11.2	4.6	8.1
BASP1	brain abundant, membrane attached signal protein 1	NM_174780	-11.2	5.8	9.3
LOC100297676	C-type lectin domain family 2 member G-like	XM_002687838 /// XM_002704428	-11.2	4.8	8.3
MEG3	maternally expressed 3 (non-protein coding)	NR_037684	-11.3	5.0	8.5
CDH11 /// LOC100851861	cadherin 11, type 2, OB-cadherin (osteoblast) /// cadherin-11-like	NM_001081624 /// XR_138938	-11.3	7.1	10.6
AEBP1	AE binding protein 1	NM_174839	-11.4	4.9	8.4
SVIL	supervillin	NM_174190	-11.5	5.6	9.1
CXCL10	chemokine (C-X-C motif) ligand 10	NM_001046551	-11.6	4.0	7.6

NUAK1	NUAK family, SNF1-like kinase, 1	NM_001205496	-11.6	5.3	8.8
TEK	TEK tyrosine kinase, endothelial	NM_173964	-11.6	4.8	8.3
LRRFIP1	leucine rich repeat (in FLII) interacting protein 1	NM_001102308	-11.7	4.8	8.3
IFI30	interferon, gamma-inducible protein 30	NM_001101251	-11.7	4.8	8.4
ANXA3	annexin A3	NM_001035325	-11.7	5.0	8.5
C7	complement component 7	NM_001045966	-11.8	5.0	8.6
LOC518495	apolipoprotein L3-like	XM_003582237 /// XM_003586098	-12.0	4.3	7.9
ACTN1	actinin, alpha 1	NM_001035351	-12.0	6.4	10.0
COL6A1	collagen, type VI, alpha 1	NM_001143865	-12.3	7.8	11.4
TFPI2	tissue factor pathway inhibitor 2	NM_182788	-12.4	4.2	7.8
PECAM1	platelet/endothelial cell adhesion molecule	NM_174571	-12.4	4.8	8.4
SGK1	serum/glucocorticoid regulated kinase 1	NM_001102033	-12.4	4.5	8.1
GYPC	glycophorin C (Gerbich blood group)	NM_001002886	-12.7	6.5	10.2
FBN1	fibrillin 1	NM_174053	-12.9	6.9	10.6
PDK4	pyruvate dehydrogenase kinase, isozyme 4	NM_001101883	-13.1	5.1	8.8
NR2F1	nuclear receptor subfamily 2, group F, member 1	NM_175804	-13.2	5.9	9.6
COL16A1 /// LOC100849968	collagen, type XVI, alpha 1 /// collagen alpha-1(XVI) chain-like	XM_002685618 /// XM_003585098 /// XM_583549	-13.2	7.3	11.0
NFIA	nuclear factor I/A	NM_001038209	-13.2	5.8	9.5
AS3MT /// AS3MT	arsenic (+3 oxidation state) methyltransferase /// arsenic (+3 oxidation state) methylt	NM_001035023 /// XM_001255467	-13.3	5.3	9.1
ENPP2	ectonucleotide pyrophosphatase/phosphodiesterase 2	NM_001080293	-13.3	4.7	8.5
AFAP1L1	actin filament associated protein 1-like 1	NM_001100334	-13.3	3.1	6.9
PTX3	pentraxin 3, long	NM_001076259	-13.4	3.6	7.4
SOX18	SRY (sex determining region Y)-box 18	NM_001075789	-13.5	4.4	8.2
ADAMTS1	ADAM metallopeptidase with thrombospondin type 1 motif, 1	NM_001101080	-13.6	5.9	9.6
ZFP36L1	zinc finger protein 36, C3H type-like 1	NM_001101234	-13.6	7.2	11.0
CTSK	cathepsin K	NM_001034435	-13.7	6.3	10.1
GRB10	growth factor receptor-bound protein 10	NM_001192586	-13.7	3.9	7.7
THBS2	thrombospondin 2	NM_176872	-13.8	5.7	9.5

DAB2	disabled homolog 2, mitogen-responsive phosphoprotein (Drosophila)	NM_001193246	-13.9	5.4	9.2
SPATS2L	spermatogenesis associated, serine-rich 2-like	NM_001192987	-13.9	6.1	9.9
IFITM1	interferon induced transmembrane protein 1 (9-27)	NM_001078142	-14.0	7.7	11.5
VIPR2	vasoactive intestinal peptide receptor 2	NM_001206781	-14.1	6.1	9.9
CAPG	capping protein (actin filament), gelsolin-like	NM_178574	-14.1	4.7	8.5
LOC100850955	myosin light chain kinase, smooth muscle-like	XM_003581710	-14.2	7.6	11.5
EMILIN1	elastin microfibril interfacer 1	NM_001192434	-14.2	5.5	9.3
EPAS1	endothelial PAS domain protein 1	NM_174725	-14.4	6.5	10.4
AGRN	agrin	XM_002694193 /// XM_604151	-14.6	5.8	9.7
CXCL12	chemokine (C-X-C motif) ligand 12	NM_001113174	-14.7	4.8	8.7
PMP22	peripheral myelin protein 22	NM_001101156	-14.8	6.1	10.0
COL11A1	collagen, type XI, alpha 1	NM_001166509	-14.9	4.5	8.4
COL1A1	collagen, type I, alpha 1	NM_001034039	-14.9	7.0	10.9
CYP17A1	cytochrome P450, subfamily XVII	NM_174304	-15.0	5.1	9.0
CD302	CD302 molecule	NM_001110191	-15.1	4.1	8.1
BRB	brain ribonuclease	NM_173891	-15.3	3.4	7.4
KDR	kinase insert domain receptor (a type III receptor tyrosine kinase)	NM_001110000	-15.3	4.5	8.5
COLEC11	collectin sub-family member 11	NM_001076303	-15.4	4.7	8.7
PSAT1	phosphoserine aminotransferase 1	NM_001102150	-15.4	6.3	10.3
TM4SF18	transmembrane 4 L six family member 18	NM_001034287 /// NM_001184724	-15.4	5.0	8.9
PTH1R	parathyroid hormone 1 receptor	NM_001075332	-15.6	4.1	8.1
LAMA4	laminin, alpha 4	NM_001205965	-15.9	4.7	8.7
RGS5	regulator of G-protein signaling 5	NM_001034707	-16.0	5.4	9.4
LOC783195 /// RNASE4	ribonuclease 4-like /// ribonuclease, RNase A family, 4	NM_001040590 /// XM_001251830 /// XM_002690653	-16.2	5.2	9.3
IGFBP4	insulin-like growth factor binding protein 4	NM_174557	-16.5	5.8	9.8
MMP23B	matrix metallopeptidase 23B	NM_001038556	-16.8	5.4	9.4
EVA1B	eva-1 homolog B (C. elegans)	NM_001101271	-16.9	5.4	9.4

GNG11	guanine nucleotide binding protein (G protein), gamma 11	NM_001024523	-17.2	5.5	9.6
INSL3	insulin-like 3 (Leydig cell)	NM_174365	-17.3	8.8	12.9
COL4A1	collagen, type IV, alpha 1	NM_001166511	-17.4	8.5	12.6
CLEC14A	C-type lectin domain family 14, member A	NM_001077890	-17.6	3.9	8.0
SCARA5	scavenger receptor class A, member 5 (putative)	NM_001102499	-17.8	3.7	7.8
FNBP1	formin binding protein 1	NM_001206712	-18.0	4.4	8.6
TM4SF1	transmembrane 4 L six family member 1	NM_001075980	-18.0	4.8	9.0
NFIB	nuclear factor I/B	NM_001076104	-18.5	5.0	9.2
VSTM4	V-set and transmembrane domain containing 4	XM_002698947 /// XM_003584215	-18.5	4.2	8.4
MAN1C1	mannosidase, alpha, class 1C, member 1	XM_002685706 /// XM_866713	-18.5	5.5	9.7
PHLDB2	pleckstrin homology-like domain, family B, member 2	NM_001206308	-18.6	4.2	8.4
FMOD	fibromodulin	NM_174058	-18.7	5.8	10.0
LOC100337023	collagen alpha-1(V) chain-like	XR_139433	-18.8	6.9	11.2
TMEM204	transmembrane protein 204	NM_001076377	-18.9	4.7	8.9
SULF2	sulfatase 2	NM_001192938	-18.9	4.2	8.5
TAGLN	transgelin	NM_001046149	-19.1	5.7	10.0
ANXA1	annexin A1	NM_175784	-19.3	6.7	11.0
SYTL2	synaptotagmin-like 2	NM_001102278	-19.5	3.5	7.8
DPT	dermatopontin	NM_001045903	-19.6	4.5	8.7
TCF21	transcription factor 21	NM_001014899	-19.7	6.3	10.6
GPM6A	glycoprotein M6A	NM_001075309	-19.7	3.7	8.0
LOC100336224 /// NEK6	serine/threonine-protein kinase Nek6-like /// NIMA (never in mitosis gene a)-related ki	NM_001098988 /// XM_002706994	-20.1	4.2	8.6
PXDN	peroxidasin homolog (Drosophila)	XM_002683948 /// XM_593953	-20.1	5.2	9.6
DPYSL3	dihydropyrimidinase-like 3	NM_001101068	-20.4	6.2	10.6
PLAT	plasminogen activator, tissue	NM_174146	-20.7	5.6	9.9
COLEC12	collectin sub-family member 12	NM_001101843	-20.7	4.1	8.5
CAV1	caveolin 1, caveolae protein, 22kDa	NM_174004	-20.9	5.1	9.4
DCLK1	doublecortin-like kinase 1	NM_001109962	-21.1	4.6	9.0

CTSH	cathepsin H	NM_001034385	-21.2	4.6	9.0
SNAI2	snail homolog 2 (Drosophila)	NM_001034538	-21.2	4.3	8.7
BOLA-DRB3	major histocompatibility complex, class II, DRB3	NM_001012680	-21.2	4.6	9.0
H19	H19, imprinted maternally expressed transcript (non-protein coding)	NR_003958	-21.3	5.8	10.2
BOLA-DRA	major histocompatibility complex, class II, DR alpha	NM_001012677	-21.3	5.2	9.6
STC1	stanniocalcin 1	NM_176669	-21.4	4.7	9.2
SDPR	serum deprivation response	XM_002685467 /// XM_610845	-21.4	5.1	9.5
CTSS	cathepsin S	NM_001033615	-21.9	4.9	9.3
SHISA2	shisa homolog 2 (Xenopus laevis)	NM_001101265	-22.0	5.7	10.2
CALCRL	calcitonin receptor-like	NM_001102107	-22.0	4.6	9.1
AXL	AXL receptor tyrosine kinase	XM_002695068 /// XM_594754	-22.4	4.3	8.8
HEYL	hairy/enhancer-of-split related with YRPW motif-like	NM_001024565	-22.7	4.4	8.9
NRK	Nik related kinase	XM_002699807	-24.1	3.8	8.4
LOC781493	collagen alpha-1(XIV) chain-like	XM_003583071 /// XM_003586917	-24.7	4.9	9.6
MMP2	matrix metallopeptidase 2 (gelatinase A, 72kDa gelatinase, 72kDa type IV collagenase)	NM_174745	-25.3	5.4	10.0
MATN2	matrilin 2	NM_001102528	-25.4	6.9	11.5
PRSS23	protease, serine, 23	NM_001080306	-25.7	6.2	10.9
PDLIM3	PDZ and LIM domain 3	NM_001034646	-26.1	4.0	8.7
AIM2	absent in melanoma 2	---	-26.4	3.4	8.1
CD99	CD99 molecule	NM_001244214	-26.5	5.1	9.9
CTGF	connective tissue growth factor	NM_174030	-26.8	6.7	11.4
COL27A1	collagen, type XXVII, alpha 1	NM_001206680	-27.5	5.8	10.6
LUM	lumican	NM_173934	-27.5	6.3	11.0
FAM101B	family with sequence similarity 101, member B	XM_002695711 /// XM_870793	-28.1	5.3	10.1
LAMB1	laminin, beta 1	NM_001206519	-28.2	5.6	10.4
LAMA2 /// LOC100848461	laminin, alpha 2 /// laminin subunit alpha-2-like	XM_001787958 /// XM_002690220 ///	-29.3	4.3	9.2

		XM_003585167 /// XM_003585378 /// XM_003586475			
SCG2	secretogranin II	NM_174176	-31.3	4.2	9.1
A2M	alpha-2-macroglobulin	NM_001109795	-31.3	5.2	10.1
LMO7	LIM domain 7	NM_001109801 /// XM_003582872 /// XM_003582873 /// XM_003586719 /// XM_003586720	-31.6	3.3	8.3
DKK3	dickkopf homolog 3 (Xenopus laevis)	NM_001100306	-31.7	4.2	9.2
FLI1	Friend leukemia virus integration 1	NM_001046298	-31.9	3.7	8.7
RARRES1	retinoic acid receptor responder (tazarotene induced) 1	NM_001075430	-32.0	4.3	9.3
LOXL1	lysyl oxidase-like 1	NM_174383	-32.1	4.0	9.0
XDH	xanthine dehydrogenase	NM_173972	-32.8	5.3	10.3
PDGFRA	platelet-derived growth factor receptor, alpha polypeptide	NM_001192345	-32.9	4.1	9.2
TBX3	T-box 3	XM_001787821 /// XM_002694588	-33.0	4.1	9.1
CDKN1C	cyclin-dependent kinase inhibitor 1C (p57, Kip2)	NM_001077903	-33.4	4.6	9.7
COL6A3	collagen, type VI, alpha 3	XM_002686570 /// XM_002686571 /// XM_002686573 /// XM_003582037 /// XM_003582038 /// XM	-33.5	6.3	11.3
PLK2	polo-like kinase 2	NM_001192245	-35.2	3.3	8.5
COL15A1	collagen, type XV, alpha 1	NM_001191285	-35.7	4.8	9.9
C27H8orf4	chromosome 27 open reading frame, human C8orf4	NM_001035490	-36.0	3.8	8.9
IGFBP6	insulin-like growth factor binding protein 6	NM_001040495	-36.2	5.9	11.1
RAMP2	receptor (G protein-coupled) activity modifying protein 2	NM_001098860	-36.5	5.0	10.2
ADAMDEC1 /// LOC100847471	ADAM-like, decysin 1 /// ADAM DEC1-like	NM_001206371 /// XM_002689785 /// XM_003582556 /// XM_003586413 /// XM_582254	-36.8	3.4	8.6

TGFBR2	transforming growth factor, beta receptor II (70/80kDa)	NM_001159566	-37.1	4.4	9.6
DLC1	deleted in liver cancer 1	NM_001102493	-38.6	4.0	9.3
FBLN5	fibulin 5	NM_001014946	-39.5	5.1	10.4
CLEC3B	C-type lectin domain family 3, member B	NM_001046212	-39.9	5.1	10.4
PLXND1	plexin D1	XM_001789172 /// XM_002697122	-40.5	3.9	9.3
STAR	steroidogenic acute regulatory protein	NM_174189	-40.7	4.5	9.8
COL12A1	collagen, type XII, alpha 1	NM_001206497	-42.5	5.0	10.4
SDC2	syndecan 2	NM_001034788	-43.4	4.6	10.1
HPGD	hydroxyprostaglandin dehydrogenase 15-(NAD)	NM_001034419	-43.7	5.1	10.5
ADAMDEC1	ADAM-like, decysin 1	NM_001206371 /// XM_002689785 /// XM_582254	-44.7	4.7	10.2
EGFLAM /// LOC100847583	EGF-like, fibronectin type III and laminin G domains /// pikachurin-like	NM_001083478 /// XM_003587531	-45.4	5.4	10.9
FILIP1L	filamin A interacting protein 1-like	XM_002684706 /// XM_002684707 /// XM_002702383 /// XM_003581681 /// XM_003581682 /// XM	-49.3	4.8	10.5
ID1	inhibitor of DNA binding 1, dominant negative helix-loop-helix protein	NM_001097568	-52.6	4.4	10.1
SPARCL1	SPARC-like 1 (hevin)	NM_001034302	-52.8	6.1	11.9
DUSP12	dual specificity phosphatase 12	XM_002685847 /// XM_581568	-53.9	4.7	10.5
ACTA2 /// ACTG2	actin, alpha 2, smooth muscle, aorta /// actin, gamma 2, smooth muscle, enteric	NM_001013592 /// NM_001034502	-55.0	6.1	11.9
LHFP	lipoma HMGIC fusion partner	NM_001077990	-55.5	4.4	10.2
NID1	nidogen 1	NM_001101155	-56.7	4.4	10.2
CXCL14	chemokine (C-X-C motif) ligand 14	NM_001034410	-65.9	5.0	11.0
IGF2	insulin-like growth factor 2 (somatomedin A)	NM_174087	-68.3	5.2	11.3
NID2	nidogen 2 (osteonidogen)	NM_001102065	-73.4	4.6	10.8
COL5A2	collagen, type V, alpha 2	XM_003581798 /// XM_003585717	-73.5	5.4	11.6

APOD	apolipoprotein D	NM_001076301	-74.3	4.9	11.1
OGN	osteoglycin	NM_173946	-75.4	4.6	10.8
COL3A1	collagen, type III, alpha 1	NM_001076831	-84.9	6.1	12.5
FN1	fibronectin 1	NM_001163778	-87.3	5.0	11.4
COL1A2	collagen, type I, alpha 2	NM_174520	-88.5	6.1	12.6
ALDH1A1	aldehyde dehydrogenase 1 family, member A1	NM_174239	-91.6	5.1	11.6
ASPN	asporin	NM_001034309	-96.9	4.0	10.6
DCN	decorin	NM_173906	-102.9	5.5	12.2
MGP	matrix Gla protein	NM_174707	-176.6	5.0	12.5
