# Supplementary material for: Transcriptome Comparisons Identify New Cell Markers for Theca Interna and Granulosa Cells from Small and Large Antral Ovarian Follicles
Source: PLoS One. 2015 Mar 16;10(3):e0119800. doi: 10.1371/journal.pone.0119800 (PMC4361622; doi:10.1371/journal.pone.0119800)
Supplement: S2 Table — Gene name, ID, fold change and mean array intensities are presented. (PDF) [file pone.0119800.s002.pdf]

**Table S2. List of genes 4-fold differentially expressed in granulosa cells compared with theca interna in large follicles with FDR $P < 0.05$. Gene name, ID, fold change and mean array intensities are presented.**

| Gene Symbol | Gene Title | RefSeq Transcript ID | Fold-change | Mean $\log_2$ array intensity | |
|---|---|---|---|---|---|
| | | | | Granulosa cells | Theca interna |
| TNFAIP6 | tumor necrosis factor, alpha-induced protein 6 | NM_001007813 | 319.3 | 12.0 | 3.7 |
| CYP19A1 | cytochrome P450, family 19, subfamily A, polypeptide 1 | NM_174305 | 147.0 | 12.8 | 5.6 |
| LRP8 | low density lipoprotein receptor-related protein 8, apolipoprotein e receptor | NM_001097565 | 107.3 | 10.7 | 3.9 |
| MGARP | chromosome 17 open reading frame, human C4orf49 | NM_001166611 | 42.3 | 11.4 | 6.0 |
| NR5A2 | nuclear receptor subfamily 5, group A, member 2 | NM_001206816 | 40.1 | 12.5 | 7.2 |
| SLC27A3 | solute carrier family 27 (fatty acid transporter), member 3 | XM_001790632 /// XM_002686020 | 39.1 | 9.6 | 4.3 |
| EFNA5 | ephrin-A5 | NM_001076432 | 37.0 | 10.0 | 4.8 |
| CHST8 | carbohydrate (N-acetylgalactosamine 4-0) sulfotransferase 8 | NM_001145992 | 30.0 | 10.7 | 5.8 |
| CA8 | carbonic anhydrase VIII | NM_001083690 | 27.6 | 8.6 | 3.9 |
| CLGN | calmegin | NM_001034205 | 27.1 | 10.0 | 5.2 |
| APOA2 | apolipoprotein A-II | NM_001045916 | 26.7 | 8.9 | 4.2 |
| GPX3 | glutathione peroxidase 3 (plasma) | NM_174077 | 23.8 | 10.0 | 5.4 |
| LRRC2 | leucine rich repeat containing 2 | NM_001080311 | 20.1 | 9.4 | 5.1 |
| SUSD4 | sushi domain containing 4 | NM_001101904 | 20.1 | 8.4 | 4.1 |
| FSHR | follicle stimulating hormone receptor | NM_174061 | 19.9 | 8.2 | 3.9 |
| CITED1 | Cbp/p300-interacting transactivator, with Glu/Asp-rich carboxy-terminal domain, 1 | NM_174518 | 17.6 | 10.6 | 6.4 |
| SPOCK2 | sparc/osteonectin, cwcv and kazal-like domains proteoglycan (testican) 2 | NM_001101115 | 16.6 | 9.2 | 5.1 |
| SLC35G1 | solute carrier family 35, member G1 | NM_001076470 | 15.7 | 10.5 | 6.6 |
| VCAN | versican | NM_181035 | 15.3 | 11.9 | 8.0 |
| TOX | thymocyte selection-associated high mobility group box | NM_001102096 | 14.9 | 10.2 | 6.3 |

| | | | | | |
|---|---|---|---|---|---|
| AP2B1 | adaptor-related protein complex 2, beta 1 subunit | NM_001075125 /// XM_003587384 | 14.4 | 7.4 | 3.5 |
| INHBA | inhibin, beta A | NM_174363 | 13.9 | 13.7 | 9.9 |
| RRAGD | Ras-related GTP binding D | NM_001192828 | 13.3 | 9.1 | 5.4 |
| BEX2 | brain expressed X-linked 2 | NM_001077034 | 13.2 | 12.1 | 8.4 |
| GLDC | glycine dehydrogenase (decarboxylating) | NM_001192951 | 13.0 | 7.4 | 3.7 |
| NABP1 | oligonucleotide/oligosaccharide-binding fold containing 2A | NM_001098124 | 12.9 | 8.2 | 4.5 |
| TFR2 | transferrin receptor 2 | NM_001177741 | 12.5 | 8.0 | 4.3 |
| IL6R | interleukin 6 receptor | NM_001110785 | 12.5 | 9.6 | 6.0 |
| SLC39A8 | solute carrier family 39 (zinc transporter), member 8 | NM_001205630 | 12.2 | 9.6 | 6.0 |
| IGSF11 | immunoglobulin superfamily, member 11 | NM_001076921 | 12.2 | 10.8 | 7.2 |
| CSN2 | casein beta | NM_181008 | 11.9 | 9.8 | 6.2 |
| RGN | regucalcin (senescence marker protein-30) | NM_173957 | 11.5 | 10.0 | 6.5 |
| TLL2 | tolloid-like 2 | XM_002698408 /// XM_864694 | 11.4 | 9.4 | 5.8 |
| GPT | glutamic-pyruvate transaminase (alanine aminotransferase) | NM_001083740 | 11.2 | 10.1 | 6.6 |
| TNPO1 | transportin 1 | NM_001076540 | 11.1 | 9.9 | 6.4 |
| NPR3 | natriuretic peptide receptor C/guanylate cyclase C (atrionatriuretic peptide receptor C | NM_174127 | 10.9 | 6.3 | 2.9 |
| PRR15 | proline rich 15 | NM_001205521 | 10.9 | 9.4 | 6.0 |
| SLC16A3 | solute carrier family 16, member 3 (monocarboxylic acid transporter 4) | NM_001109980 | 10.6 | 8.4 | 5.0 |
| BTBD7 | BTB (POZ) domain containing 7 | XM_002696820 /// XM_003585089 | 10.5 | 7.3 | 3.9 |
| LOC404103 | spleen trypsin inhibitor | NM_205786 | 10.4 | 9.1 | 5.7 |
| FAM78A | family with sequence similarity 78, member A | NM_001038508 | 10.4 | 8.9 | 5.5 |
| NT5E | 5'-nucleotidase, ecto (CD73) | NM_174129 | 10.4 | 8.7 | 5.3 |
| SLCO3A1 | solute carrier organic anion transporter family, member 3A1 | NM_001001134 | 10.0 | 8.3 | 5.0 |
| PIK3R1 | phosphoinositide-3-kinase, regulatory subunit 1 (alpha) | NM_174575 | 9.9 | 8.7 | 5.4 |
| MTR | 5-methyltetrahydrofolate-homocysteine methyltransferase | NM_001030298 | 9.9 | 7.4 | 4.1 |
| ADAM9 | ADAM metallopeptidase domain 9 | NM_001192818 | 9.8 | 8.0 | 4.7 |

| | | | | | |
|---|---|---|---|---|---|
| TMEM120A | transmembrane protein 120A | NM_001079600 | 9.7 | 11.0 | 7.7 |
| LINGO2 | leucine rich repeat and Ig domain containing 2 | XM_002689490 /// XM_590571 | 9.6 | 7.2 | 3.9 |
| FST | follistatin | NM_175801 | 9.5 | 13.5 | 10.3 |
| F2R | coagulation factor II (thrombin) receptor | NM_001103097 | 9.4 | 8.2 | 5.0 |
| ST3GAL4 | ST3 beta-galactoside alpha-2,3-sialyltransferase 4 | NM_205806 | 9.3 | 12.4 | 9.2 |
| STRA6 | stimulated by retinoic acid gene 6 homolog (mouse) | NM_001075730 | 9.3 | 11.3 | 8.0 |
| IDH3A | isocitrate dehydrogenase 3 (NAD+) alpha | NM_174644 | 9.2 | 11.7 | 8.5 |
| PLEKHH1 | pleckstrin homology domain-containing family H member 1-like | XM_003582735 /// XM_003586594 | 9.2 | 8.0 | 4.8 |
| ABCD1 | ATP-binding cassette, sub-family D (ALD), member 1 | NM_001046190 | 9.0 | 8.8 | 5.6 |
| CYBB | cytochrome b-245, beta polypeptide | NM_174035 | 9.0 | 9.3 | 6.1 |
| GDI1 | GDP dissociation inhibitor 1 | NM_174064 | 8.8 | 9.1 | 6.0 |
| FOXO1 | forkhead box O1 | XM_002691748 /// XM_583090 | 8.3 | 10.5 | 7.4 |
| LOC788816 /// TPM1 /// TPM3 | tropomyosin 2, beta-like /// tropomyosin 1 (alpha) /// tropomyosin 3 | NM_001011674 /// NM_001013590 /// XM_001255755 /// XM_002700612 | 8.1 | 12.0 | 9.0 |
| CDK6 | cyclin-dependent kinase 6 | NM_001192301 | 7.9 | 8.2 | 5.3 |
| TMIGD1 | transmembrane and immunoglobulin domain containing 1 | NM_001035036 | 7.9 | 6.5 | 3.5 |
| AFF1 | AF4/FMR2 family, member 1 | NM_001191525 | 7.9 | 8.0 | 5.1 |
| EFHD1 | EF-hand domain family, member D1 | NM_001075832 | 7.9 | 8.5 | 5.5 |
| DDX26B | DEAD/H (Asp-Glu-Ala-Asp/His) box polypeptide 26B | XM_002699587 /// XM_865657 | 7.8 | 8.3 | 5.3 |
| LARP4 /// LOC100848829 | La ribonucleoprotein domain family, member 4 /// la-related protein 4-like | NM_001101189 /// XM_003586056 | 7.6 | 9.4 | 6.5 |
| NBEAL1 | neurobeachin-like 1 | NM_001103173 | 7.5 | 7.6 | 4.7 |
| TET2 | tet methylcytosine dioxygenase 2 | XM_001790146 /// XM_002688092 /// XM_003582308 /// XM_003586185 | 7.4 | 8.3 | 5.4 |

| | | | | | |
|---|---|---|---|---|---|
| GCLC | glutamate-cysteine ligase, catalytic subunit | NM_001083674 | 7.4 | 9.8 | 6.9 |
| ANAPC5 | anaphase promoting complex subunit 5 | NM_001192945 | 7.3 | 9.0 | 6.1 |
| BTBD11 | BTB (POZ) domain containing 11 | NM_001205745 | 7.3 | 7.3 | 4.4 |
| ADNP | activity-dependent neuroprotector homeobox | NM_001206031 | 7.3 | 6.3 | 3.5 |
| OBSL1 | obscurin-like 1 | NM_001075491 | 7.3 | 7.5 | 4.6 |
| ANTXR2 | anthrax toxin receptor 2 | NM_001076826 | 7.3 | 8.3 | 5.4 |
| UPK1B | uroplakin 1B | NM_174482 | 7.2 | 10.1 | 7.3 |
| C21H14orf132 | chromosome 21 open reading frame, human C14orf132 | NM_001252510 /// XM_002696766 /// XM_870197 | 7.1 | 9.4 | 6.5 |
| SEMA6D | sema domain, transmembrane domain (TM), and cytoplasmic domain, (semaphorin) 6D | NM_001191133 | 7.1 | 10.0 | 7.2 |
| FAM188A | family with sequence similarity 188, member A | NM_001076107 | 7.1 | 10.9 | 8.1 |
| SLC38A2 | solute carrier family 38, member 2 | NM_001082424 | 7.0 | 7.7 | 4.9 |
| LOC614107 | hexokinase 2-like | XM_865470 | 7.0 | 7.9 | 5.1 |
| TMEM176B | transmembrane protein 176B | NM_001099145 | 7.0 | 10.6 | 7.8 |
| ACE2 | angiotensin I converting enzyme (peptidyl-dipeptidase A) 2 | NM_001024502 /// NM_001191154 | 7.0 | 9.6 | 6.8 |
| TMEM138 | transmembrane protein 138 | NM_001098967 | 6.9 | 6.7 | 3.9 |
| THBS3 | thrombospondin 3 | NM_001101839 | 6.9 | 8.5 | 5.7 |
| NDRG4 | NDRG family member 4 | NM_001075695 | 6.9 | 7.9 | 5.1 |
| SLAIN1 | SLAIN motif family, member 1 | XM_002691900 /// XM_003582874 /// XM_003582875 /// XM_003586721 /// XM_583891 | 6.9 | 8.5 | 5.7 |
| LOC100850064 | versican core protein-like | XM_003582488 | 6.9 | 11.8 | 9.0 |
| ELL2 | elongation factor, RNA polymerase II, 2 | NM_001191146 | 6.8 | 9.7 | 6.9 |
| PDP2 | pyruvate dehyrogenase phosphatase catalytic subunit 2 | XM_002694806 /// XM_003584879 | 6.8 | 9.9 | 7.2 |
| MT1A | metallothionein-1A | NM_001040492 | 6.7 | 10.9 | 8.1 |

| | | | | | |
|---|---|---|---|---|---|
| PDSS1 | prenyl (decaprenyl) diphosphate synthase, subunit 1 | NM_001100331 | 6.7 | 10.2 | 7.5 |
| MERTK | c-mer proto-oncogene tyrosine kinase | NM_001192024 | 6.6 | 8.8 | 6.0 |
| B3GALT2 | UDP-Gal:betaGlcNAc beta 1,3-galactosyltransferase, polypeptide 2 | NM_001076188 | 6.5 | 7.4 | 4.7 |
| UBE2B | ubiquitin-conjugating enzyme E2B | NM_001037459 | 6.5 | 7.0 | 4.3 |
| CA5B | carbonic anhydrase VB, mitochondrial | NM_001080908 | 6.5 | 7.4 | 4.7 |
| TRIB2 | tribbles homolog 2 (Drosophila) | NM_178317 | 6.5 | 11.1 | 8.4 |
| FAM114A1 | family with sequence similarity 114, member A1 | XM_002688202 /// XM_588946 | 6.4 | 8.6 | 5.9 |
| CRABP2 | cellular retinoic acid binding protein 2 | NM_001008670 | 6.4 | 7.1 | 4.4 |
| MEX3C | mex-3 homolog C (C. elegans) | XM_002697824 /// XM_607763 | 6.4 | 10.7 | 8.0 |
| ADAM10 | ADAM metallopeptidase domain 10 | NM_174496 | 6.3 | 8.6 | 5.9 |
| BCAR3 | breast cancer anti-estrogen resistance 3 | NM_001024483 /// XM_003581980 | 6.3 | 8.9 | 6.2 |
| STBD1 | starch binding domain 1 | XM_002688357 /// XM_591045 | 6.3 | 10.0 | 7.4 |
| POR | P450 (cytochrome) oxidoreductase | NM_001035390 | 6.3 | 10.6 | 7.9 |
| INSIG1 | insulin induced gene 1 | NM_001077909 | 6.3 | 10.4 | 7.8 |
| TMEM156 | transmembrane protein 156 | NM_001083469 | 6.3 | 7.5 | 4.9 |
| RHBDD2 | rhomboid domain containing 2 | NM_001046478 | 6.2 | 8.9 | 6.2 |
| CSDE1 | cold shock domain containing E1, RNA-binding | NM_001098025 | 6.2 | 7.5 | 4.9 |
| SCD | stearoyl-CoA desaturase (delta-9-desaturase) | NM_173959 /// XM_002698490 | 6.2 | 11.7 | 9.0 |
| SH3GL2 | SH3-domain GRB2-like 2 | NM_001076840 | 6.1 | 7.6 | 4.9 |
| RBBP8 | retinoblastoma binding protein 8 | NM_001101966 | 6.1 | 8.4 | 5.8 |
| DOCK5 | dedicator of cytokinesis 5 | XM_002689790 /// XM_581879 | 6.1 | 7.0 | 4.4 |
| GATA6 | GATA binding protein 6 | XM_001253596 /// XM_002697727 | 6.0 | 12.1 | 9.6 |
| PTPRD | protein tyrosine phosphatase, receptor type, D | XM_002689593 /// XM_003582520 /// | 5.8 | 9.1 | 6.6 |

| | | | | | |
|---|---|---|---|---|---|
| | | XM_003582521 /// XM_003582522 /// XM_003582523 /// XM | | | |
| CYCS /// LOC100847304 /// LOC100847700 /// LOC100850794 | cytochrome c, somatic /// cytochrome c-like /// cytochrome c-like /// cytochrome c-like | NM_001046061 /// XM_003582829 /// XM_003582831 /// XM_003583532 /// XM_003586673 /// XM | 5.8 | 9.7 | 7.2 |
| MFGE8 | milk fat globule-EGF factor 8 protein | NM_176610 | 5.8 | 12.3 | 9.8 |
| PTHLH | parathyroid hormone-like hormone | NM_174753 | 5.8 | 7.8 | 5.3 |
| SOAT1 | sterol O-acyltransferase 1 | NM_001034206 | 5.7 | 11.2 | 8.7 |
| CGN | cingulin | NM_001192786 | 5.7 | 8.8 | 6.3 |
| MAL2 | mal, T-cell differentiation protein 2 | NM_001081719 | 5.7 | 9.0 | 6.5 |
| SCYL2 | SCY1-like 2 (S. cerevisiae) | NM_001102529 | 5.7 | 6.5 | 4.0 |
| ENPEP | glutamyl aminopeptidase (aminopeptidase A) | NM_001038027 | 5.7 | 6.6 | 4.1 |
| FYTTD1 | forty-two-three domain containing 1 | NM_001001137 | 5.6 | 7.2 | 4.8 |
| C19H17orf63 | chromosome 19 open reading frame, human C17orf63 | XM_002695749 /// XM_003583543 /// XM_003587387 /// XM_882223 | 5.6 | 7.6 | 5.1 |
| NCEH1 | neutral cholesterol ester hydrolase 1 | NM_001123034 | 5.6 | 7.3 | 4.8 |
| HPSE | heparanase | NM_174082 | 5.6 | 5.9 | 3.4 |
| MPV17L2 | MPV17 mitochondrial membrane protein-like 2 | NM_001098145 | 5.6 | 11.1 | 8.7 |
| RCAN3 | RCAN family member 3 | NM_001045945 | 5.5 | 8.2 | 5.7 |
| PRPF39 | PRP39 pre-mRNA processing factor 39 homolog (S. cerevisiae) | NM_001109789 | 5.5 | 7.2 | 4.7 |
| SLC29A1 | solute carrier family 29 (nucleoside transporters), member 1 | NM_001034398 | 5.5 | 10.9 | 8.4 |
| CCND2 | cyclin D2 | NM_001076372 | 5.5 | 11.4 | 9.0 |
| HEATR1 | HEAT repeat containing 1 | NM_001076448 /// XM_583512 /// XM_585511 | 5.5 | 6.2 | 3.8 |

| | | | | | |
|---|---|---|---|---|---|
| ANKRD1 | ankyrin repeat domain 1 (cardiac muscle) | NM_001034378 | 5.4 | 6.7 | 4.3 |
| F2RL1 | coagulation factor II (thrombin) receptor-like 1 | NM_001046283 | 5.4 | 8.1 | 5.7 |
| IGF1R | insulin-like growth factor 1 receptor | NM_001244612 | 5.4 | 7.8 | 5.4 |
| PKM | pyruvate kinase, muscle | NM_001205727 | 5.4 | 9.3 | 6.9 |
| MYO1B | myosin IB | NM_001102199 | 5.4 | 6.8 | 4.4 |
| F3 /// LOC100849695 | coagulation factor III /// tissue factor-like | NM_173878 /// XM_003581982 | 5.4 | 7.7 | 5.3 |
| CASP3 | caspase 3, apoptosis-related cysteine peptidase | NM_001077840 | 5.3 | 6.0 | 3.5 |
| ID2 | inhibitor of DNA binding 2, dominant negative helix-loop-helix protein | NM_001034231 | 5.3 | 10.7 | 8.3 |
| CTDSPL2 | CTD (carboxy-terminal domain, RNA polymerase II, polypeptide A) small phosphatase like | NM_001191405 | 5.3 | 6.2 | 3.8 |
| PPM1K | protein phosphatase, Mg2+/Mn2+ dependent, 1K | NM_001046474 | 5.3 | 8.6 | 6.2 |
| AMIGO2 | adhesion molecule with Ig-like domain 2 | NM_001205786 | 5.3 | 8.9 | 6.5 |
| GYLTL1B | glycosyltransferase-like 1B | NM_001206192 | 5.3 | 7.8 | 5.4 |
| ROBO2 | roundabout, axon guidance receptor, homolog 2 (Drosophila) | XM_002684652 /// XM_614756 | 5.3 | 6.3 | 3.9 |
| PAPD5 | PAP associated domain containing 5 | XM_001256515 /// XM_002694879 | 5.2 | 9.0 | 6.6 |
| LOC532189 | carboxypeptidase D-like | XM_003583547 /// XM_003587391 /// XM_003587397 | 5.2 | 12.4 | 10.1 |
| CANT1 | calcium activated nucleotidase 1 | XM_002696154 /// XM_596269 | 5.2 | 8.0 | 5.6 |
| FNDC3B | fibronectin type III domain containing 3B | NM_001206211 | 5.2 | 10.8 | 8.4 |
| PPAP2B | phosphatidic acid phosphatase type 2B | NM_001076473 | 5.2 | 11.3 | 8.9 |
| TRIM6 | tripartite motif containing 6 | NM_001205189 | 5.2 | 6.4 | 4.0 |
| ITGA2 | integrin, alpha 2 (CD49B, alpha 2 subunit of VLA-2 receptor) | NM_001166499 | 5.2 | 5.7 | 3.3 |
| RDH11 | retinol dehydrogenase 11 (all-trans/9-cis/11-cis) | XM_002690979 /// XM_582373 | 5.2 | 7.1 | 4.8 |
| PRAF2 | PRA1 domain family, member 2 | NM_001046009 | 5.2 | 9.0 | 6.6 |

| | | | | | |
|---|---|---|---|---|---|
| IPMK | inositol polyphosphate multikinase | NM_001035462 | 5.1 | 6.0 | 3.6 |
| SYNGR2 | synaptogyrin 2 | NM_001100358 | 5.1 | 10.4 | 8.0 |
| SLC25A12 | solute carrier family 25 (mitochondrial carrier, Aralar), member 12 | NM_001101194 | 5.1 | 6.3 | 3.9 |
| PPP1CB | protein phosphatase 1, catalytic subunit, beta isozyme | NM_001034653 | 5.1 | 8.1 | 5.8 |
| RASGEF1B | RasGEF domain family, member 1B | NM_001083649 | 5.1 | 7.6 | 5.3 |
| CCNT1 | cyclin T1 | NM_001001147 | 5.1 | 6.6 | 4.3 |
| PPP1R3F | protein phosphatase 1, regulatory subunit 3F | NM_001193144 | 5.1 | 7.5 | 5.2 |
| TK1 | thymidine kinase 1, soluble | NM_001097572 | 5.1 | 9.2 | 6.9 |
| FLVCR2 | feline leukemia virus subgroup C cellular receptor family, member 2 | NM_001192143 | 5.1 | 7.7 | 5.4 |
| CDH2 | cadherin 2, type 1, N-cadherin (neuronal) | NM_001166492 | 5.0 | 8.7 | 6.4 |
| RNPC3 | RNA-binding region (RNP1, RRM) containing 3 | NM_001077117 | 5.0 | 8.8 | 6.5 |
| MRAP | melanocortin 2 receptor accessory protein | XM_002684584 /// XM_582078 | 5.0 | 6.9 | 4.6 |
| CHSY1 | chondroitin sulfate synthase 1 | NM_001191157 | 5.0 | 5.7 | 3.4 |
| DDIT4 | DNA-damage-inducible transcript 4 | NM_001075922 | 5.0 | 11.8 | 9.5 |
| PFKFB4 | 6-phosphofructo-2-kinase/fructose-2,6-biphosphatase 4 | NM_001192835 | 5.0 | 7.0 | 4.7 |
| ADAM12 | ADAM metallopeptidase domain 12 | NM_001001156 | 5.0 | 7.6 | 5.3 |
| CAPRIN2 | caprin family member 2 | XM_002687720 /// XM_871851 | 4.9 | 11.0 | 8.7 |
| HIATL1 | hippocampus abundant transcript-like 1 | NM_001083662 | 4.9 | 7.0 | 4.7 |
| SLC2A3 | solute carrier family 2 (facilitated glucose transporter), member 3 | NM_174603 | 4.9 | 6.0 | 3.7 |
| PBRM1 | polybromo 1 | XM_002697017 /// XM_003587634 /// XM_583018 | 4.9 | 8.4 | 6.1 |
| BMPR2 | bone morphogenetic protein receptor, type II (serine/threonine kinase) | XM_002685492 /// XM_617592 | 4.9 | 5.7 | 3.4 |
| AHCYL2 | adenosylhomocysteinase-like 2 | NM_001101143 | 4.9 | 8.6 | 6.4 |
| ACBD5 | acyl-CoA binding domain containing 5 | NM_181038 | 4.9 | 7.1 | 4.9 |

| | | | | | | |
|---|---|---|---|---|---|---|
| PPARG | peroxisome proliferator-activated receptor gamma | NM_181024 | 4.8 | 8.2 | 5.9 | |
| RAVER2 | ribonucleoprotein, PTB-binding 2 | XM_002686338 /// XM_600332 | 4.8 | 6.9 | 4.6 | |
| FUBP1 | far upstream element (FUSE) binding protein 1 | NM_001076846 | 4.8 | 6.7 | 4.4 | |
| MOCS1 | molybdenum cofactor synthesis 1 | NM_001013597 /// NM_001166297 | 4.8 | 8.8 | 6.5 | |
| ZBTB33 | zinc finger and BTB domain containing 33 | NM_001098157 | 4.8 | 6.9 | 4.6 | |
| FAM184A | family with sequence similarity 184, member A | XM_002690079 /// XM_002705132 /// XM_614661 | 4.8 | 7.1 | 4.8 | |
| INHBB | inhibin, beta B | NM_176852 | 4.8 | 6.5 | 4.3 | |
| FUT1 | fucosyltransferase 1 (galactoside 2-alpha-L-fucosyltransferase, H blood group) | NM_177499 | 4.8 | 6.6 | 4.3 | |
| VEGFA | --- | --- | | 10.0 | 7.8 | 4.7 |
| CLPTM1 | cleft lip and palate associated transmembrane protein 1 | NM_001046613 | 4.7 | 8.6 | 6.4 | |
| ECE1 | endothelin converting enzyme 1 | NM_181009 | 4.7 | 9.8 | 7.5 | |
| ROR2 | receptor tyrosine kinase-like orphan receptor 2 | NM_001105464 | 4.7 | 9.6 | 7.3 | |
| COPA | coatomer protein complex, subunit alpha | NM_001105645 | 4.7 | 5.6 | 3.4 | |
| LOC100847279 /// STAM2 | signal transducing adapter molecule 2-like /// signal transducing adaptor molecule (SH3 | NM_001076106 /// XM_003585736 | 4.7 | 8.1 | 5.8 | |
| TIA1 | TIA1 cytotoxic granule-associated RNA binding protein | NM_001076109 | 4.7 | 7.8 | 5.6 | |
| RPAP3 | RNA polymerase II associated protein 3 | NM_001101930 /// XM_002687317 /// XM_002704277 | 4.7 | 9.8 | 7.6 | |
| ZFPM1 | zinc finger protein, multitype 1 | XM_002694855 /// XM_606758 | 4.7 | 7.2 | 5.0 | |
| PIP4K2A | phosphatidylinositol-5-phosphate 4-kinase, type II, alpha | NM_001192769 | 4.7 | 6.0 | 3.8 | |
| USP28 | ubiquitin specific peptidase 28 | NM_001192998 | 4.6 | 6.1 | 3.8 | |
| CHAC1 | ChaC, cation transport regulator homolog 1 (E. coli) | NM_001098882 | 4.6 | 7.3 | 5.1 | |
| CCDC134 | coiled-coil domain containing 134 | NM_001205826 | 4.6 | 8.6 | 6.4 | |
| LOC509420 | chromosome 9 open reading frame 61-like | XM_003582533 /// XM_003586391 | 4.6 | 6.5 | 4.3 | |

| | | | | | |
|---|---|---|---|---|---|
| RAP2A | RAP2A, member of RAS oncogene family | NM_001192942 | 4.6 | 6.1 | 3.9 |
| MTFR1 | mitochondrial fission regulator 1 | NM_001035412 | 4.6 | 7.0 | 4.8 |
| ELOVL6 | ELOVL fatty acid elongase 6 | NM_001102155 | 4.6 | 6.7 | 4.5 |
| KCTD20 | potassium channel tetramerisation domain containing 20 | NM_001101093 | 4.6 | 5.5 | 3.4 |
| HMGCS1 | HMGCS1 protein-like | NM_001206578 | 4.5 | 11.7 | 9.6 |
| CHST11 | carbohydrate (chondroitin 4) sulfotransferase 11 | NM_001192668 | 4.5 | 6.0 | 3.8 |
| SLC25A13 | solute carrier family 25, member 13 (citrin) | NM_001193076 /// XM_002686663 /// XM_003582054 | 4.5 | 6.8 | 4.6 |
| RRAD | Ras-related associated with diabetes | NM_001045913 | 4.5 | 9.2 | 7.0 |
| KLK1 | kallikrein 1 | NM_001008416 | 4.5 | 7.4 | 5.2 |
| CHN2 | chimerin (chimaerin) 2 | NM_001045963 | 4.5 | 7.8 | 5.7 |
| EIF4EBP1 | eukaryotic translation initiation factor 4E binding protein 1 | NM_001077893 | 4.5 | 10.6 | 8.4 |
| TOB1 | transducer of ERBB2, 1 | NM_001077075 /// XM_001252475 | 4.5 | 10.7 | 8.5 |
| SRGN | serglycin | NM_001025326 | 4.5 | 12.4 | 10.2 |
| SLITRK2 | SLIT and NTRK-like family, member 2 | XM_002699621 /// XM_591589 | 4.5 | 8.6 | 6.5 |
| CAMK1 | calcium/calmodulin-dependent protein kinase I | NM_001076868 | 4.5 | 7.1 | 4.9 |
| LNP1 | leukemia NUP98 fusion partner 1 | NM_001205535 | 4.5 | 6.2 | 4.0 |
| SLC38A1 | solute carrier family 38, member 1 | XM_001790621 /// XM_002687321 | 4.5 | 5.4 | 3.2 |
| GNG10 | guanine nucleotide binding protein (G protein), gamma 10 | NM_001114512 | 4.4 | 12.1 | 9.9 |
| MOSPD3 | motile sperm domain containing 3 | NM_001034265 | 4.4 | 9.5 | 7.3 |
| S100PBP | S100P binding protein | NM_001034687 | 4.4 | 7.4 | 5.2 |
| LOC100850047 /// ZNF609 | zinc finger protein 609-like /// zinc finger protein 609 | NM_001193023 /// XM_003585405 | 4.4 | 8.0 | 5.9 |
| PLD1 | phospholipase D1, phosphatidylcholine-specific | NM_001102001 | 4.4 | 5.7 | 3.5 |
| JAK3 | Janus kinase 3 | XM_002688539 /// XM_002704713 | 4.4 | 10.9 | 8.7 |
| LOC100850098 | 28S ribosomal protein S6, mitochondrial-like /// | NM_001040584 /// | 4.4 | 11.5 | 9.3 |

| | | | | | | |
|---|---|---|---|---|---|---|
| /// MRPS6 | mitochondrial ribosomal protein S6 | XM_003584706 | | | | |
| SMAD2 /// SMAD3 | SMAD family member 2 /// SMAD family member 3 | NM_001046218 /// NM_001205805 | 4.4 | 10.6 | 8.5 | |
| SLC25A28 | solute carrier family 25, member 28 | NM_001205552 | 4.4 | 11.0 | 8.9 | |
| SLC25A39 | solute carrier family 25, member 39 | NM_001075415 | 4.4 | 9.3 | 7.1 | |
| LOC100850907 /// MAPK6 | mitogen-activated protein kinase 6-like /// mitogen-activated protein kinase 6 | XM_002690965 /// XM_002706554 /// XM_003585063 | 4.3 | 6.4 | 4.3 | |
| MYO9A | myosin IXA | XM_002690529 /// XM_593333 | 4.3 | 6.8 | 4.6 | |
| INHA | inhibin, alpha | NM_174094 | 4.3 | 13.6 | 11.5 | |
| C5H12orf35 | chromosome 5 open reading frame, human C12orf35 | NM_001101072 | 4.3 | 8.7 | 6.6 | |
| NUP85 | nucleoporin 85kDa | NM_001034387 | 4.3 | 7.7 | 5.6 | |
| PLA2G1B | phospholipase A2, group IB (pancreas) | NM_174646 | 4.3 | 9.1 | 7.0 | |
| MORN4 | MORN repeat containing 4 | NM_001035450 | 4.3 | 7.9 | 5.8 | |
| HECTD1 | HECT domain containing E3 ubiquitin protein ligase 1 | XM_002696696 /// XM_003583752 /// XM_872366 | 4.3 | 6.7 | 4.6 | |
| KIAA0564 | --- | --- | | 6.9 | 4.8 | 4.2 |
| ENTPD4 | ectonucleoside triphosphate diphosphohydrolase 4 | NM_001077985 | 4.2 | 7.7 | 5.6 | |
| MZT1 | mitotic spindle organizing protein 1 | NM_001206331 | 4.2 | 8.8 | 6.7 | |
| ALG3 | asparagine-linked glycosylation 3, alpha-1,3-mannosyltransferase homolog (S. cerevisia | NM_001083511 | 4.2 | 6.0 | 4.0 | |
| ME3 | malic enzyme 3, NADP(+)-dependent, mitochondrial | NM_001075877 | 4.2 | 7.4 | 5.4 | |
| GPX2 | glutathione peroxidase 2 (gastrointestinal) | NM_001163139 | 4.2 | 6.5 | 4.4 | |
| AMH | anti-Mullerian hormone | NM_173890 | 4.2 | 9.2 | 7.1 | |
| ANKRD6 | ankyrin repeat domain 6 | NM_001075736 /// NM_001205477 | 4.2 | 7.1 | 5.0 | |
| PIGS | phosphatidylinositol glycan anchor biosynthesis, class S | NM_001034216 | 4.2 | 7.1 | 5.0 | |
| CSPG4 | chondroitin sulfate proteoglycan 4 | NM_001192782 | 4.2 | 8.3 | 6.2 | |
| BoLA | major histocompatibility complex, class I, A | NM_001114855 | 4.2 | 6.9 | 4.8 | |

| | | | | | |
|---|---|---|---|---|---|
| TFRC | transferrin receptor (p90, CD71) | NM_001206577 | 4.2 | 6.0 | 4.0 |
| LOC404051 | serpin peptidase inhibitor, clade B (ovalbumin), member 6-like | XM_866025 | 4.2 | 5.5 | 3.5 |
| NUDT10 | nudix (nucleoside diphosphate linked moiety X)-type motif 10 | NM_001035488 | 4.2 | 7.0 | 4.9 |
| LOC783497 /// PPP1R14C | protein phosphatase 1, regulatory (inhibitor) subunit 14C-like /// protein phosphatase | NM_001078068 /// XM_002692947 /// XM_003583080 | 4.2 | 6.3 | 4.3 |
| LHCGR | luteinizing hormone/choriogonadotropin receptor | NM_174381 | 4.1 | 8.0 | 5.9 |
| RGNEF | 190 kDa guanine nucleotide exchange factor | NM_001102307 | 4.1 | 10.5 | 8.5 |
| ATP6V1A | ATPase, H+ transporting, lysosomal 70kDa, V1 subunit A | NM_174504 | 4.1 | 9.0 | 7.0 |
| NEU3 | sialidase 3 (membrane sialidase) | NM_174122 | 4.1 | 5.4 | 3.4 |
| HOOK1 | hook homolog 1 (Drosophila) | NM_001206870 /// XM_003582004 | 4.1 | 6.4 | 4.3 |
| KRAS | v-Ki-ras2 Kirsten rat sarcoma viral oncogene homolog | NM_001110001 | 4.1 | 10.1 | 8.1 |
| NTRK1 | neurotrophic tyrosine kinase, receptor, type 1 | XM_002685965 /// XM_002685966 /// XM_613650 /// XM_882395 | 4.1 | 6.7 | 4.6 |
| HSPH1 | heat shock 105kDa/110kDa protein 1 | NM_001075302 | 4.1 | 5.1 | 3.0 |
| QSER1 | glutamine and serine rich 1 | NM_001256881 /// XM_002693518 /// XM_003585105 /// XM_617172 /// XM_865223 | 4.1 | 6.1 | 4.0 |
| WBP4 | WW domain binding protein 4 (formin binding protein 21) | NM_001075251 | 4.1 | 6.6 | 4.5 |
| ITGAV | integrin, alpha V (vitronectin receptor, alpha polypeptide, antigen CD51) | NM_174367 | 4.1 | 8.4 | 6.4 |
| FAM13A | family with sequence similarity 13, member A | NM_174692 /// XM_003582315 | 4.1 | 5.5 | 3.5 |
| ATAD1 | ATPase family, AAA domain containing 1 | NM_001205581 | 4.0 | 7.0 | 5.0 |
| KLC2 | kinesin light chain 2 | NM_001075768 | 4.0 | 6.6 | 4.6 |
| AGPAT5 | 1-acylglycerol-3-phosphate O-acyltransferase 5 | NM_001075932 | 4.0 | 8.8 | 6.8 |

| | | | | | |
|---|---|---|---|---|---|
| | (lysophosphatidic acid acyltransferase, | | | | |
| LOC505766 | BMP-2-inducible protein kinase-like | XM_003582341 /// XM_003586219 | 4.0 | 7.5 | 5.5 |
| Mar-06 | membrane-associated ring finger (C3HC4) 6 | NM_001205812 | 4.0 | 11.0 | 9.0 |
| ABCC9 | ATP-binding cassette, sub-family C (CFTR/MRP), member 9 | XM_002687737 /// XM_003582250 | -4.0 | 4.9 | 6.9 |
| WARS2 | tryptophanyl tRNA synthetase 2, mitochondrial | -4.0102 | -4.0 | 6.7 | 8.7 |
| LOC100848191 | latent-transforming growth factor beta-binding protein 4-like | XM_003587266 /// XR_138945 | -4.0 | 5.0 | 7.0 |
| FAM117A | family with sequence similarity 117, member A | NM_001101070 | -4.0 | 4.4 | 6.4 |
| IL32 | interleukin 32 | XM_002697939 /// XM_002703050 | -4.0 | 6.2 | 8.2 |
| EGFL7 | EGF-like-domain, multiple 7 | NM_001078038 | -4.0 | 5.3 | 7.3 |
| RND3 | Rho family GTPase 3 | NM_001191158 /// XM_003585297 | -4.0 | 7.0 | 9.1 |
| ADCY8 | adenylate cyclase 8 (brain) | NM_001192841 | -4.0 | 3.7 | 5.7 |
| ABLIM1 | actin binding LIM protein 1 | XM_002698530 /// XM_866358 | -4.0 | 6.4 | 8.4 |
| DTX1 | deltex homolog 1 (Drosophila) | NM_001205734 | -4.1 | 4.6 | 6.6 |
| OOEP | oocyte expressed protein homolog (dog) | NM_001077869 | -4.1 | 5.0 | 7.1 |
| CPQ | plasma glutamate carboxypeptidase | NM_001076248 | -4.1 | 5.8 | 7.8 |
| IL17D | interleukin 17D | XM_002691866 /// XM_866648 | -4.1 | 5.1 | 7.2 |
| CRYM | crystallin, mu | NM_001045914 | -4.1 | 4.5 | 6.5 |
| RBMS2 | RNA binding motif, single stranded interacting protein 2 | NM_001034365 | -4.1 | 6.2 | 8.2 |
| DOPEY2 | dopey family member 2 | XM_002685151 /// XM_865857 | -4.1 | 3.8 | 5.9 |
| UBE2L6 | ubiquitin-conjugating enzyme E2L 6 | NM_001098917 | -4.1 | 5.1 | 7.1 |
| ETS1 | v-ets erythroblastosis virus E26 oncogene homolog 1 (avian) | NM_001099106 | -4.1 | 4.2 | 6.2 |
| CDC42BPG | CDC42 binding protein kinase gamma (DMPK-like) | NM_001102502 | -4.1 | 5.0 | 7.1 |
| BOLA-DQA1 | major histocompatibility complex, class II, DQ alpha, type 1 | NM_001013601 | -4.1 | 4.3 | 6.4 |
| LTBP2 | latent transforming growth factor beta binding protein 2 | NM_174385 | -4.1 | 4.0 | 6.0 |

| | | | | | |
|---|---|---|---|---|---|
| SNTG2 | syntrophin, gamma 2 | XM_002683947 /// XM_867552 | -4.1 | 4.0 | 6.0 |
| GPC3 | glypican 3 | NM_001035463 | -4.1 | 7.5 | 9.6 |
| ENOX1 | ecto-NOX disulfide-thiol exchanger 1 | XM_002691825 /// XM_867914 | -4.1 | 4.3 | 6.3 |
| RASIP1 | Ras interacting protein 1 | NM_001193160 | -4.1 | 5.8 | 7.9 |
| CCDC138 | coiled-coil domain containing 138 | XM_002691278 /// XM_584296 | -4.1 | 5.2 | 7.3 |
| TPCN1 | two pore segment channel 1 | XM_002694517 /// XM_588037 | -4.1 | 4.6 | 6.6 |
| TIMP1 | TIMP metallopeptidase inhibitor 1 | NM_174471 | -4.1 | 8.4 | 10.5 |
| CGREF1 | cell growth regulator with EF-hand domain 1 | NM_001045977 | -4.1 | 4.1 | 6.1 |
| POSTN | periostin, osteoblast specific factor | NM_001040479 | -4.2 | 4.1 | 6.1 |
| PKIG | protein kinase (cAMP-dependent, catalytic) inhibitor gamma | NM_205812 | -4.2 | 5.7 | 7.7 |
| ITPKA | inositol-trisphosphate 3-kinase A | NM_001083519 | -4.2 | 6.0 | 8.1 |
| NGF | nerve growth factor (beta polypeptide) | NM_001099362 | -4.2 | 4.1 | 6.2 |
| NAGK | N-acetylglucosamine kinase | NM_001034314 | -4.2 | 5.6 | 7.6 |
| KANK3 | KN motif and ankyrin repeat domains 3 | NM_001113763 | -4.2 | 5.2 | 7.3 |
| NFKBIA | nuclear factor of kappa light polypeptide gene enhancer in B-cells inhibitor, alpha | NM_001045868 | -4.2 | 6.6 | 8.7 |
| MYADM | myeloid-associated differentiation marker | NM_001075252 | -4.2 | 7.7 | 9.8 |
| MXRA8 | matrix-remodelling associated 8 | NM_001075830 | -4.2 | 7.4 | 9.5 |
| PKP4 | plakophilin 4 | NM_001191491 | -4.2 | 6.0 | 8.1 |
| FAP | fibroblast activation protein, alpha | NM_001098001 | -4.2 | 3.5 | 5.6 |
| NKD2 | naked cuticle homolog 2 (Drosophila) | XM_001256156 /// XM_002696468 | -4.2 | 5.1 | 7.2 |
| FKBP10 | FK506 binding protein 10, 65 kDa | NM_001046403 | -4.2 | 6.9 | 8.9 |
| WDR17 | WD repeat domain 17 | XM_001789371 /// XM_002698642 /// XM_002698643 | -4.2 | 4.1 | 6.2 |
| RGS3 | regulator of G-protein signaling 3 | NM_001077973 | -4.2 | 6.5 | 8.6 |

| | | | | | |
|---|---|---|---|---|---|
| ACVRL1 | activin A receptor type II-like 1 | NM_001083479 | -4.2 | 5.1 | 7.2 |
| ELMO1 | engulfment and cell motility 1 | NM_001024505 /// NM_001113227 | -4.2 | 4.9 | 7.0 |
| PFKP | phosphofructokinase, platelet | NM_001193220 | -4.2 | 5.3 | 7.4 |
| APBB2 | amyloid beta (A4) precursor protein-binding, family B, member 2 | NM_001076847 | -4.2 | 4.9 | 7.0 |
| OSMR | oncostatin M receptor | NM_001080272 /// XM_003583702 /// XM_003587530 | -4.2 | 3.8 | 5.9 |
| MSN | moesin | NM_001046477 | -4.2 | 8.0 | 10.1 |
| PRKCB | protein kinase C, beta | NM_174587 | -4.2 | 5.2 | 7.3 |
| ADCK3 | aarF domain containing kinase 3 | NM_001046419 | -4.2 | 5.9 | 8.0 |
| PTX3 | pentraxin 3, long | NM_001076259 | -4.3 | 4.5 | 6.6 |
| LFNG | LFNG O-fucosylpeptide 3-beta-N-acetylglucosaminyltransferase | NM_001046222 | -4.3 | 5.4 | 7.4 |
| TSPAN7 | tetraspanin 7 | NM_001076384 | -4.3 | 4.6 | 6.7 |
| AIF1 | allograft inflammatory factor 1 | NM_173985 | -4.3 | 5.1 | 7.2 |
| MAP7D1 | MAP7 domain containing 1 | XM_002686551 /// XM_589552 | -4.3 | 6.7 | 8.8 |
| DGKI | diacylglycerol kinase, iota | XM_002687061 /// XM_608560 | -4.3 | 4.4 | 6.5 |
| COLEC11 | collectin sub-family member 11 | NM_001076303 | -4.3 | 5.1 | 7.2 |
| DZIP1 | DAZ interacting protein 1 | NM_001110066 | -4.3 | 6.9 | 9.0 |
| RNF130 | ring finger protein 130 | NM_001099708 | -4.3 | 7.0 | 9.1 |
| CATHL5 | cathelicidin 5 | NM_174510 | -4.3 | 4.2 | 6.3 |
| SCARF1 | scavenger receptor class F, member 1 | XM_002695682 /// XM_002702111 | -4.3 | 4.9 | 7.0 |
| MEGF9 | multiple epidermal growth factor-like domains protein 9-like | XM_003582585 /// XM_003586445 | -4.3 | 4.6 | 6.7 |
| MPEG1 | macrophage expressed 1 | NM_001046464 | -4.3 | 4.9 | 7.0 |
| SCIN | scinderin | NM_174177 | -4.3 | 4.3 | 6.5 |
| REN | renin | NM_001206509 | -4.3 | 4.1 | 6.2 |

| | | | | | |
|---|---|---|---|---|---|
| CD74 | CD74 molecule, major histocompatibility complex, class II invariant chain | NM_001034735 /// NM_174799 | -4.3 | 6.1 | 8.2 |
| ABCC3 | ATP-binding cassette, sub-family C (CFTR/MRP), member 3 | NM_001192756 | -4.3 | 4.5 | 6.6 |
| IPO11 | importin 11 | NM_001192932 | -4.4 | 7.1 | 9.2 |
| RASA3 | RAS p21 protein activator 3 | NM_174676 | -4.4 | 4.8 | 6.9 |
| ZEB1 | zinc finger E-box binding homeobox 1 | NM_001206590 | -4.4 | 5.7 | 7.8 |
| TTC7A | tetratricopeptide repeat domain 7A | NM_001205500 | -4.4 | 4.1 | 6.2 |
| TRPC6 | transient receptor potential cation channel, subfamily C, member 6 | NM_001166572 | -4.4 | 3.6 | 5.7 |
| C10H5orf13 | chromosome 10 open reading frame, human C5orf13 | NM_001105045 | -4.4 | 9.0 | 11.1 |
| KANK2 | KN motif and ankyrin repeat domains 2 | NM_001076531 | -4.4 | 7.0 | 9.2 |
| CYBA | cytochrome b-245, alpha polypeptide | NM_174034 | -4.4 | 5.8 | 7.9 |
| CDH13 | cadherin 13, H-cadherin (heart) | NM_001035277 | -4.4 | 4.4 | 6.5 |
| TNFRSF25 | tumor necrosis factor receptor superfamily, member 25 | NM_001144077 | -4.4 | 3.8 | 6.0 |
| SMIM10 | small integral membrane protein 10 | NM_001163442 | -4.4 | 4.6 | 6.7 |
| FMO5 | flavin containing monooxygenase 5 | NM_001101304 | -4.4 | 4.5 | 6.6 |
| LOC512271 | protein tweety homolog 3-like | XM_003584053 /// XM_003587848 | -4.4 | 7.5 | 9.6 |
| IRF8 | interferon regulatory factor 8 | NM_001083769 | -4.4 | 5.3 | 7.4 |
| CYYR1 | cysteine/tyrosine-rich 1 | NM_001078105 | -4.4 | 4.1 | 6.2 |
| SLC43A1 | solute carrier family 43, member 1 | NM_001206598 | -4.4 | 5.9 | 8.0 |
| GBP5 | guanylate binding protein 5 | NM_001075746 | -4.4 | 3.8 | 6.0 |
| GAS6 | growth arrest-specific 6 | XM_002692001 /// XM_580667 | -4.4 | 5.6 | 7.7 |
| ZNF608 | zinc finger protein 608 | NM_001206306 | -4.4 | 5.5 | 7.6 |
| LOC530341 | cortactin-binding protein 2-like | XR_082754 /// XR_083652 | -4.4 | 4.3 | 6.5 |
| NEB | nebulin | XM_002685358 /// XM_613028 | -4.5 | 3.7 | 5.8 |
| KIAA1522 | KIAA1522 ortholog | XM_001790114 /// XM_002685609 /// | -4.5 | 7.1 | 9.3 |

| | | | | | |
|---|---|---|---|---|---|
| | | XM_003581882 /// XM_003585785 | | | |
| EOGT | chromosome 22 open reading frame, human C3orf64 | NM_001077882 | -4.5 | 5.7 | 7.9 |
| TCF7L1 | transcription factor 7-like 1 (T-cell specific, HMG-box) | XM_002691408 /// XM_593301 | -4.5 | 4.5 | 6.6 |
| BOLA-DMA | major histocompatibility complex, class II, DM alpha | NM_001012674 | -4.5 | 5.2 | 7.3 |
| C1R | complement component 1, r subcomponent | NM_001034407 | -4.5 | 5.6 | 7.8 |
| GSN | gelsolin | NM_001034627 /// NM_001113284 | -4.5 | 8.9 | 11.0 |
| NALCN | sodium leak channel, non-selective | XM_002691980 /// XM_616223 | -4.5 | 5.7 | 7.9 |
| GAS7 | growth arrest-specific 7 | NM_001102280 | -4.5 | 4.0 | 6.1 |
| C16H1orf21 | chromosome 16 open reading frame, human C1orf21 | NM_001081547 | -4.5 | 6.4 | 8.6 |
| MARCKS | myristoylated alanine-rich protein kinase C substrate | NM_001076276 | -4.5 | 8.4 | 10.5 |
| ROBO1 | roundabout, axon guidance receptor, homolog 1 (Drosophila) | NM_001192888 | -4.5 | 6.7 | 8.8 |
| SGK1 | serum/glucocorticoid regulated kinase 1 | NM_001102033 | -4.6 | 4.8 | 7.0 |
| C28H10orf10 | chromosome 28 open reading frame, human C10orf10 | NM_001046515 | -4.6 | 5.0 | 7.2 |
| INPP5F | inositol polyphosphate-5-phosphatase F | XM_003584127 /// XM_003587902 | -4.6 | 5.9 | 8.1 |
| EHBP1L1 | EH domain binding protein 1-like 1 | NM_001191243 | -4.6 | 3.9 | 6.1 |
| MYO18A | myosin XVIIIA | XM_002695670 /// XM_003583544 /// XM_003587388 /// XM_597858 | -4.6 | 4.9 | 7.1 |
| PPAP2A | phosphatidic acid phosphatase type 2A | NM_001080329 | -4.6 | 6.4 | 8.6 |
| CD53 | CD53 molecule | NM_001034232 | -4.6 | 4.1 | 6.3 |
| INPP5D | inositol polyphosphate-5-phosphatase, 145kDa | NM_001101882 | -4.6 | 4.4 | 6.6 |
| SFRP1 | secreted frizzled-related protein 1 | NM_174460 | -4.6 | 5.1 | 7.3 |
| PTPRR | protein tyrosine phosphatase, receptor type, R | NM_001015662 /// NM_001113261 | -4.6 | 4.6 | 6.8 |
| WTIP | Wilms tumor 1 interacting protein | NM_001205562 | -4.6 | 5.6 | 7.9 |

| | | | | | | |
|---|---|---|---|---|---|---|
| RGS10 | regulator of G-protein signaling 10 | NM_001046540 | -4.6 | 5.2 | 7.4 | |
| BT.106027 /// LOC783653 | --- /// --- | --- | | 4.0 | 6.2 | -4.6 |
| FES | feline sarcoma oncogene | NM_001032300 | -4.7 | 4.4 | 6.6 | |
| PRND | prion protein 2 (dublet) | NM_174158 | -4.7 | 4.2 | 6.5 | |
| NRBP2 | nuclear receptor binding protein 2 | NM_001077848 | -4.7 | 7.1 | 9.3 | |
| THBD | thrombomodulin | NM_001166522 | -4.7 | 4.7 | 7.0 | |
| AOX1 | aldehyde oxidase 1 | NM_176668 | -4.7 | 7.6 | 9.8 | |
| C10H15orf48 | chromosome 10 open reading frame, human C15orf48 | NM_001206976 | -4.7 | 4.7 | 6.9 | |
| LOC782991 /// SIRPA | tyrosine-protein phosphatase non-receptor type substrate 1-like /// signal-regulatory p | NM_175788 /// XR_083446 | -4.7 | 4.4 | 6.6 | |
| PLAU | plasminogen activator, urokinase | NM_174147 | -4.7 | 4.8 | 7.0 | |
| RASL11B | RAS-like, family 11, member B | NM_001015635 | -4.7 | 4.7 | 6.9 | |
| ESAM | endothelial cell adhesion molecule | NM_001078066 | -4.7 | 4.4 | 6.7 | |
| VAT1 | vesicle amine transport protein 1 homolog (T. californica) | NM_001192265 | -4.7 | 7.5 | 9.7 | |
| LOC785805 | collagen alpha-5(VI) chain-like | XM_003581780 /// XM_003585711 | -4.7 | 3.6 | 5.9 | |
| CCL16 | chemokine (C-C motif) ligand 16 | XM_002695627 /// XM_868834 | -4.7 | 3.5 | 5.7 | |
| METTL7A /// METTL7A | methyltransferase like 7A-like /// methyltransferase like 7A | NM_001035439 /// XM_001249762 | -4.7 | 5.0 | 7.2 | |
| LOC100337074 | Leukocyte elastase inhibitor-like | XM_002697631 | -4.8 | 7.2 | 9.4 | |
| IL1R1 | interleukin 1 receptor, type I | NM_001206735 | -4.8 | 4.1 | 6.4 | |
| NFIA | nuclear factor I/A | NM_001038209 | -4.8 | 7.3 | 9.6 | |
| FRY | furry homolog (Drosophila) | NM_001205616 | -4.8 | 5.6 | 7.8 | |
| CDH11 /// LOC100851861 | cadherin 11, type 2, OB-cadherin (osteoblast) /// cadherin-11-like | NM_001081624 /// XR_138938 | -4.8 | 7.9 | 10.2 | |
| CTSK | cathepsin K | NM_001034435 | -4.8 | 7.8 | 10.1 | |
| AFAP1L1 | actin filament associated protein 1-like 1 | NM_001100334 | -4.8 | 4.3 | 6.6 | |
| RGS19 | regulator of G-protein signaling 19 | NM_001076915 | -4.8 | 4.7 | 6.9 | |
| RAB3D | RAB3D, member RAS oncogene family | NM_001191375 | -4.8 | 5.9 | 8.2 | |

| | | | | | |
|---|---|---|---|---|---|
| COL4A5 | collagen, type IV, alpha 5 | XM_001790133 /// XM_001790134 /// XM_002699860 /// XM_002699862 | -4.8 | 6.1 | 8.4 |
| ITGA8 | integrin, alpha 8 | XM_002692081 /// XM_002701040 | -4.8 | 3.1 | 5.4 |
| GUCY1B3 | guanylate cyclase 1, soluble, beta 3 | NM_174641 | -4.8 | 8.1 | 10.4 |
| GAMT | guanidinoacetate N-methyltransferase | NM_001038544 | -4.8 | 6.5 | 8.8 |
| ADCY4 | adenylate cyclase 4 | NM_001099208 | -4.9 | 4.7 | 7.0 |
| POLD4 | polymerase (DNA-directed), delta 4 | NM_001076481 | -4.9 | 4.9 | 7.2 |
| FMNL3 | formin-like 3 | NM_001191506 | -4.9 | 5.1 | 7.4 |
| S1PR2 | sphingosine-1-phosphate receptor 2 | NM_001081541 | -4.9 | 6.0 | 8.3 |
| CXCL11 | chemokine (C-X-C motif) ligand 11 | NM_001113173 | -4.9 | 3.6 | 5.8 |
| COL13A1 | collagen, type XIII, alpha 1 | NM_001105433 | -4.9 | 4.4 | 6.7 |
| CDO1 | cysteine dioxygenase, type I | NM_001034465 | -4.9 | 7.1 | 9.4 |
| FAM109B | family with sequence similarity 109, member B | NM_001045936 | -4.9 | 5.3 | 7.6 |
| MAP2 | microtubule-associated protein 2 | NM_001205807 | -4.9 | 4.9 | 7.2 |
| FAM105A | family with sequence similarity 105, member A | NM_001102171 | -4.9 | 7.0 | 9.3 |
| KIAA1598 /// LOC100848050 | KIAA1598 ortholog /// uncharacterized LOC100848050 | NM_001110080 /// XM_003584108 /// XM_003584109 /// XM_003587898 /// XM_003587899 /// XR | -4.9 | 5.7 | 8.0 |
| ARHGAP24 | Rho GTPase activating protein 24 | NM_001102234 | -4.9 | 4.5 | 6.8 |
| LOC510193 /// LOC527460 | apolipoprotein L, 3-like /// apolipoprotein L, 3-like | NM_001100333 /// XM_002687683 /// XM_605852 | -4.9 | 3.8 | 6.1 |
| C5H12orf75 | chromosome 5 open reading frame, human C12orf75 | NM_001145202 | -4.9 | 5.2 | 7.5 |
| EFEMP1 | EGF containing fibulin-like extracellular matrix protein 1 | NM_001081717 | -5.0 | 4.0 | 6.3 |
| SSBP2 | single-stranded DNA binding protein 2 | NM_001035478 | -5.0 | 6.4 | 8.8 |
| TMEM243 | chromosome 4 open reading frame, human C7orf23 | NM_001078044 | -5.0 | 5.3 | 7.6 |

| | | | | | | |
|---|---|---|---|---|---|---|
| SMTN | smoothelin | NM_001076879 | -5.0 | 5.3 | 7.6 | |
| PHGDH | phosphoglycerate dehydrogenase | NM_001035017 | -5.0 | 6.7 | 9.0 | |
| MECOM | MDS1 and EVI1 complex locus | XM_002684943 /// XM_003581739 /// XM_003581740 /// XM_003581741 /// XM_003581742 /// XM | -5.0 | 4.4 | 6.7 | |
| GCOM1 | --- | --- | | 5.3 | 7.6 | -5.0 |
| CNN2 | calponin 2 | NM_001035420 | -5.0 | 5.7 | 8.1 | |
| CD14 | CD14 molecule | NM_174008 | -5.0 | 4.1 | 6.5 | |
| LOC100337091 | 1-phosphatidylinositol-4,5-bisphosphate phosphodiesterase gamma-2-like | XM_003583364 /// XM_003585201 /// XM_003587203 | -5.0 | 4.3 | 6.7 | |
| PDPN | podoplanin | NM_001033120 | -5.1 | 5.7 | 8.1 | |
| SLC35D2 | solute carrier family 35, member D2 | NM_001206266 | -5.1 | 4.9 | 7.2 | |
| APLNR | apelin receptor | NM_001102524 | -5.1 | 4.6 | 7.0 | |
| FBLN2 | fibulin 2 | XM_002697131 /// XM_589271 | -5.1 | 4.5 | 6.8 | |
| LGMN | legumain | NM_174101 /// XM_003585053 | -5.1 | 7.3 | 9.6 | |
| CST6 | cystatin E/M | NM_001012764 | -5.1 | 5.5 | 7.8 | |
| LOC520070 | endosialin-like | XM_003584268 /// XM_003588045 | -5.1 | 4.5 | 6.9 | |
| SMARCA2 | SWI/SNF related, matrix associated, actin dependent regulator of chromatin, subfamily a | NM_001099115 | -5.1 | 7.8 | 10.2 | |
| ABI3BP | ABI family, member 3 (NESH) binding protein | NM_001080307 /// XM_003581686 /// XM_003585635 | -5.1 | 4.7 | 7.0 | |
| NEDD4L | neural precursor cell expressed, developmentally down-regulated 4-like | XM_002697776 /// XM_587080 | -5.1 | 5.0 | 7.4 | |
| FLT1 | fms-related tyrosine kinase 1 (vascular endothelial growth factor/vascular permeability | NM_001191132 | -5.2 | 4.2 | 6.5 | |
| TPBG | trophoblast glycoprotein | XM_002690047 /// | -5.2 | 7.1 | 9.5 | |

| | | | | | |
|---|---|---|---|---|---|
| | | XM_593502 | | | |
| DUSP1 | dual specificity phosphatase 1 | NM_001046452 | -5.2 | 7.9 | 10.2 |
| CXCL16 | chemokine (C-X-C motif) ligand 16 | NM_001046095 | -5.2 | 5.7 | 8.0 |
| LOC789485 | neurogenic locus notch homolog protein 3-like | XM_003582382 /// XM_003586246 | -5.2 | 6.0 | 8.4 |
| TNXB | tenascin XB | NM_174703 | -5.2 | 4.5 | 6.9 |
| METTL12 | methyltransferase like 12 | NM_001192320 | -5.2 | 6.5 | 8.9 |
| PPFIBP2 | PTPRF interacting protein, binding protein 2 (liprin beta 2) | NM_001193044 /// XM_002693138 /// XM_003583112 /// XM_003586954 | -5.2 | 5.1 | 7.5 |
| NAAA | N-acylethanolamine acid amidase | NM_001100369 | -5.2 | 4.3 | 6.7 |
| SULT1A1 | sulfotransferase family, cytosolic, 1A, phenol-preferring, member 1 | NM_177521 | -5.2 | 6.5 | 8.9 |
| SH3KBP1 | SH3-domain kinase binding protein 1 | NM_001128500 | -5.2 | 5.0 | 7.4 |
| COL24A1 | collagen, type XXIV, alpha 1 | XM_002686308 /// XM_609742 | -5.2 | 3.7 | 6.0 |
| MAP1B | microtubule-associated protein 1B | NM_001206119 | -5.2 | 5.7 | 8.1 |
| SFRP4 | secreted frizzled-related protein 4 | NM_001075764 | -5.2 | 3.9 | 6.3 |
| PALLD | palladin, cytoskeletal associated protein | NM_001193170 /// XM_002689443 /// XM_003582498 | -5.2 | 8.1 | 10.5 |
| TSPAN33 | tetraspanin 33 | NM_001034672 | -5.2 | 4.6 | 7.0 |
| TMTC2 | transmembrane and tetratricopeptide repeat containing 2 | XM_002687180 /// XM_003582183 | -5.3 | 6.2 | 8.6 |
| STK10 | serine/threonine kinase 10 | NM_001192627 | -5.3 | 6.0 | 8.4 |
| XAF1 | XIAP associated factor 1 | NM_001035075 | -5.3 | 5.1 | 7.5 |
| TSC22D3 | TSC22 domain family, member 3 | NM_001103342 | -5.3 | 7.5 | 9.9 |
| PER2 | period homolog 2 (Drosophila) | NM_001192317 /// XM_002686586 | -5.3 | 4.3 | 6.7 |
| LOC100336728 | paladin-like | XM_002698907 | -5.3 | 4.5 | 6.9 |
| TAGLN2 | transgelin 2 | NM_001013599 | -5.3 | 7.1 | 9.5 |

| | | | | | |
|---|---|---|---|---|---|
| IRX3 | iroquois homeobox 3 | NM_001104996 | -5.3 | 4.4 | 6.8 |
| ANGPT2 | angiopoietin 2 | NM_001098855 | -5.3 | 5.8 | 8.2 |
| APOLD1 | apolipoprotein L domain containing 1 | NM_001101180 | -5.3 | 5.0 | 7.4 |
| PLCL2 | phospholipase C-like 2 | NM_001192255 | -5.3 | 5.9 | 8.3 |
| OSTF1 | osteoclast stimulating factor 1 | NM_174409 | -5.3 | 7.9 | 10.3 |
| CD36 | CD36 molecule (thrombospondin receptor) | NM_174010 | -5.3 | 6.3 | 8.7 |
| CD48 | CD48 molecule | NM_001046002 | -5.3 | 4.0 | 6.4 |
| ENDOD1 | endonuclease domain containing 1 | NM_001102519 | -5.3 | 5.2 | 7.6 |
| TCN2 | transcobalamin II | NM_174195 | -5.3 | 6.3 | 8.7 |
| MSR1 | macrophage scavenger receptor 1 | NM_001113240 /// NM_174113 | -5.3 | 4.2 | 6.6 |
| RHOBTB3 | Rho-related BTB domain containing 3 | XM_002689396 /// XM_609411 | -5.3 | 6.1 | 8.5 |
| IQCA1 | IQ motif containing with AAA domain 1 | NM_001206547 | -5.4 | 4.1 | 6.5 |
| DUSP10 | dual specificity phosphatase 10 | NM_001034725 | -5.4 | 4.8 | 7.2 |
| LYZ | lysozyme | NM_001078159 | -5.4 | 4.5 | 6.9 |
| CDH3 | cadherin 3, type 1, P-cadherin (placental) | NM_001244605 | -5.4 | 5.6 | 8.0 |
| CP | ceruloplasmin (ferroxidase) | NM_001256556 /// XM_002685026 /// XM_592003 | -5.4 | 3.2 | 5.6 |
| LYN | v-yes-1 Yamaguchi sarcoma viral related oncogene homolog | NM_001177740 | -5.4 | 4.0 | 6.4 |
| SOCS2 | suppressor of cytokine signaling 2 | NM_177523 | -5.4 | 5.0 | 7.4 |
| C4H7orf41 | chromosome 4 open reading frame, human C7orf41 | NM_001101246 | -5.5 | 5.4 | 7.8 |
| TNC | tenascin C | NM_001078026 | -5.5 | 4.4 | 6.9 |
| SLIT2 | slit homolog 2 (Drosophila) | NM_001191516 | -5.5 | 4.2 | 6.6 |
| TGFB1I1 | transforming growth factor beta 1 induced transcript 1 | NM_001035313 | -5.5 | 5.6 | 8.1 |
| PDGFRL | platelet-derived growth factor receptor-like | NM_001035301 | -5.5 | 5.2 | 7.7 |
| CCDC85B | coiled-coil domain containing 85B | NM_001144087 | -5.5 | 4.5 | 7.0 |
| CYB5R3 | cytochrome b5 reductase 3 | NM_001103250 | -5.5 | 8.9 | 11.3 |
| MGC139164 | uncharacterized LOC509649 | NM_001101916 | -5.5 | 3.4 | 5.9 |

| | | | | | |
|---|---|---|---|---|---|
| CTSF | cathepsin F | NM_001075416 | -5.5 | 5.7 | 8.2 |
| BAMBI | BMP and activin membrane-bound inhibitor homolog (Xenopus laevis) | NM_001046309 | -5.5 | 4.1 | 6.6 |
| TGFBI | transforming growth factor, beta-induced, 68kDa | NM_001205402 | -5.5 | 5.5 | 8.0 |
| PMEPA1 | prostate transmembrane protein, androgen induced 1 | NM_001078078 | -5.6 | 3.5 | 6.0 |
| MICAL1 | microtubule associated monoxygenase, calponin and LIM domain containing 1 | NM_001081582 | -5.6 | 5.0 | 7.5 |
| IMPA1 | inositol(myo)-1(or 4)-monophosphatase 1 | NM_174361 | -5.6 | 5.9 | 8.4 |
| CTSC | cathepsin C | NM_001033617 | -5.6 | 7.8 | 10.2 |
| ALAD | aminolevulinate dehydratase | NM_001014895 | -5.6 | 7.2 | 9.7 |
| CXCL6 | chemokine (C-X-C motif) ligand 6 (granulocyte chemotactic protein 2) | NM_174300 | -5.6 | 3.4 | 5.9 |
| LMO2 | LIM domain only 2 (rhombotin-like 1) | NM_001076352 | -5.6 | 5.2 | 7.7 |
| PTGDS | prostaglandin D2 synthase 21kDa (brain) | NM_174791 | -5.6 | 6.7 | 9.2 |
| SCLY | selenocysteine lyase | NM_001083804 | -5.7 | 4.6 | 7.1 |
| OCIAD2 | OCIA domain containing 2 | NM_001034258 | -5.7 | 5.5 | 8.0 |
| FXYD1 | FXYD domain containing ion transport regulator 1 | NM_001076410 | -5.7 | 4.7 | 7.2 |
| PPP2R2B | protein phosphatase 2, regulatory subunit B, beta | NM_001014879 /// NM_001272085 /// NR_073586 | -5.7 | 4.1 | 6.6 |
| TMEM51 | transmembrane protein 51 | NM_001098975 | -5.7 | 6.0 | 8.5 |
| BASP1 | brain abundant, membrane attached signal protein 1 | NM_174780 | -5.7 | 6.7 | 9.2 |
| CCL2 | chemokine (C-C motif) ligand 2 | NM_174006 | -5.7 | 4.3 | 6.8 |
| LOC512486 | interferon-induced guanylate-binding protein 1 | NM_001244229 | -5.7 | 5.2 | 7.7 |
| SH3BGRL2 | SH3 domain binding glutamic acid-rich protein like 2 | NM_001083791 | -5.7 | 4.8 | 7.3 |
| EHD3 | EH-domain containing 3 | NM_001098004 | -5.7 | 5.1 | 7.6 |
| THBS1 | thrombospondin 1 | NM_174196 | -5.7 | 8.4 | 10.9 |
| TGFBR3 | transforming growth factor, beta receptor III | XM_001253071 /// XM_002686233 | -5.7 | 8.6 | 11.1 |
| PKIB | protein kinase (cAMP-dependent, catalytic) inhibitor beta | NM_001114518 | -5.7 | 3.9 | 6.4 |
| VAMP5 | vesicle-associated membrane protein 5 (myobrevin) | NM_001046476 | -5.7 | 6.8 | 9.3 |

| | | | | | |
|---|---|---|---|---|---|
| TRPM4 | transient receptor potential cation channel, subfamily M, member 4 | XM_002695205 /// XM_002701936 | -5.8 | 5.0 | 7.5 |
| GPRC5C | G protein-coupled receptor, family C, group 5, member C | NM_001272010 /// XM_002696193 /// XM_873143 | -5.8 | 4.9 | 7.4 |
| ANG | angiogenin, ribonuclease, RNase A family, 5 | NM_001078144 | -5.8 | 4.8 | 7.4 |
| TMEM140 | transmembrane protein 140 | NM_001105373 | -5.8 | 4.5 | 7.1 |
| C5H12orf57 | chromosome 5 open reading frame, human C12orf57 | NM_001075525 | -5.8 | 7.3 | 9.8 |
| EPHX1 | epoxide hydrolase 1, microsomal (xenobiotic) | NM_001034629 | -5.8 | 5.4 | 7.9 |
| AKAP1 | A kinase (PRKA) anchor protein 1 | XM_002695587 /// XM_610582 | -5.8 | 6.2 | 8.8 |
| C29H11orf75 /// SMCO4 | chromosome 29 open reading frame, human C11orf75 /// single-pass membrane protein with | XM_002698992 /// XM_002703411 /// XM_002706160 | -5.8 | 5.4 | 8.0 |
| MRC1 | mannose receptor, C type 1 | XM_003582920 /// XM_003582925 /// XM_003586772 | -5.8 | 4.2 | 6.7 |
| PTGER4 | prostaglandin E receptor 4 (subtype EP4) | NM_174589 | -5.8 | 3.8 | 6.4 |
| LOC782642 /// MAL | mal, T-cell differentiation protein-like /// mal, T-cell differentiation protein | NM_001075428 /// XM_001251278 | -5.8 | 4.3 | 6.9 |
| PAK1 | p21 protein (Cdc42/Rac)-activated kinase 1 | NM_001076898 | -5.9 | 4.2 | 6.8 |
| MEIS1 | Meis homeobox 1 | NM_001083507 | -5.9 | 3.9 | 6.5 |
| OIT3 | oncoprotein induced transcript 3 | NM_001046064 | -5.9 | 4.3 | 6.8 |
| BOLA-N /// JSP.1 | MHC class I antigen /// MHC Class I JSP.1 | NM_001040498 /// NM_001105651 | -5.9 | 6.4 | 8.9 |
| SNHG12 | small nucleolar RNA host gene 12 (non-protein coding) | NR_038147 /// NR_038148 | -5.9 | 7.3 | 9.8 |
| CASP4 | caspase 4, apoptosis-related cysteine peptidase | NM_176638 | -5.9 | 4.9 | 7.5 |
| LOC100137759 | N-acetyl-beta-glucosaminyl-glycoprotein 4-beta-N-acetylgalactosaminyltransferase 1-like | XM_003584770 /// XM_003588069 | -5.9 | 3.6 | 6.2 |
| RGS7 | regulator of G-protein signaling 7 | NM_174169 | -6.0 | 4.4 | 7.0 |
| ARHGAP29 | Rho GTPase activating protein 29 | NM_001102485 | -6.0 | 5.2 | 7.7 |
| HHEX | hematopoietically expressed homeobox | NM_001105424 | -6.0 | 3.1 | 5.7 |

| | | | | | |
|---|---|---|---|---|---|
| LOC100849927 /// TINAGL1 | tubulointerstitial nephritis antigen-like /// tubulointerstitial nephritis antigen-like | XM_002685619 /// XM_003585097 /// XM_882308 | -6.0 | 3.9 | 6.5 |
| C28H10orf54 | chromosome 28 open reading frame, human C10orf54 | NM_001080347 | -6.0 | 5.1 | 7.7 |
| PQLC3 | PQ loop repeat containing 3 | NM_001101878 | -6.0 | 5.2 | 7.7 |
| FAM171A1 | family with sequence similarity 171, member A1 | NM_001102180 | -6.0 | 6.2 | 8.8 |
| RGS1 | regulator of G-protein signaling 1 | NM_001199063 | -6.0 | 3.5 | 6.1 |
| SPRY1 | sprouty homolog 1, antagonist of FGF signaling (Drosophila) | NM_001099366 | -6.1 | 5.8 | 8.4 |
| MAOB | monoamine oxidase B | NM_177944 | -6.1 | 5.0 | 7.6 |
| PLEKHO1 | pleckstrin homology domain containing, family O member 1 | NM_001083797 | -6.1 | 5.8 | 8.4 |
| S100A4 | S100 calcium binding protein A4 | NM_174595 | -6.1 | 5.4 | 8.0 |
| AOC3 | amine oxidase, copper containing 3 (vascular adhesion protein 1) | NM_181002 | -6.1 | 4.8 | 7.4 |
| MEST | mesoderm specific transcript homolog (mouse) | NM_001083368 | -6.1 | 6.4 | 9.0 |
| CNN1 | calponin 1, basic, smooth muscle | NM_001046379 | -6.1 | 5.3 | 8.0 |
| BGN | biglycan | NM_178318 | -6.1 | 6.2 | 8.8 |
| MEOX2 | mesenchyme homeobox 2 | NM_001098045 | -6.1 | 3.2 | 5.9 |
| CAST | calpastatin | NM_001030318 /// NM_001030319 /// NM_001030320 /// NM_174003 | -6.2 | 7.5 | 10.1 |
| PDGFC | platelet derived growth factor C | XM_002694441 /// XM_864899 | -6.2 | 7.8 | 10.4 |
| IQGAP2 | IQ motif containing GTPase activating protein 2 | XM_002690436 /// XM_003582648 | -6.2 | 6.0 | 8.6 |
| PSMB10 | proteasome (prosome, macropain) subunit, beta type, 10 | NM_001034040 | -6.2 | 6.3 | 8.9 |
| LRCH1 | leucine-rich repeats and calponin homology (CH) domain containing 1 | XM_002691830 /// XM_581598 | -6.2 | 4.1 | 6.7 |
| KCNJ8 | potassium inwardly-rectifying channel, subfamily J, member 8 | NM_001040482 | -6.2 | 4.5 | 7.1 |
| SLCO2A1 | solute carrier organic anion transporter family, member 2A1 | NM_174829 | -6.2 | 4.1 | 6.7 |

| | | | | | |
|---|---|---|---|---|---|
| APP | amyloid beta (A4) precursor protein | NM_001076796 | -6.2 | 7.3 | 10.0 |
| CYR61 | cysteine-rich, angiogenic inducer, 61 | NM_001034340 | -6.2 | 8.2 | 10.8 |
| TESC | tescalcin | XM_002694586 /// XM_002701712 | -6.3 | 4.7 | 7.3 |
| GJB5 | gap junction protein, beta 5, 31.1kDa | NM_001205907 | -6.3 | 6.2 | 8.8 |
| IMPA2 | inositol(myo)-1(or 4)-monophosphatase 2 | NM_001192282 | -6.3 | 7.2 | 9.9 |
| MEIS2 | Meis homeobox 2 | NM_001076175 /// NM_001191269 | -6.3 | 5.9 | 8.5 |
| LOC100298641 /// LOC614050 | olfactory receptor, family 2, subfamily D, member 2-like /// olfactory receptor, family | XM_002686444 /// XM_002686445 /// XM_865375 | -6.3 | 4.4 | 7.1 |
| TRPC4 | transient receptor potential cation channel, subfamily C, member 4 | NM_174478 | -6.3 | 4.2 | 6.8 |
| CLCA2 | chloride channel accessory 2 | NM_001191300 /// XM_002686307 /// XM_003581991 | -6.3 | 3.4 | 6.1 |
| SEMA5B | sema domain, seven thrombospondin repeats (type 1 and type 1-like), transmembrane domain | XM_001788280 /// XM_002684809 | -6.3 | 5.2 | 7.8 |
| TGFB3 | transforming growth factor, beta 3 | NM_001101183 | -6.3 | 3.9 | 6.5 |
| CD302 | CD302 molecule | NM_001110191 | -6.4 | 5.4 | 8.0 |
| VAV3 | vav 3 guanine nucleotide exchange factor | XM_002686162 /// XM_615898 | -6.4 | 4.0 | 6.7 |
| EPS8 | epidermal growth factor receptor pathway substrate 8 | NM_001076102 /// XM_003582254 /// XM_003586110 | -6.4 | 4.0 | 6.7 |
| C11H2orf40 | chromosome 11 open reading frame, human C2orf40 | NM_001038113 | -6.4 | 5.9 | 8.6 |
| PDLIM2 | PDZ and LIM domain 2 (mystique) | NM_001034430 | -6.4 | 5.2 | 7.9 |
| SLC1A3 | solute carrier family 1 (glial high affinity glutamate transporter), member 3 | NM_174600 | -6.4 | 3.9 | 6.6 |
| EFEMP2 | EGF containing fibulin-like extracellular matrix protein 2 | NM_001076049 | -6.4 | 4.9 | 7.6 |
| LOC100851475 /// LOC100852174 | sushi repeat-containing protein SRPX2-like /// sushi repeat-containing protein SRPX2-li | NM_001014926 /// XM_003584298 /// XM_003585524 | -6.5 | 4.1 | 6.8 |

| | | | | | |
|---|---|---|---|---|---|
| /// SRPX2 | | | | | |
| CFD | complement factor D (adipsin) | NM_001034255 | -6.5 | 5.0 | 7.6 |
| MBP | myelin basic protein | NM_001206674 | -6.5 | 3.9 | 6.6 |
| C1QTNF5 | C1q and tumor necrosis factor related protein 5 | NM_001099138 | -6.5 | 4.9 | 7.6 |
| ANKRD29 | ankyrin repeat domain 29 | NM_001102104 | -6.5 | 4.8 | 7.5 |
| BOLA-DMB | major histocompatibility complex, class II, DM beta | NM_001040481 | -6.5 | 5.4 | 8.1 |
| IGJ | immunoglobulin J polypeptide, linker protein for immunoglobulin alpha and mu polypeptid | NM_175773 | -6.6 | 5.6 | 8.3 |
| HDAC7 | histone deacetylase 7 | NM_001193141 | -6.6 | 5.9 | 8.6 |
| LOC100851529 /// RTP4 | receptor-transporting protein 4-like /// receptor (chemosensory) transporter protein 4 | NM_001075961 /// XM_003585525 | -6.6 | 4.0 | 6.7 |
| CH25H | cholesterol 25-hydroxylase | NM_001075243 | -6.6 | 4.2 | 6.9 |
| RCSD1 | RCSD domain containing 1 | NM_001034418 | -6.7 | 3.8 | 6.5 |
| PEG3 | paternally expressed 3 | NM_001002887 /// XM_003583503 /// XM_003587348 | -6.7 | 8.1 | 10.9 |
| NPVF | neuropeptide VF precursor | NM_174168 | -6.7 | 4.0 | 6.7 |
| OCLN | occludin | NM_001082433 | -6.7 | 4.5 | 7.3 |
| CAMK2N1 | calcium/calmodulin-dependent protein kinase II inhibitor 1 | NM_001114520 | -6.7 | 4.4 | 7.1 |
| ODZ2 | odz, odd Oz/ten-m homolog 2 (Drosophila) | XM_001788056 /// XM_002689363 /// XM_003582481 /// XM_003582482 /// XM_003582483 /// XM | -6.7 | 4.5 | 7.3 |
| MFNG | MFNG O-fucosylpeptide 3-beta-N-acetylglucosaminyltransferase | NM_001101051 | -6.7 | 4.9 | 7.6 |
| C1QB | complement component 1, q subcomponent, B chain | NM_001046599 | -6.7 | 5.2 | 7.9 |
| GATA2 | GATA binding protein 2 | NM_001192114 | -6.7 | 4.0 | 6.7 |
| FXYD5 | FXYD domain containing ion transport regulator 5 | NM_001192063 | -6.8 | 4.6 | 7.3 |
| PRKCH | protein kinase C, eta | NM_001076863 | -6.8 | 4.5 | 7.3 |
| SLC35F2 | solute carrier family 35, member F2 | XM_002692963 /// XM_612258 | -6.9 | 3.9 | 6.7 |

| | | | | | |
|---|---|---|---|---|---|
| SULT1B1 | sulfotransferase family, cytosolic, 1B, member 1 | NM_001075823 | -6.9 | 4.0 | 6.8 |
| SPATS2L | spermatogenesis associated, serine-rich 2-like | NM_001192987 | -6.9 | 7.1 | 9.9 |
| BACE2 | beta-site APP-cleaving enzyme 2 | NM_001206062 | -6.9 | 5.4 | 8.2 |
| MATN2 | matrilin 2 | NM_001102528 | -6.9 | 8.4 | 11.1 |
| TGM2 | transglutaminase 2 (C polypeptide, protein-glutamine-gamma-glutamyltransferase) | NM_177507 | -7.0 | 8.1 | 10.8 |
| RIMS1 | regulating synaptic membrane exocytosis 1 | XM_002690015 /// XM_003582588 /// XM_003582589 /// XM_003586450 /// XM_003586451 /// XM | -7.0 | 3.7 | 6.5 |
| FGL1 | fibrinogen-like 1 | NM_001034313 | -7.0 | 4.5 | 7.3 |
| LARP6 | La ribonucleoprotein domain family, member 6 | NM_001099205 | -7.0 | 4.5 | 7.3 |
| FHL3 | four and a half LIM domains 3 | NM_001034223 | -7.0 | 5.0 | 7.8 |
| LIMS2 | LIM and senescent cell antigen-like domains 2 | NM_001130751 | -7.0 | 5.8 | 8.6 |
| TENC1 | tensin like C1 domain containing phosphatase (tensin 2) | XM_001790201 /// XM_002687243 | -7.1 | 4.7 | 7.5 |
| ASS1 | argininosuccinate synthase 1 | NM_173892 | -7.1 | 5.1 | 7.9 |
| PYROXD2 | pyridine nucleotide-disulphide oxidoreductase domain 2 | NM_001034532 | -7.2 | 4.7 | 7.6 |
| NPDC1 | neural proliferation, differentiation and control, 1 | NM_001105489 | -7.2 | 5.3 | 8.1 |
| GLIPR2 | GLI pathogenesis-related 2 | NM_001076112 | -7.2 | 6.4 | 9.3 |
| PPP1R16B | protein phosphatase 1, regulatory subunit 16B | NM_174824 | -7.2 | 4.4 | 7.2 |
| GJA5 | gap junction protein, alpha 5, 40kDa | NM_001078022 | -7.2 | 4.5 | 7.3 |
| OLFML1 | olfactomedin-like 1 | NM_001035385 | -7.3 | 5.4 | 8.3 |
| ZNF521 | zinc finger protein 521 | NM_001105419 | -7.3 | 4.7 | 7.6 |
| MFAP2 | microfibrillar-associated protein 2 | NM_174388 | -7.3 | 6.2 | 9.1 |
| COTL1 | coactosin-like 1 (Dictyostelium) | NM_001046593 | -7.4 | 4.9 | 7.8 |
| NR2F2 | nuclear receptor subfamily 2, group F, member 2 | NM_174402 | -7.4 | 7.8 | 10.7 |
| LOC504548 /// UBD | ubiquitin D-like /// ubiquitin D | NM_001206473 /// XM_002697377 /// XM_580689 | -7.4 | 4.8 | 7.7 |

| | | | | | |
|---|---|---|---|---|---|
| LOC100849579 /// PDE4B | cAMP-specific 3',5'-cyclic phosphodiesterase 4A-like /// phosphodiesterase 4B, cAMP-spe | NM_001102546 /// XM_003585529 | -7.4 | 4.0 | 6.9 |
| AKAP12 | A kinase (PRKA) anchor protein 12 | XM_002690338 /// XM_591518 | -7.4 | 4.8 | 7.7 |
| FGL2 | fibrinogen-like 2 | NM_001046097 | -7.4 | 4.8 | 7.7 |
| ATP1A2 | ATPase, Na+/K+ transporting, alpha 2 polypeptide | NM_001081524 | -7.5 | 5.3 | 8.2 |
| ISLR | immunoglobulin superfamily containing leucine-rich repeat | NM_001080729 | -7.5 | 5.0 | 7.9 |
| CD200 | CD200 molecule | NM_001034620 | -7.5 | 4.6 | 7.5 |
| CXHXorf57 | chromosome X open reading frame, human CXorf57 | XM_001789923 /// XM_002699835 /// XM_003584302 /// XM_003588125 | -7.6 | 4.6 | 7.5 |
| DHRS7 | dehydrogenase/reductase (SDR family) member 7 | NM_001046162 | -7.6 | 3.9 | 6.8 |
| C4A /// LOC100851057 /// LOC100852118 /// LOC617696 | complement component 4A /// complement C4-A-like /// complement C4-A-like /// complemen | NM_001166485 /// XM_003584610 /// XM_003584815 /// XM_870004 | -7.6 | 7.7 | 10.7 |
| ADA | adenosine deaminase | NM_173887 | -7.6 | 5.1 | 8.0 |
| FREM1 | FRAS1 related extracellular matrix 1 | NM_001192995 | -7.6 | 3.8 | 6.7 |
| SRPX | sushi-repeat containing protein, X-linked | NM_001040489 | -7.6 | 5.7 | 8.6 |
| MAGI1 | membrane associated guanylate kinase, WW and PDZ domain containing 1 | XM_001789358 /// XM_001789365 /// XM_002696963 /// XM_003583802 | -7.7 | 6.6 | 9.6 |
| ACSL5 /// LOC100851804 | acyl-CoA synthetase long-chain family member 5 /// long-chain-fatty-acid--CoA ligase 5- | NM_001075650 /// XM_003585076 | -7.7 | 5.2 | 8.1 |
| IFITM1 | interferon induced transmembrane protein 1 (9-27) | NM_001078142 | -7.7 | 8.7 | 11.7 |
| CXCL3 | chemokine (C-X-C motif) ligand 3 | NM_001046513 | -7.7 | 4.2 | 7.2 |
| TMEM100 | transmembrane protein 100 | NM_001046522 | -7.7 | 5.1 | 8.1 |
| KCTD15 | potassium channel tetramerisation domain containing 15 | NM_001075568 | -7.7 | 4.7 | 7.7 |
| SWAP70 | SWAP switching B-cell complex 70kDa subunit | NM_001080297 | -7.7 | 4.8 | 7.7 |

| | | | | | |
|---|---|---|---|---|---|
| CORO1A | coronin, actin binding protein, 1A | NM_174521 | -7.8 | 4.1 | 7.1 |
| ANKH | ankylosis, progressive homolog (mouse) | NM_001109793 | -7.8 | 5.0 | 8.0 |
| CALML4 | calmodulin-like 4 | NM_001034671 | -7.8 | 4.4 | 7.4 |
| HFM1 | HFM1, ATP-dependent DNA helicase homolog (S. cerevisiae) | NM_001205576 | -7.8 | 3.0 | 5.9 |
| FRMD6 /// LOC100335364 | FERM domain containing 6 /// FERM domain-containing protein 6-like | NM_001102133 /// XM_002700610 | -7.8 | 4.8 | 7.8 |
| TACC2 | transforming, acidic coiled-coil containing protein 2 | NM_001102159 /// XM_003584113 /// XM_003584114 /// XM_003587903 | -7.8 | 5.6 | 8.6 |
| ICAM3 | intercellular adhesion molecule 3 | NM_174349 | -7.8 | 4.6 | 7.5 |
| PLIN5 | perilipin 5 | NM_001101136 | -7.8 | 3.7 | 6.7 |
| DBNDD2 | dysbindin (dystrobrevin binding protein 1) domain containing 2 | NM_001130748 | -7.8 | 4.0 | 6.9 |
| C1QC | complement component 1, q subcomponent, C chain | NM_001206396 | -7.8 | 4.6 | 7.6 |
| LOC100849300 /// MAP2K6 | dual specificity mitogen-activated protein kinase kinase 6-like /// mitogen-activated p | NM_001034045 /// XM_003585085 | -7.9 | 4.4 | 7.4 |
| ARAP3 | ArfGAP with RhoGAP domain, ankyrin repeat and PH domain 3 | XM_003582447 /// XM_003586307 | -8.0 | 4.3 | 7.3 |
| CD300LG | CD300 molecule-like family member g | XM_002696037 /// XM_867184 | -8.0 | 4.5 | 7.5 |
| PEAR1 | platelet endothelial aggregation receptor 1 | NM_001101300 | -8.0 | 5.2 | 8.2 |
| PRKCDBP | protein kinase C, delta binding protein | NM_001083401 | -8.1 | 5.9 | 8.9 |
| NRP2 | neuropilin 2 | NM_001193237 | -8.1 | 4.5 | 7.5 |
| MYH11 | myosin, heavy chain 11, smooth muscle | NM_001102127 | -8.1 | 7.5 | 10.5 |
| RGS16 | regulator of G-protein signaling 16 | NM_174450 | -8.1 | 4.3 | 7.3 |
| S100A10 | S100 calcium binding protein A10 | NM_174650 | -8.1 | 8.0 | 11.0 |
| C16H1orf115 | chromosome 16 open reading frame, human C1orf115 | NM_001205383 | -8.2 | 6.5 | 9.5 |
| MYO10 | myosin X | NM_174394 | -8.2 | 5.8 | 8.8 |
| CLU | clusterin | NM_173902 | -8.3 | 7.2 | 10.3 |
| PLAC8 | placenta-specific 8 | NM_001076987 | -8.3 | 4.8 | 7.9 |

| | | | | | |
|---|---|---|---|---|---|
| MRVI1 | murine retrovirus integration site 1 homolog | NM_174392 /// NM_194465 | -8.3 | 4.8 | 7.8 |
| CD55 | CD55 molecule, decay accelerating factor for complement (Cromer blood group) | NM_001030303 | -8.4 | 4.7 | 7.8 |
| DNER | delta/notch-like EGF repeat containing | XM_003585079 /// XM_003585774 /// XM_594984 | -8.4 | 3.9 | 7.0 |
| EML1 | echinoderm microtubule associated protein like 1 | XM_002696768 /// XM_590509 | -8.4 | 4.5 | 7.5 |
| KLF2 | Kruppel-like factor 2 (lung) | XM_001787366 /// XM_002688582 | -8.5 | 5.1 | 8.2 |
| DKK3 | dickkopf homolog 3 (Xenopus laevis) | NM_001100306 | -8.5 | 6.6 | 9.7 |
| LOC508666 | C-C motif chemokine 23 | NM_001244199 | -8.6 | 6.6 | 9.7 |
| NDRG1 | N-myc downstream regulated 1 | NM_001035009 | -8.6 | 4.7 | 7.8 |
| FHOD1 | formin homology 2 domain containing 1 | NM_001206339 | -8.6 | 4.9 | 8.0 |
| HSPB8 | heat shock 22kDa protein 8 | NM_001014955 | -8.7 | 5.0 | 8.1 |
| MALL | mal, T-cell differentiation protein-like | NM_001046115 | -8.7 | 3.5 | 6.6 |
| FBLIM1 | filamin binding LIM protein 1 | NM_001076955 | -8.7 | 6.2 | 9.3 |
| AS3MT /// AS3MT | arsenic (+3 oxidation state) methyltransferase /// arsenic (+3 oxidation state) methylt | NM_001035023 /// XM_001255467 | -8.7 | 6.1 | 9.2 |
| TIE1 | tyrosine kinase with immunoglobulin-like and EGF-like domains 1 | NM_173965 | -8.7 | 4.0 | 7.1 |
| LOX | lysyl oxidase | NM_173932 | -8.8 | 4.3 | 7.4 |
| ZFP36L1 | zinc finger protein 36, C3H type-like 1 | NM_001101234 | -8.8 | 8.2 | 11.3 |
| ETS2 | v-ets erythroblastosis virus E26 oncogene homolog 2 (avian) | NM_001080214 | -8.8 | 4.0 | 7.2 |
| FOLH1 | folate hydrolase (prostate-specific membrane antigen) 1 | NM_001101858 | -8.9 | 5.9 | 9.1 |
| SH3BP5 | SH3-domain binding protein 5 (BTK-associated) | NM_001206288 | -8.9 | 5.3 | 8.4 |
| PPP1R3C | protein phosphatase 1, regulatory subunit 3C | NM_001076164 | -8.9 | 3.9 | 7.1 |
| SCG2 | secretogranin II | NM_174176 | -8.9 | 5.8 | 9.0 |
| CPE | carboxypeptidase E | NM_173903 | -8.9 | 4.5 | 7.7 |
| IQSEC1 | IQ motif and Sec7 domain 1 | NM_001206814 | -8.9 | 4.6 | 7.8 |

| | | | | | |
|---|---|---|---|---|---|
| SCHIP1 | schwannomin interacting protein 1 | NM_001046412 | -9.0 | 5.7 | 8.8 |
| NXN | nucleoredoxin | NM_001102136 | -9.0 | 5.2 | 8.4 |
| C1S | complement component 1, s subcomponent | NM_001076550 | -9.0 | 5.3 | 8.5 |
| GNG2 | guanine nucleotide binding protein (G protein), gamma 2 | NM_174072 | -9.0 | 5.6 | 8.8 |
| ADAMTS1 | ADAM metallopeptidase with thrombospondin type 1 motif, 1 | NM_001101080 | -9.0 | 6.9 | 10.0 |
| MAN1C1 | mannosidase, alpha, class 1C, member 1 | XM_002685706 /// XM_866713 | -9.0 | 6.8 | 10.0 |
| GLUL | glutamate-ammonia ligase | NM_001040474 | -9.1 | 8.4 | 11.6 |
| FRMD4A | FERM domain containing 4A | NM_001192267 | -9.1 | 4.2 | 7.4 |
| THBS2 | thrombospondin 2 | NM_176872 | -9.2 | 6.1 | 9.2 |
| TMEM88 | transmembrane protein 88 | NM_001098378 | -9.2 | 4.8 | 8.0 |
| KLHL23 | kelch-like 23 (Drosophila) | NM_001205507 | -9.2 | 4.0 | 7.2 |
| CAPG | capping protein (actin filament), gelsolin-like | NM_178574 | -9.2 | 5.3 | 8.5 |
| LOC509972 | C-type lectin domain family 2 member D11-like | XM_002687834 /// XM_003582258 /// XM_003586126 /// XM_587046 | -9.2 | 3.4 | 6.6 |
| LOC504773 | regakine 1 | NM_001034220 | -9.2 | 5.6 | 8.9 |
| SLC9A3R2 | solute carrier family 9 (sodium/hydrogen exchanger), member 3 regulator 2 | NM_001077065 | -9.3 | 4.9 | 8.1 |
| EPB41L4A | erythrocyte membrane protein band 4.1 like 4A | NM_001105384 | -9.3 | 4.9 | 8.1 |
| TCF4 | transcription factor 4 | NM_001034621 | -9.3 | 5.0 | 8.2 |
| BLA-DQB /// LA-DQB | MHC class II antigen /// MHC cell surface glycoprotein | NM_001034668 /// NM_001080923 | -9.3 | 6.9 | 10.2 |
| PALMD | palmdelphin | NM_001035079 | -9.4 | 4.3 | 7.5 |
| OSR2 | odd-skipped related 2 (Drosophila) | NM_001034328 | -9.4 | 3.7 | 6.9 |
| PGM5 | phosphoglucomutase 5 | NM_001102335 | -9.5 | 4.8 | 8.0 |
| COL4A6 | collagen, type IV, alpha 6 | XM_002699886 /// XM_601826 | -9.5 | 4.8 | 8.0 |
| BICC1 | bicaudal C homolog 1 (Drosophila) | XM_002698825 /// XM_617983 | -9.5 | 3.9 | 7.1 |

| | | | | | | |
|---|---|---|---|---|---|---|
| SEMA5A | sema domain, seven thrombospondin repeats (type 1 and type 1-like), transmembrane domai | XM_002696441 /// XM_583112 | -9.5 | 4.8 | 8.0 | |
| RFTN2 | raftlin family member 2 | NM_001097986 | -9.6 | 3.6 | 6.9 | |
| P4HA3 | prolyl 4-hydroxylase, alpha polypeptide III | NM_001001598 | -9.7 | 4.7 | 8.0 | |
| SELENBP1 | selenium binding protein 1 | NM_001046048 | -9.8 | 4.8 | 8.1 | |
| CD44 | CD44 molecule (Indian blood group) | NM_174013 | -9.8 | 3.8 | 7.1 | |
| LPL | lipoprotein lipase | NM_001075120 | -9.8 | 6.4 | 9.7 | |
| TGFB2 | transforming growth factor, beta 2 | NM_001113252 | -9.8 | 3.5 | 6.8 | |
| NEDD9 | neural precursor cell expressed, developmentally down-regulated 9 | NM_001101847 | -9.9 | 6.1 | 9.4 | |
| DSC2 | desmocollin 2 | NM_001166526 | -9.9 | 4.1 | 7.4 | |
| TIMP3 | TIMP metallopeptidase inhibitor 3 | NM_174473 | -10.0 | 4.4 | 7.7 | |
| CCL14 | chemokine (C-C motif) ligand 14 | NM_001046585 | -10.0 | 4.4 | 7.7 | |
| MFAP5 | microfibrillar associated protein 5 | NM_174386 | -10.0 | 4.9 | 8.2 | |
| NUAK1 | NUAK family, SNF1-like kinase, 1 | NM_001205496 | -10.0 | 5.6 | 8.9 | |
| FKBP11 | FK506 binding protein 11, 19 kDa | NM_001045932 | -10.1 | 6.4 | 9.8 | |
| PLAGL1 | pleiomorphic adenoma gene-like 1 | NM_001103289 | -10.1 | 4.6 | 8.0 | |
| KRT10 /// LOC100138974 | keratin 10 /// keratin 13-like | NM_174377 /// XM_001788728 /// XM_003585337 | -10.1 | 4.3 | 7.7 | |
| TCF21 | transcription factor 21 | NM_001014899 | -10.2 | 7.4 | 10.7 | |
| RBPMS | RNA binding protein with multiple splicing | NM_001046535 | -10.2 | 4.2 | 7.5 | |
| HES1 | hairy and enhancer of split 1, (Drosophila) | NM_001034678 | -10.2 | 4.1 | 7.5 | |
| EHD4 | EH-domain containing 4 | NM_001192053 | -10.2 | 4.9 | 8.2 | |
| AHNAK | --- | --- | | 7.4 | 10.7 | |
| CDH5 | cadherin 5, type 2 (vascular endothelium) | NM_001001601 | -10.2 | 5.3 | 8.7 | - 10.2 |
| TFPI2 | tissue factor pathway inhibitor 2 | NM_182788 | -10.3 | 4.9 | 8.3 | |
| MYOC | myocilin, trabecular meshwork inducible glucocorticoid response | NM_174118 | -10.4 | 4.6 | 8.0 | |
| ROBO4 | roundabout homolog 4, magic roundabout (Drosophila) | NM_001099191 | -10.4 | 5.2 | 8.6 | |

| | | | | | |
|---|---|---|---|---|---|
| PLN | phospholamban | NM_001103319 | -10.4 | 4.2 | 7.5 |
| SASH1 | SAM and SH3 domain containing 1 | XM_002690315 /// XM_588302 | -10.5 | 5.0 | 8.4 |
| LAPTM5 | lysosomal protein transmembrane 5 | NM_001046118 | -10.5 | 4.9 | 8.3 |
| ICAM1 | intercellular adhesion molecule 1 | NM_174348 | -10.5 | 4.9 | 8.3 |
| HLF | hepatic leukemia factor | NM_001192437 | -10.6 | 4.1 | 7.5 |
| EVA1B | eva-1 homolog B (C. elegans) | NM_001101271 | -10.7 | 5.9 | 9.3 |
| CXCL10 | chemokine (C-X-C motif) ligand 10 | NM_001046551 | -10.8 | 4.1 | 7.5 |
| LRRC34 | leucine rich repeat containing 34 | XM_002684932 /// XM_590280 | -10.8 | 3.2 | 6.6 |
| TPM2 | tropomyosin 2 (beta) | NM_001010995 | -10.8 | 7.2 | 10.6 |
| KLF4 | Kruppel-like factor 4 (gut) | NM_001105385 | -10.9 | 4.2 | 7.7 |
| MMRN1 | multimerin 1 | NM_001102500 | -10.9 | 3.8 | 7.2 |
| ARHGEF25 | Rho guanine nucleotide exchange factor (GEF) 25 | NM_001024481 | -11.0 | 4.4 | 7.9 |
| C1QA | complement component 1, q subcomponent, A chain | NM_001014945 | -11.0 | 5.8 | 9.2 |
| NR2F1 | nuclear receptor subfamily 2, group F, member 1 | NM_175804 | -11.0 | 5.9 | 9.3 |
| BOLA-DRB3 /// LOC100851018 | major histocompatibility complex, class II, DRB3 /// DLA class II histocompatibility an | NM_001012680 /// XM_003585221 | -11.0 | 4.5 | 8.0 |
| RFTN1 | raftlin, lipid raft linker 1 | XM_002685189 /// XM_583278 | -11.0 | 5.4 | 8.8 |
| TMEM150C | transmembrane protein 150C | NM_001078001 | -11.0 | 3.9 | 7.4 |
| NCAM1 | neural cell adhesion molecule 1 | NM_174399 | -11.0 | 4.2 | 7.7 |
| ADCY2 | adenylate cyclase 2 (brain) | XM_002696432 /// XM_587884 | -11.1 | 3.7 | 7.2 |
| LRRC17 | leucine rich repeat containing 17 | NM_001078150 | -11.1 | 5.5 | 9.0 |
| EFNA1 | ephrin-A1 | NM_001034292 | -11.2 | 5.0 | 8.5 |
| RHOJ | ras homolog gene family, member J | NM_001083498 | -11.2 | 3.3 | 6.8 |
| HEPH | hephaestin | XM_002700167 /// XM_003584900 /// XM_587920 | -11.2 | 4.4 | 7.9 |

| | | | | | |
|---|---|---|---|---|---|
| RASSF3 | Ras association (RalGDS/AF-6) domain family member 3 | NM_001192886 | -11.2 | 5.2 | 8.7 |
| ADAM19 | ADAM metallopeptidase domain 19 | NM_001075475 | -11.2 | 4.7 | 8.2 |
| IGFBP6 | insulin-like growth factor binding protein 6 | NM_001040495 | -11.3 | 7.7 | 11.2 |
| PTGIS | prostaglandin I2 (prostacyclin) synthase | NM_174444 | -11.4 | 4.3 | 7.8 |
| FAM43A | family with sequence similarity 43, member A | XM_002684829 /// XM_586734 | -11.5 | 4.4 | 7.9 |
| PTPLAD2 | protein tyrosine phosphatase-like A domain containing 2 | NM_001076522 | -11.5 | 4.7 | 8.2 |
| S100B | S100 calcium binding protein B | NM_001034555 | -11.6 | 5.5 | 9.1 |
| CD34 | CD34 molecule | NM_174009 | -11.6 | 4.7 | 8.3 |
| PSAT1 | phosphoserine aminotransferase 1 | NM_001102150 | -11.6 | 6.6 | 10.1 |
| LUM | lumican | NM_173934 | -11.7 | 7.2 | 10.8 |
| ECSCR | endothelial cell-specific chemotaxis regulator | NM_001046099 /// NM_001244445 /// NM_001244446 | -11.8 | 5.2 | 8.7 |
| LOC100297676 | C-type lectin domain family 2 member G-like | XM_002687838 /// XM_002704428 | -11.9 | 4.9 | 8.5 |
| ARHGDIB | Rho GDP dissociation inhibitor (GDI) beta | NM_175797 | -11.9 | 5.2 | 8.8 |
| RSPO3 | R-spondin 3 | NM_001076034 | -11.9 | 4.6 | 8.2 |
| SPARC | secreted protein, acidic, cysteine-rich (osteonectin) | NM_174464 | -12.0 | 8.8 | 12.4 |
| SERTAD4 | SERTA domain containing 4 | NM_001193037 | -12.0 | 3.6 | 7.2 |
| HOXD9 | homeobox D9 | XM_002685288 /// XM_002703599 | -12.1 | 5.0 | 8.6 |
| FOS | FBJ murine osteosarcoma viral oncogene homolog | NM_182786 | -12.1 | 6.1 | 9.7 |
| Sep-04 | septin 4 | NM_001034651 | -12.1 | 4.9 | 8.5 |
| LYVE1 | lymphatic vessel endothelial hyaluronan receptor 1 | NM_205815 | -12.2 | 4.4 | 8.0 |
| AEBP1 | AE binding protein 1 | NM_174839 | -12.2 | 5.0 | 8.6 |
| WIPF1 | WAS/WASL interacting protein family, member 1 | NM_001076923 | -12.3 | 4.8 | 8.4 |
| CLDN5 | claudin 5 | NM_001076460 | -12.3 | 5.1 | 8.7 |
| SVIL | supervillin | NM_174190 | -12.4 | 5.5 | 9.1 |
| RHOU | ras homolog gene family, member U | NM_001098147 | -12.4 | 4.5 | 8.1 |

| | | | | | |
|---|---|---|---|---|---|
| ACTG2 | actin, gamma 2, smooth muscle, enteric | NM_001013592 | -12.5 | 7.2 | 10.8 |
| AGRN | agrin | XM_002694193 /// XM_604151 | -12.5 | 6.1 | 9.7 |
| PCSK6 | proprotein convertase subtilisin/kexin type 6 | XM_002696654 /// XM_603014 | -12.5 | 3.9 | 7.6 |
| SHANK3 | SH3 and multiple ankyrin repeat domains 3 | XM_002687990 /// XM_589942 | -12.6 | 4.6 | 8.3 |
| CXCR4 | chemokine (C-X-C motif) receptor 4 | NM_174301 | -12.7 | 4.1 | 7.7 |
| FABP5 | fatty acid binding protein 5 (psoriasis-associated) | NM_174315 | -12.7 | 4.5 | 8.2 |
| CYP17A1 | cytochrome P450, subfamily XVII | NM_174304 | -12.7 | 5.4 | 9.1 |
| PTPRB | protein tyrosine phosphatase, receptor type, B | NM_001205297 | -12.9 | 4.0 | 7.7 |
| ANXA3 | annexin A3 | NM_001035325 | -12.9 | 5.1 | 8.7 |
| PDK4 | pyruvate dehydrogenase kinase, isozyme 4 | NM_001101883 | -13.0 | 5.1 | 8.8 |
| LRRFIP1 | leucine rich repeat (in FLII) interacting protein 1 | NM_001102308 | -13.1 | 4.6 | 8.3 |
| RYR3 | ryanodine receptor 3 | XM_002690724 /// XM_003584950 /// XM_590220 | -13.1 | 3.4 | 7.1 |
| LDB2 | LIM domain binding 2 | NM_001046611 | -13.2 | 4.7 | 8.5 |
| JAG1 | jagged 1 | NM_001191178 | -13.3 | 5.8 | 9.5 |
| ERG | v-ets erythroblastosis virus E26 oncogene homolog (avian) | NM_001102183 | -13.3 | 4.2 | 8.0 |
| TMEM119 | transmembrane protein 119 | NM_001083664 | -13.3 | 5.0 | 8.8 |
| WFDC1 | WAP four-disulfide core domain 1 | XM_002694744 /// XM_581642 | -13.3 | 5.6 | 9.3 |
| SDC2 | syndecan 2 | NM_001034788 | -13.5 | 6.6 | 10.3 |
| REM1 | RAS (RAD and GEM)-like GTP-binding 1 | NM_001046001 | -13.5 | 3.1 | 6.9 |
| C1QTNF7 | C1q and tumor necrosis factor related protein 7 | NM_001076201 | -13.6 | 3.9 | 7.7 |
| ALDH1A2 | aldehyde dehydrogenase 1 family, member A2 | XM_002690855 /// XM_615062 | -13.7 | 4.6 | 8.3 |
| NTRK2 | neurotrophic tyrosine kinase, receptor, type 2 | NM_001075225 | -13.8 | 4.0 | 7.8 |
| STAR | steroidogenic acute regulatory protein | NM_174189 | -14.2 | 6.2 | 10.1 |
| NOV | nephroblastoma overexpressed gene | NM_001102382 | -14.3 | 5.9 | 9.7 |

| | | | | | |
|---|---|---|---|---|---|
| COL27A1 | collagen, type XXVII, alpha 1 | NM_001206680 | -14.5 | 6.7 | 10.6 |
| RNASE6 | ribonuclease, RNase A family, k6 | NM_174594 | -14.7 | 4.5 | 8.4 |
| LBH | limb bud and heart development homolog (mouse) | NM_001099152 | -14.8 | 4.4 | 8.3 |
| COLEC12 | collectin sub-family member 12 | NM_001101843 | -14.9 | 4.6 | 8.5 |
| LRRC70 | leucine rich repeat containing 70 | XM_002696307 /// XM_002702499 | -14.9 | 4.3 | 8.2 |
| LOC100851458 | CUGBP Elav-like family member 2-like | XM_003582899 | -15.0 | 3.4 | 7.3 |
| GPR116 | G protein-coupled receptor 116 | NM_001193243 | -15.0 | 4.3 | 8.2 |
| GRB10 | growth factor receptor-bound protein 10 | NM_001192586 | -15.0 | 3.9 | 7.8 |
| PTH1R | parathyroid hormone 1 receptor | NM_001075332 | -15.2 | 4.1 | 8.0 |
| MYC | v-myc myelocytomatosis viral oncogene homolog (avian) | NM_001046074 | -15.3 | 6.1 | 10.0 |
| HEYL | hairy/enhancer-of-split related with YRPW motif-like | NM_001024565 | -15.4 | 4.6 | 8.5 |
| ITGB3 | integrin, beta 3 (platelet glycoprotein IIIa, antigen CD61) | NM_001206490 | -15.5 | 4.8 | 8.8 |
| ENPP1 | ectonucleotide pyrophosphatase/phosphodiesterase 1 | NM_001206212 | -15.6 | 5.1 | 9.0 |
| TEK | TEK tyrosine kinase, endothelial | NM_173964 | -15.7 | 4.8 | 8.8 |
| LPAR6 | lysophosphatidic acid receptor 6 | NM_001101284 | -15.7 | 3.3 | 7.3 |
| TM4SF18 | transmembrane 4 L six family member 18 | NM_001034287 /// NM_001184724 | -15.7 | 4.3 | 8.3 |
| LOC783399 | major allergen Equ c 1-like | XM_002689933 /// XM_003582575 | -15.8 | 4.3 | 8.2 |
| GPM6A | glycoprotein M6A | NM_001075309 | -15.9 | 4.2 | 8.2 |
| EMP1 | epithelial membrane protein 1 | XM_001251759 /// XM_002687764 | -15.9 | 4.1 | 8.1 |
| INSL3 | insulin-like 3 (Leydig cell) | NM_174365 | -16.1 | 9.0 | 13.1 |
| EMILIN1 | elastin microfibril interfacer 1 | NM_001192434 | -16.4 | 5.1 | 9.1 |
| DAB2 | disabled homolog 2, mitogen-responsive phosphoprotein (Drosophila) | NM_001193246 | -16.5 | 5.3 | 9.3 |
| ANXA1 | annexin A1 | NM_175784 | -16.5 | 6.6 | 10.7 |
| AGTR1 | angiotensin II receptor, type 1 | NM_174233 | -16.7 | 4.1 | 8.1 |
| PDLIM1 | PDZ and LIM domain 1 | NM_001035455 | -16.8 | 6.1 | 10.2 |
| LAMA4 | laminin, alpha 4 | NM_001205965 | -17.1 | 4.8 | 8.9 |

| | | | | | | |
|---|---|---|---|---|---|---|
| CHST7 | carbohydrate (N-acetylglucosamine 6-O) sulfotransferase 7 | NM_001193203 | -17.2 | 4.6 | 8.7 | |
| CTSS | cathepsin S | NM_001033615 | -17.3 | 5.5 | 9.6 | |
| IGFBP3 | insulin-like growth factor binding protein 3 | NM_174556 /// XM_003582113 | -17.5 | 5.8 | 9.9 | |
| ID1 | inhibitor of DNA binding 1, dominant negative helix-loop-helix protein | NM_001097568 | -17.6 | 5.5 | 9.7 | |
| BOLA-DRB3 | major histocompatibility complex, class II, DRB3 | NM_001012680 | -17.6 | 5.3 | 9.5 | |
| NRK | Nik related kinase | XM_002699807 | -17.7 | 4.0 | 8.2 | |
| NRIP3 | nuclear receptor interacting protein 3 | NM_001102218 | -18.0 | 4.6 | 8.8 | |
| PECAM1 | platelet/endothelial cell adhesion molecule | NM_174571 | -18.0 | 4.8 | 8.9 | |
| LOC100848920 /// RGS2 | regulator of G-protein signaling 2-like /// regulator of G-protein signaling 2, 24kDa | NM_001075596 /// XM_003583217 /// XM_003587052 | -18.1 | 4.4 | 8.5 | |
| FHL2 | four and a half LIM domains 2 | NM_001046046 | -18.1 | 6.5 | 10.6 | |
| AIM2 | absent in melanoma 2 | --- | | 4.0 | 8.2 | |
| COL18A1 | collagen, type XVIII, alpha 1 | NM_001083388 | -18.2 | 6.1 | 10.2 | - 18.2 |
| LOC518495 | apolipoprotein L3-like | XM_003582237 /// XM_003586098 | -18.5 | 4.5 | 8.7 | |
| KDR | kinase insert domain receptor (a type III receptor tyrosine kinase) | NM_001110000 | -18.5 | 4.1 | 8.3 | |
| KCNE4 | potassium voltage-gated channel, Isk-related family, member 4 | NM_001081543 | -18.6 | 5.0 | 9.3 | |
| CXCR7 | chemokine (C-X-C motif) receptor 7 | NM_001098381 | -18.7 | 4.6 | 8.8 | |
| KIT | v-kit Hardy-Zuckerman 4 feline sarcoma viral oncogene homolog | NM_001166484 | -18.8 | 4.2 | 8.4 | |
| GGTA1 | alpha-galactosyltransferase 1 (glycoprotein) | NM_177511 | -18.9 | 4.1 | 8.3 | |
| MMP23B | matrix metallopeptidase 23B | NM_001038556 | -19.2 | 5.2 | 9.5 | |
| EMCN | endomucin | NM_001076420 | -19.3 | 4.8 | 9.1 | |
| SYTL2 | synaptotagmin-like 2 | NM_001102278 | -19.5 | 3.4 | 7.7 | |
| AXL | AXL receptor tyrosine kinase | XM_002695068 /// XM_594754 | -19.6 | 4.8 | 9.1 | |

| | | | | | |
|---|---|---|---|---|---|
| LOC539690 | complement component C1q receptor-like | XM_003582935 /// XM_003586784 | -19.7 | 4.8 | 9.1 |
| STC1 | stanniocalcin 1 | NM_176669 | -20.2 | 4.9 | 9.3 |
| OLFML3 | olfactomedin-like 3 | NM_001075197 | -20.4 | 5.2 | 9.5 |
| LOC100850955 | myosin light chain kinase, smooth muscle-like | XM_003581710 | -20.5 | 7.5 | 11.9 |
| CYBRD1 | cytochrome b reductase 1 | NM_001206049 | -20.5 | 5.1 | 9.4 |
| CTGF | connective tissue growth factor | NM_174030 | -20.5 | 7.2 | 11.6 |
| LOC783195 /// RNASE4 | ribonuclease 4-like /// ribonuclease, RNase A family, 4 | NM_001040590 /// XM_001251830 /// XM_002690653 | -20.6 | 5.1 | 9.4 |
| C27H8orf4 | chromosome 27 open reading frame, human C8orf4 | NM_001035490 | -20.8 | 4.4 | 8.8 |
| CTSH | cathepsin H | NM_001034385 | -20.8 | 4.7 | 9.1 |
| SOX18 | SRY (sex determining region Y)-box 18 | NM_001075789 | -20.9 | 3.9 | 8.3 |
| CALCRL | calcitonin receptor-like | NM_001102107 | -20.9 | 4.6 | 9.0 |
| LOC100337023 | collagen alpha-1(V) chain-like | XR_139433 | -21.0 | 7.0 | 11.4 |
| CFH | complement factor H | NM_001033936 | -21.1 | 4.2 | 8.6 |
| VIPR2 | vasoactive intestinal peptide receptor 2 | NM_001206781 | -21.1 | 6.0 | 10.4 |
| SCUBE2 | signal peptide, CUB domain, EGF-like 2 | XM_002693059 /// XM_003583105 /// XM_003586953 /// XM_608409 | -21.6 | 4.6 | 9.0 |
| LAMB1 | laminin, beta 1 | NM_001206519 | -21.9 | 6.0 | 10.5 |
| CDKN1C | cyclin-dependent kinase inhibitor 1C (p57, Kip2) | NM_001077903 | -21.9 | 5.2 | 9.6 |
| CLEC14A | C-type lectin domain family 14, member A | NM_001077890 | -22.1 | 3.8 | 8.3 |
| TM4SF1 | transmembrane 4 L six family member 1 | NM_001075980 | -22.1 | 4.7 | 9.1 |
| AQP1 | aquaporin 1 (Colton blood group) | NM_174702 | -22.3 | 4.1 | 8.6 |
| GYPC | glycophorin C (Gerbich blood group) | NM_001002886 | -22.7 | 5.5 | 10.0 |
| SULF2 | sulfatase 2 | NM_001192938 | -22.8 | 4.2 | 8.7 |
| MEG3 | maternally expressed 3 (non-protein coding) | NR_037684 | -22.8 | 4.6 | 9.1 |
| PDGFRA | platelet-derived growth factor receptor, alpha polypeptide | NM_001192345 | -23.0 | 4.9 | 9.5 |

| | | | | | |
|---|---|---|---|---|---|
| COL11A1 | collagen, type XI, alpha 1 | NM_001166509 | -23.0 | 4.2 | 8.7 |
| BOLA-DRA | major histocompatibility complex, class II, DR alpha | NM_001012677 | -23.0 | 5.5 | 10.0 |
| VSTM4 | V-set and transmembrane domain containing 4 | XM_002698947 /// XM_003584215 | -23.2 | 3.8 | 8.4 |
| PDLIM3 | PDZ and LIM domain 3 | NM_001034646 | -23.3 | 4.7 | 9.2 |
| DLC1 | deleted in liver cancer 1 | NM_001102493 | -23.5 | 4.7 | 9.2 |
| DCLK1 | doublecortin-like kinase 1 | NM_001109962 | -23.9 | 5.1 | 9.6 |
| PMP22 | peripheral myelin protein 22 | NM_001101156 | -24.0 | 5.6 | 10.2 |
| TMEM204 | transmembrane protein 204 | NM_001076377 | -24.5 | 4.2 | 8.8 |
| H19 | H19, imprinted maternally expressed transcript (non-protein coding) | NR_003958 | -24.6 | 5.4 | 10.0 |
| FMOD | fibromodulin | NM_174058 | -25.0 | 5.6 | 10.2 |
| LMO7 | LIM domain 7 | NM_001109801 /// XM_003582872 /// XM_003582873 /// XM_003586719 /// XM_003586720 | -25.1 | 3.7 | 8.4 |
| TBX3 | T-box 3 | XM_001787821 /// XM_002694588 | -25.9 | 3.8 | 8.5 |
| COL1A1 | collagen, type I, alpha 1 | NM_001034039 | -26.0 | 6.2 | 10.9 |
| C7 | complement component 7 | NM_001045966 | -26.2 | 4.0 | 8.7 |
| BRB | brain ribonuclease | NM_173891 | -26.4 | 3.5 | 8.2 |
| ELTD1 | EGF, latrophilin and seven transmembrane domain containing 1 | NM_001076908 | -27.4 | 4.1 | 8.9 |
| PHLDB2 | pleckstrin homology-like domain, family B, member 2 | NM_001206308 | -27.8 | 3.7 | 8.5 |
| PXDN | peroxidasin homolog (Drosophila) | XM_002683948 /// XM_593953 | -27.9 | 5.0 | 9.8 |
| SH3BGR | SH3 domain binding glutamic acid-rich protein | NM_001243326 | -28.0 | 4.5 | 9.3 |
| FNBP1 | formin binding protein 1 | NM_001206712 | -28.1 | 4.2 | 9.0 |
| CXCL12 | chemokine (C-X-C motif) ligand 12 | NM_001113174 | -28.1 | 4.3 | 9.1 |
| LMCD1 | LIM and cysteine-rich domains 1 | NM_001076222 | -28.2 | 4.5 | 9.3 |

| | | | | | |
|---|---|---|---|---|---|
| DPYSL3 | dihydropyrimidinase-like 3 | NM_001101068 | -28.7 | 5.6 | 10.5 |
| NFIB | nuclear factor I/B | NM_001076104 | -28.7 | 4.3 | 9.2 |
| LOC100336224 /// NEK6 | serine/threonine-protein kinase Nek6-like /// NIMA (never in mitosis gene a)-related ki | NM_001098988 /// XM_002706994 | -29.0 | 3.7 | 8.6 |
| DPT | dermatopontin | NM_001045903 | -29.9 | 3.8 | 8.7 |
| NID2 | nidogen 2 (osteonidogen) | NM_001102065 | -30.3 | 5.7 | 10.6 |
| CLEC3B | C-type lectin domain family 3, member B | NM_001046212 | -30.5 | 5.7 | 10.7 |
| SNAI2 | snail homolog 2 (Drosophila) | NM_001034538 | -30.7 | 4.1 | 9.0 |
| GNB4 | guanine nucleotide binding protein (G protein), beta polypeptide 4 | NM_001099033 | -31.3 | 3.7 | 8.7 |
| EPAS1 | endothelial PAS domain protein 1 | NM_174725 | -31.9 | 5.6 | 10.6 |
| FRZB | frizzled-related protein | NM_174059 | -32.2 | 4.0 | 9.1 |
| COL6A1 | collagen, type VI, alpha 1 | NM_001143865 | -32.8 | 6.3 | 11.3 |
| PLXND1 | plexin D1 | XM_001789172 /// XM_002697122 | -33.4 | 4.1 | 9.2 |
| TGFBR2 | transforming growth factor, beta receptor II (70/80kDa) | NM_001159566 | -33.8 | 4.7 | 9.8 |
| A2M | alpha-2-macroglobulin | NM_001109795 | -34.6 | 5.3 | 10.4 |
| LOXL1 | lysyl oxidase-like 1 | NM_174383 | -34.7 | 3.9 | 9.0 |
| FBN1 | fibrillin 1 | NM_174053 | -34.8 | 5.7 | 10.8 |
| ACTN1 | actinin, alpha 1 | NM_001035351 | -35.0 | 5.1 | 10.2 |
| ENPP2 | ectonucleotide pyrophosphatase/phosphodiesterase 2 | NM_001080293 | -35.0 | 3.8 | 8.9 |
| RGS5 | regulator of G-protein signaling 5 | NM_001034707 | -35.4 | 4.4 | 9.5 |
| ITGBL1 | integrin, beta-like 1 (with EGF-like repeat domains) | NM_001206834 | -36.8 | 4.1 | 9.3 |
| TAGLN | transgelin | NM_001046149 | -37.2 | 5.5 | 10.7 |
| FAM101B | family with sequence similarity 101, member B | XM_002695711 /// XM_870793 | -38.3 | 4.4 | 9.7 |
| SCARA5 | scavenger receptor class A, member 5 (putative) | NM_001102499 | -39.8 | 3.4 | 8.7 |
| CLDN11 | claudin 11 | NM_001035055 | -40.3 | 4.2 | 9.6 |
| FLI1 | Friend leukemia virus integration 1 | NM_001046298 | -40.9 | 3.7 | 9.0 |
| LAMA2 /// LOC100848461 | laminin, alpha 2 /// laminin subunit alpha-2-like | XM_001787958 /// XM_002690220 /// | -41.1 | 3.6 | 9.0 |

| | | | | | |
|---|---|---|---|---|---|
| | | XM_003585167 /// XM_003585378 /// XM_003586475 | | | |
| GNG11 | guanine nucleotide binding protein (G protein), gamma 11 | NM_001024523 | -41.5 | 4.6 | 10.0 |
| RAMP2 | receptor (G protein-coupled) activity modifying protein 2 | NM_001098860 | -41.9 | 4.6 | 10.0 |
| FN1 | fibronectin 1 | NM_001163778 | -42.7 | 6.6 | 12.0 |
| PLK2 | polo-like kinase 2 | NM_001192245 | -44.7 | 3.5 | 9.0 |
| MMP2 | matrix metallopeptidase 2 (gelatinase A, 72kDa gelatinase, 72kDa type IV collagenase) | NM_174745 | -45.3 | 4.9 | 10.4 |
| CD99 | CD99 molecule | NM_001244214 | -46.8 | 4.5 | 10.1 |
| ADAMDEC1 /// LOC100847471 | ADAM-like, decysin 1 /// ADAM DEC1-like | NM_001206371 /// XM_002689785 /// XM_003582556 /// XM_003586413 /// XM_582254 | -47.1 | 3.5 | 9.0 |
| DCN | decorin | NM_173906 | -47.2 | 6.6 | 12.2 |
| XDH | xanthine dehydrogenase | NM_173972 | -49.0 | 4.7 | 10.3 |
| FILIP1L | filamin A interacting protein 1-like | XM_002684706 /// XM_002684707 /// XM_002702383 /// XM_003581681 /// XM_003581682 /// XM | -49.8 | 4.9 | 10.5 |
| CAV1 | caveolin 1, caveolae protein, 22kDa | NM_174004 | -50.5 | 4.4 | 10.0 |
| LHFP | lipoma HMGIC fusion partner | NM_001077990 | -56.0 | 4.5 | 10.3 |
| CXCL14 | chemokine (C-X-C motif) ligand 14 | NM_001034410 | -57.8 | 4.3 | 10.1 |
| SDPR | serum deprivation response | XM_002685467 /// XM_610845 | -57.8 | 4.0 | 9.8 |
| ADAMDEC1 | ADAM-like, decysin 1 | NM_001206371 /// XM_002689785 /// XM_582254 | -58.7 | 4.8 | 10.7 |
| LOC781493 | collagen alpha-1(XIV) chain-like | XM_003583071 /// XM_003586917 | -60.0 | 4.4 | 10.3 |
| IGF2 | insulin-like growth factor 2 (somatomedin A) | NM_174087 | -63.6 | 5.3 | 11.3 |

| | | | | | |
|---|---|---|---|---|---|
| HPGD | hydroxyprostaglandin dehydrogenase 15-(NAD) | NM_001034419 | -65.2 | 4.1 | 10.2 |
| COL6A3 | collagen, type VI, alpha 3 | XM_002686570 /// XM_002686571 /// XM_002686573 /// XM_003582037 /// XM_003582038 /// XM | -66.6 | 5.5 | 11.6 |
| ASPN | asporin | NM_001034309 | -69.4 | 4.6 | 10.7 |
| COL1A2 | collagen, type I, alpha 2 | NM_174520 | -69.5 | 6.8 | 12.9 |
| EGFLAM /// LOC100847583 | EGF-like, fibronectin type III and laminin G domains /// pikachurin-like | NM_001083478 /// XM_003587531 | -73.4 | 4.7 | 10.9 |
| SHISA2 | shisa homolog 2 (Xenopus laevis) | NM_001101265 | -75.5 | 3.9 | 10.1 |
| DUSP12 | dual specificity phosphatase 12 | XM_002685847 /// XM_581568 | -77.4 | 4.3 | 10.6 |
| OGN | osteoglycin | NM_173946 | -78.0 | 4.7 | 11.0 |
| APOD | apolipoprotein D | NM_001076301 | -84.7 | 4.9 | 11.3 |
| COL3A1 | collagen, type III, alpha 1 | NM_001076831 | -86.7 | 6.1 | 12.6 |
| FBLN5 | fibulin 5 | NM_001014946 | -88.8 | 4.4 | 10.9 |
| COL12A1 | collagen, type XII, alpha 1 | NM_001206497 | -90.1 | 4.4 | 10.9 |
| SPARCL1 | SPARC-like 1 (hevin) | NM_001034302 | -92.8 | 5.3 | 11.8 |
| RARRES1 | retinoic acid receptor responder (tazarotene induced) 1 | NM_001075430 | -99.8 | 4.3 | 10.9 |
| ACTA2 /// ACTG2 | actin, alpha 2, smooth muscle, aorta /// actin, gamma 2, smooth muscle, enteric | NM_001013592 /// NM_001034502 | -102.9 | 5.7 | 12.3 |
| COL5A2 | collagen, type V, alpha 2 | XM_003581798 /// XM_003585717 | -104.6 | 5.1 | 11.8 |
| MGP | matrix Gla protein | NM_174707 | -121.3 | 5.7 | 12.6 |
| ALDH1A1 | aldehyde dehydrogenase 1 family, member A1 | NM_174239 | -136.3 | 4.6 | 11.7 |
| COL15A1 | collagen, type XV, alpha 1 | NM_001191285 | -166.6 | 3.8 | 11.2 |
